# Serum and urinary golgi membrane protein 1 (GOLM1/GP73/G73) for chronic kidney disease staging

**DOI:** 10.3389/fmed.2026.1896678

**Published:** 2026-07-10

**Authors:** Jiaqi Xu, Jiawen Lin, Chao Ma, Jianishaya Yeerlan, Shuping Li, Wenjian Zhu, Zhihua Zheng, Mingcheng Huang

**Affiliations:** 1 Department of Nephrology, Center of Kidney and Urology, The Seventh Affiliated Hospital, Sun Yat-Sen University, Shenzhen, Guangdong, China; 2 Department of Surgical Anesthesia Center, The Seventh Affiliated Hospital, Sun Yat-Sen University, Shenzhen, Guangdong, China; 3 Department of Physiology, University of Oklahoma Health Sciences Center, Oklahoma City, OK, United States

**Keywords:** biomarker, chronic kidney disease, disease progression, GP73, serum, urine

## Abstract

**Background:**

In this cross-sectional observational study, we evaluated whether serum and urinary Golgi membrane protein 1 (GOLM1/GP73) could serve as biomarkers for CKD. Chronic kidney disease (CKD) lacks simple biomarkers reflecting both systemic burden and tubular health. GP73 is stress-responsive, but its utility across CKD and acute kidney injury (AKI) has not been systematically evaluated.

**Methods:**

We enrolled 32 healthy individuals and 172 kidney disease patients: CKD stages 1–3 (*n* = 70), 4–5 (*n* = 42), dialysis-dependent stage 5D (*n* = 40), and AKI or acute-on-chronic kidney disease (*n* = 20). Serum G73, urinary G73, urinary creatinine, and conventional renal markers were measured. Logistic regression, AUC, decision curve analysis (DCA), and ordinal regression were used. Kidney biopsies (*n* = 20) were immunostained for GP73.

**Results:**

Serum G73 progressively increased from healthy controls to CKD stages 4-5, with a modest decrease in CKD stage 5D (median: healthy 47.2, CKD1-3 62.2, CKD4-5 96.1, CKD5D 89.9 ng/mL; *P* < 0.001), whereas urinary G73 and the urine G73-to-creatinine ratio progressively declined (both *P* < 0.001). Adding serum G73 to a clinical model (age, sex, BMI, hypertension, diabetes, CKD duration) significantly improved discrimination of advanced CKD (CKD4-5/5D vs. 1–3): AUC increased from 0.81 to 0.85 (DeLong *P* = 0.026), with net DCA benefit across 0–85% thresholds. Adding serum G73 to a non-renal clinical covariate model improved discrimination for advanced CKD; however, sensitivity analyses showed no meaningful incremental value beyond eGFR or serum creatinine for this eGFR-defined endpoint. Serum G73 alone showed modest ability to distinguish AKI/AonC from CKD (AUC 0.59–0.75). Kidney GP73 immunostaining was weak and unchanged across stages.

**Conclusions:**

Serum G73 independently associates with CKD severity and adds diagnostic value to conventional models, especially for advanced CKD. The contrasting decline in urinary G73 and lack of increased renal tissue expression are consistent with a predominantly extra-renal contribution to circulating G73, while urinary G73 reflects tubular function. Serum and urinary G73 are useful non-invasive biomarkers that could improve CKD staging.

## 1 Introduction

Chronic kidney disease (CKD) affects approximately 10–15% of the global adult population and is a leading cause of morbidity, mortality, and healthcare expenditure worldwide (1–4). Early detection and accurate staging are critical for slowing disease progression, preventing complications, and preparing for renal replacement therapy (5). Currently, CKD staging relies primarily on estimated glomerular filtration rate (eGFR) derived from serum creatinine and on albuminuria (6). However, these markers have well-known limitations. Other candidate biomarkers, such as neutrophil gelatinase-associated lipocalin (NGAL) (7) and kidney injury molecule-1 (KIM-1) (8), have been investigated for assessing tubular injury. Serum creatinine is influenced by age, sex, muscle mass, and dietary protein intake, and its relationship with GFR is non-linear, making it relatively insensitive to early kidney function decline (9). Albuminuria, while a powerful prognosticator, is not universally present in all CKD subtypes, particularly in tubulointerstitial diseases (10). More importantly, neither marker directly reflects the integrity or function of the renal tubule- a compartment that plays a central role in solute reabsorption, secretion, and metabolic homeostasis (11). Tubular injury and dysfunction are increasingly recognized as key drivers of CKD progression and are associated with adverse outcomes independent of glomerular damage (12). Recent advances in metabolomics have greatly facilitated the discovery of novel biomarkers and the elucidation of disease mechanisms in various kidney diseases, including CKD, AKI, and diabetic kidney disease (13).Therefore, there is a pressing need for novel biomarkers that can complement eGFR and albuminuria by providing insights into both systemic disease burden and local tubular health.

Golgi membrane protein 1 (GOLM1, also known as GP73; hereinafter referred to as G73) is a type II Golgi transmembrane protein that is up-regulated in response to various cellular stresses, including inflammation, oxidative stress, and endoplasmic reticulum stress (14, 15). Initially identified as a serum biomarker for hepatocellular carcinoma and non-alcoholic steatohepatitis (16, 17), GP73 has also been extensively investigated for its diagnostic value in various liver diseases (18). In diabetic nephropathy, podocyte-specific GP73 overexpression exacerbates renal injury via the EGFR-PPARγ-AMPKα pathway, whereas GP73 ablation is protective (16). Moreover, serum GP73 levels are elevated in patients with diabetic kidney disease and correlate with renal dysfunction (16). Beyond diabetes, GP73 has been detected in urinary extracellular vesicles and may participate in intercellular communication within the kidney. These findings suggest that GP73 might serve as a dual-compartment biomarker-measurable in both serum and urine-that could capture both systemic stress and tubular functional changes. However, no study has systematically evaluated serum and urinary G73 across the full spectrum of predominantly non-diabetic CKD, from early stages to dialysis, nor compared its diagnostic performance with that of established clinical models in the same cohort.

In the present study, we aimed to characterize serum and urinary G73 levels across the full spectrum of kidney disease, including healthy individuals, patients with CKD stages 1–3, 4–5, and 5D, as well as those with AKI or acute-on-chronic kidney disease. We further sought to assess the diagnostic performance of serum G73 for distinguishing kidney disease from healthy status, identifying advanced CKD (stages 4–5/5D vs. 1–3), and discriminating acute from chronic kidney disease. Additionally, we evaluated whether adding serum G73 to a clinical model improves disease staging using discrimination (AUC), calibration, and decision curve analysis, and explored the relationship between serum/urinary G73 and renal tissue G73 expression in a subset of patients to gain mechanistic insight. By integrating serum and urinary measurements, we aimed to determine whether G73 could serve as a complementary biomarker reflecting both systemic illness and tubular function, thereby potentially improving CKD staging and clinical decision-making.

## 2 Materials and methods

### 2.1 Study design and participants

This observational study was conducted in the Department of Nephrology, Center of Kidney and Urology, The Seventh Affiliated Hospital, Sun Yat-Sen University (Shenzhen, Guangdong, China). Patients were enrolled between May 1, 2025 and February 6, 2026. The study was approved by the Ethics Committee of the hospital (approval number:KY-2025-015-01). All participants provided written informed consent. The study was reported in accordance with the Strengthening the Reporting of Observational Studies in Epidemiology (STROBE) statement (1) and the Standards for Reporting Diagnostic Accuracy Studies (STARD) criteria (2).

Healthy individuals were eligible if they had no history of kidney disease, normal renal function (estimated glomerular filtration rate [eGFR] ≥ 90 mL/min/1.73 m^2^), and no proteinuria on dipstick testing. Key exclusion criteria for healthy individuals included acute illness, known liver disease, malignancy, or use of nephrotoxic medications within the past 3 months.

Patients with kidney disease were included if they had a confirmed diagnosis based on Kidney Disease: Improving Global Outcomes (KDIGO) criteria, with available serum and urine samples at enrollment. Exclusion criteria for patients included active infection, pregnancy, or concomitant liver disease with ALT or AST > 3 times the upper limit of normal. A total of 32 healthy individuals and 172 patients with kidney disease were enrolled. Patients were categorized into five groups based on clinical diagnosis and kidney function: CKD stages 1–3 (*n* = 70); CKD stages 4–5 (*n* = 42); CKD stage 5D (dialysis-dependent, including hemodialysis and peritoneal dialysis, *n* = 40); AKI or AonC (*n* = 20). CKD staging was determined using the KDIGO 2024 criteria based on eGFR calculated by the CKD-EPI 2021 equation. AKI was defined according to the KDIGO criteria (increase in serum creatinine ≥0.3 mg/dL within 48 h, or ≥1.5 times baseline within 7 days, or urine output < 0.5 mL/kg/h for 6 h). AonC was defined as AKI superimposed on pre-existing CKD. Among the 172 patients with kidney disease, the most common etiologies of chronic kidney disease (CKD) were hypertensive nephropathy , diabetic kidney disease, IgA nephropathy , membranous nephropathy, and chronic glomerulonephritis. Other causes included obstructive nephropathy, focal segmental glomerulosclerosis, lupus nephritis, ANCA-associated vasculitis, gouty nephropathy, tubulointerstitial nephritis, drug-induced kidney injury, and genetic kidney diseases. Missing data for each variable are summarized in Appendix Table 1.

### 2.2 Sample and data collection

Fasting venous blood samples (5 mL) and midstream urine samples were collected from all participants at enrollment. Serum was separated by centrifugation at 3,000 rpm for 10 min and stored at −80°C until analysis. Urine samples were centrifuged at 1,500 rpm for 10 min, and the supernatant was stored at −80°C. All samples were processed within 2 h of collection. Serum and urine samples were available for all participants. No assays were repeated due to QC failures.

Demographic and clinical data were extracted from electronic medical records, including age, sex, body mass index (BMI), systolic and diastolic blood pressure (SBP/DBP), comorbidities (hypertension, diabetes, coronary heart disease, hyperlipidemia, cerebrovascular disease), CKD duration, dialysis status, and baseline medications (RAAS inhibitors, SGLT2 inhibitors, mineralocorticoid receptor antagonists). Laboratory parameters measured at enrollment included serum creatinine, urea, eGFR, cystatin C, uric acid, albumin, hemoglobin, platelet count, total cholesterol, triglycerides, LDL-C, HDL-C, fasting glucose, HbA1c, C-reactive protein (CRP), brain natriuretic peptide (BNP), ALT, AST, ferritin, calcium, phosphorus, parathyroid hormone, vitamin D, urine albumin-to-creatinine ratio (UACR), 24-h urinary protein, and urinary N-acetyl-β-D-glucosaminidase (NAG). For a subset of patients with available 6-month follow-up data (*n* = 76), serum creatinine, eGFR, UACR, and 24-h urinary protein were re-measured.

### 2.3 Biomarker assays

Serum and urine G73 concentrations were measured using a commercially available enzyme-linked immunosorbent assay (ELISA) kit (Human GP73 ELISA Kit, FineTest, Wuhan, China) according to the manufacturer's instructions. The kit measures total GP73/GOLM1. The intra-assay coefficients of variation were < 8%, and the inter-assay coefficients of variation were < 10%. All measurements were performed in duplicate, and the mean values were used for analysis. Urinary G73 levels were normalized to urinary creatinine to account for dilution variation, and the urine G73-to-creatinine ratio (U-G73/Cr) was calculated as: U-G73/Cr (ng/mg) = urine G73 (ng/mL)/urine creatinine (mg/dL). Personnel performing the biomarker assays were blinded to all clinical data.

### 2.4 Immunohistochemistry

Formalin-fixed, paraffin-embedded kidney sections (4 μm) were deparaffinized, rehydrated, and subjected to heat-induced antigen retrieval in Tris-EDTA buffer (pH 9.0). Endogenous peroxidase was blocked with 0.3% H_2_O_2_. Sections were incubated overnight at 4 °C with a rabbit monoclonal anti-GP73 antibody (Santa Cruz Biotechnology, Dallas, TX, USA; dilution 1:200), followed by HRP-conjugated goat anti-rabbit secondary antibody (dilution 1:200). Immunoreactivity was visualized using DAB, and sections were counterstained with hematoxylin. Stained sections were evaluated by two independent pathologists blinded to clinical data.

### 2.5 Statistical analysis

#### 2.5.1 General statistical framework

Statistical analyses were performed using R software version 4.2.1 (R Foundation for Statistical Computing, Vienna, Austria). This exploratory study aimed to evaluate the diagnostic and prognostic relevance of G73 in kidney disease. Descriptive statistics, visualization, and univariate analyses were first performed to characterize the distribution of clinical variables and G73-related biomarkers across predefined disease groups, including group comparisons, correlation analyses, univariate regression, and assessment of the discrimination, calibration, and clinical utility of serum G73 alone. Because of the exploratory nature of this study, candidate covariates were selected based on clinical relevance and prior evidence rather than data-driven variable selection methods such as LASSO regression. Sequential multivariable models were then constructed to evaluate the incremental predictive value of G73 combined with clinically relevant variables, with model performance assessed in terms of discrimination, calibration, and clinical utility. In addition, we explored whether G73 could serve as a potential prognostic predictor of renal outcomes at approximately 6 months. All statistical tests were two-sided unless otherwise specified, and a *P*-value < 0.05 was considered statistically significant. Missing data were handled using complete-case analysis for multivariable models. Sensitivity analyses using multiple imputation were performed to assess the robustness of the findings.

#### 2.5.2 Descriptive analysis and univariate analysis

Baseline demographic characteristics, clinical features, laboratory parameters, urinary biomarkers, comorbidities, medication use, and follow-up renal outcomes were summarized across predefined disease categories, including kidney disease status, CKD severity, acute versus chronic kidney disease status, and the integrated disease classification of healthy controls, CKD stages 1–3, CKD stages 4–5, CKD 5D, and AKI/AonC. The normality of continuous variables was assessed using the Shapiro-Wilk test. If Shapiro-Wilk test *P* value greater than 0.05, these variables were considered as Normally distributed variables. Normally distributed variables were presented as mean ± standard deviation and compared using Student's *t*-test or one-way analysis of variance, whereas non-normally distributed variables were presented as median with interquartile range and compared using the Wilcoxon rank-sum test or Kruskal–Wallis test, as appropriate. Categorical variables were summarized as counts and percentages and compared using Pearson's chi-square test, Fisher's exact test, or chi-square tests with simulated *P* values when applicable. The distributions of serum G73, urinary G73, and urinary G73-to-creatinine ratio were visualized using violin plots with embedded boxplots, individual data points, and median markers.

Before regression modeling, pairwise correlations among candidate regression variables, G73-related biomarkers, and 6-month renal outcome indicators were evaluated using Spearman's rank correlation analysis. Binary and categorical variables were numerically coded for correlation analyses, and multiple testing was adjusted using the Benjamini–Hochberg false discovery rate method. Correlation patterns were visualized using heatmaps.

#### 2.5.3 Multivariable analysis

Multivariable logistic regression models were used to evaluate the independent association between serum G73 and three binary endpoints: kidney disease vs. healthy status, advanced CKD, and acute vs. chronic kidney disease. Advanced CKD was defined as CKD stages 4–5 or CKD 5D, with CKD stages 1–3 as the reference group; AKI/AonC was defined as the event group in the acute vs. chronic kidney disease analysis. Sequential models were constructed for each endpoint. For kidney disease status, models included serum G73 alone, followed by adjustment for age, sex, and BMI, and further adjustment for ALT, AST, and ALB. For advanced CKD, models were sequentially adjusted for age, sex, BMI, hypertension, diabetes, CKD duration, ALT, AST, and ALB. For acute vs. chronic kidney disease, models were adjusted for age, sex, and BMI, with additional models incorporating either eGFR or serum creatinine. Because the AKI/AonC group had only 21 events, conventional logistic regression may produce biased estimates. Therefore, we used Firth penalized logistic regression to reduce small-sample bias. The events-per-variable (EPV) ratio for the fully adjusted model (Model 3b, with 4 predictors: age, sex, BMI, and serum creatinine) was approximately 5.25 (21 events/4 predictors), which is below the recommended minimum of 10, indicating a risk of overfitting. Therefore, the results for this endpoint should be interpreted as exploratory. Associations were reported as odds ratios with 95% confidence intervals, and percentile bootstrap confidence intervals were additionally estimated.

To explore the prognostic relevance of G73, linear regression models were fitted for changes in renal outcomes at approximately 6 months, including serum creatinine, eGFR, UACR, and 24-h urinary protein excretion. Changes were defined as follow-up values minus baseline values. Sequential models included disease group, serum G73, demographic variables, CKD duration, liver function indicators, comorbidities, baseline use of RAAS inhibitors, SGLT2 inhibitors, and mineralocorticoid receptor antagonists, and post-admission medication adjustments. Regression coefficients with 95% confidence intervals were reported, together with model fit indices including , adjusted , and Akaike information criterion (AIC).

#### 2.5.4 Discrimination, calibration and clinical utility

Model discrimination was assessed using the area under the receiver operating characteristic curve (AUC). Bootstrap 95% CIs were calculated using 1,000 resamples. The Youden index was used to determine the optimal cut-off value of serum G73 for each endpoint. Sensitivity, specificity, positive predictive value (PPV), and negative predictive value (NPV) were reported. The DeLong test was used to compare AUCs between models.

Calibration was evaluated using grouped calibration plots with 1,000 bootstrap resamples; logistic recalibration intercepts and slopes were estimated.

Clinical utility was assessed using decision curve analysis (DCA). Net benefit was calculated across threshold probabilities from 0.05 to 0.95, with treat-all and treat-none strategies as references. Bootstrap 95% CIs for net benefit were estimated with 1,000 resamples.

#### 2.5.5 Internal validation

Bootstrap optimism correction with 1,000 resamples was performed. Apparent AUC, optimism-corrected AUC, and corresponding calibration metrics were reported for each model.

## 3 Results

### 3.1 Baseline characteristics and G73 distribution

A total of 204 participants were included: 32 healthy individuals, 70 patients with CKD stages 1–3, 42 with CKD stages 4–5, 40 with dialysis-dependent CKD stage 5D, and 20 with AKI or acute-on-chronic kidney disease (AonC). Baseline characteristics are summarized in Table 1.

**Table 1:** Baseline characteristics and G73 profiles according to kidney disease category.

Characteristic	Healthy	CKD 1-3	CKD 4-5	CKD 5D	AKI/AonC	*P* value
	***N** = **32***	***N** = **70***	***N** = **42***	***N** = **40***	***N** = **20***	
Gender						0.757
Male	13 (56.5%)	42 (60.9%)	25 (61.0%)	28 (70.0%)	14 (70.0%)	
Female	10 (43.5%)	27 (39.1%)	16 (39.0%)	12 (30.0%)	6 (30.0%)	
Age	38.0 [25.5; 49.0]	46.0 [36.0; 61.0]	55.0 [43.0; 64.0]	55.0 [45.0; 62.2]	60.5 [50.8; 65.8]	< 0.001
BMI	23.5 [21.5; 24.7]	24.5 [22.1; 27.0]	24.3 [22.4; 27.4]	24.6 [23.0; 26.7]	25.0 [24.2; 27.9]	0.073
Systolic blood pressure	116 (12.0)	127 (19.2)	139 (20.2)	136 (21.9)	130 (17.9)	< 0.001
Diastolic Blood Pressure	73.0 [66.0; 79.5]	79.0 [72.0; 86.0]	83.0 [75.0; 96.0]	83.0 [73.5; 93.5]	80.0 [71.0; 90.5]	0.003
Serum G73	47.2 [36.4; 65.1]	62.2 [49.1; 81.7]	96.1 [71.6; 122]	89.9 [73.8; 125]	91.5 [80.0; 104]	< 0.001
Urine G73	17.2 [11.5; 28.0]	17.6 [10.9; 24.1]	7.56 [5.71; 10.2]	5.23 [3.74; 7.56]	15.3 [9.68; 16.7]	< 0.001
Urine G73/Urine creatinine	1.55 [1.02; 2.12]	1.76 [1.11; 2.65]	1.41 [0.97; 1.97]	0.82 [0.54; 1.17]	3.30 [1.72; 4.94]	< 0.001
Urea	4.60 [3.95; 5.60]	6.60 [4.80; 8.85]	17.4 [12.3; 22.6]	18.5 [15.1; 27.4]	15.4 [10.9; 22.7]	< 0.001
Creatinine	67.2 [55.6; 80.1]	98.5 [75.7; 123]	330 [261; 583]	833 [546; 1,109]	240 [154; 343]	< 0.001
eGFR	111 [104; 120]	72.0 [53.0; 96.0]	14.0 [10.0; 22.0]	6.00 [4.00; 9.00]	22.0 [17.8; 45.0]	< 0.001
Cystatin c		1.25 [1.04; 1.80]	3.49 [3.04; 4.35]	6.50 [4.90; 7.23]	2.56 [1.32; 2.78]	< 0.001
Uric acid	269 [232; 369]	339 [287;403]	425 [365;509]	350 [316;412]	431 [313;473]	< 0.001
Albumin-to-creatinine ratio	5.15 [3.31; 7.65]	386 [110; 1,690]	1,286 [465; 2,243]	2,903 [1,842; 5,938]	40.8 [35.3; 2,098]	0.002
24-h urinary total protein		732 [210; 2,273]	2,523 [1,019; 3,723]	4,835 [2,788; 6,414]	281 [137; 2,682]	< 0.001
Urine protein-to-creatinine ratio	0.01 [0.01; 0.02]	0.01 [0.01; 0.02]	0.01 [0.01; 0.02]	0.01 [0.00; 0.01]	0.03 [0.02; 0.05]	0.001
Urine NAG		7.68 [5.57; 14.2]	6.44 [5.00; 9.84]	6.92 [6.92; 6.92]	9.95 [6.35; 20.2]	0.724
Hypertension						< 0.001
No	0 (%)	33 (48.5%)	8 (19.5%)	3 (7.69%)	7 (38.9%)	
Yes	0 (%)	35 (51.5%)	33 (80.5%)	36 (92.3%)	11 (61.1%)	
Diabetes						0.044
No	0 (%)	55 (79.7%)	24 (58.5%)	22 (57.9%)	11 (61.1%)	
Yes	0 (%)	14 (20.3%)	17 (41.5%)	16 (42.1%)	7 (38.9%)	
Coronary heart disease						0.001
No	0 (%)	68 (98.6%)	39 (95.1%)	29 (76.3%)	17 (94.4%)	
Yes	0 (%)	1 (1.45%)	2 (4.88%)	9 (23.7%)	1 (5.56%)	
Hyperlipidemia						0.018
No	0 (%)	37 (53.6%)	28 (68.3%)	30 (78.9%)	15 (83.3%)	
Yes	0 (%)	32 (46.4%)	13 (31.7%)	8 (21.1%)	3 (16.7%)	
ALB	43.7 [42.7; 46.1]	37.5 [32.2; 40.0]	34.1 [32.3; 37.9]	35.4 [31.8; 38.2]	33.7 [28.1; 40.5]	< 0.001
ALT	18.3 [13.6; 24.7]	17.0 [11.9; 26.7]	16.4 [12.1; 36.2]	18.5 [10.9; 27.2]	19.6 [13.1; 32.9]	0.852
AST	18.5 [16.1; 20.6]	19.1 [15.1; 23.7]	18.7 [14.2; 26.2]	17.6 [11.1; 22.1]	20.3 [17.1; 29.7]	0.144

UPCR, urine protein-to-creatinine ratio (g/g); UACR, urine albumin-to-creatinine ratio (mg/g).

**Figure 1:**
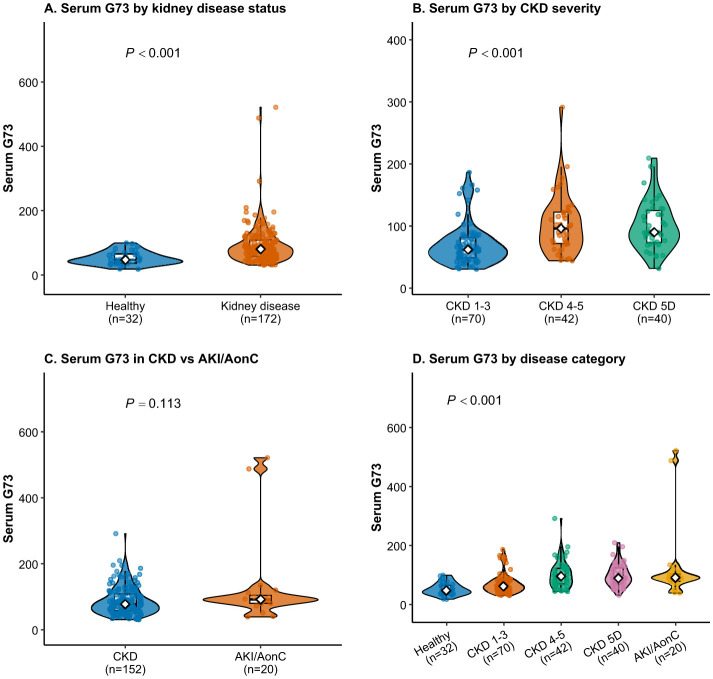
Distribution of serum G73 levels according to kidney disease status and disease category. **(A)**. Serum G73 by kidney disease status. **(B)**. Serum G73 by CKD severity. **(C)**. Serum G73 in CKD vs. AKI/AonC. **(D)**. Serum G73 by disease category. *P* values were derived from the Wilcoxon rank-sum test or Kruskal–Wallis test, as appropriate.

Age, systolic and diastolic blood pressure differed significantly across groups, whereas sex distribution and BMI were not significantly different. Serum G73 levels differed significantly across the five groups (Kruskal-Wallis *P* < 0.001), suggesting distinct urinary biomarker patterns according to disease severity and acute kidney disease status. The median serum G73 progressively increased from healthy controls [47.2 (36.4–65.1) ng/mL] to CKD stages 1–3 [62.2 (49.1–81.7) ng/mL] and further to CKD stages 4–5 [96.1 (71.6–122) ng/mL], remained elevated in CKD stage 5D [89.9 (73.8–125) ng/mL] and in AKI/AonC patients [91.5 (80.0–104) ng/mL] (Figures 1A,B). Pairwise comparisons showed that serum G73 was significantly higher in each patient group than in healthy controls (all *P* < 0.001). No significant difference was observed between CKD stage 5D and CKD stages 4–5 (Dunn's test, *P* = 0.797) nor between the combined CKD group and AKI/AonC group (Figure 1C, *P* = 0.11).

Urinary G73 and the urine G73-to-creatinine ratio also varied significantly across groups (both *P* < 0.001). Unlike serum G73, urinary G73 progressively declined with advancing CKD: median values were 17.2 ng/mL in healthy controls, 17.6 ng/mL in CKD 1–3, 7.56 ng/mL in CKD 4–5, and 5.23 ng/mL in CKD 5D. Of note, AKI/AonC patients exhibited higher urine G73-to-creatinine ratios (median 3.30 ng/mg) than all CKD subgroups, which is an exception to this declining trend (Figure 1D). The urine G73-to-creatinine ratio showed a similar decreasing trend, whereas AKI/AonC patients exhibited the highest ratio (median 3.30 ng/mg, *P* < 0.001 vs. healthy controls) (Figure 1D) (Appendix Figure 1 and Appendix Figure 2).

Conventional renal function markers (urea, serum creatinine, eGFR, cystatin C, uric acid, UACR, 24-h urinary protein) all showed expected differences across groups, confirming appropriate disease stratification (Table 1).

### 3.2 Correlations of G73 with clinical parameters

**Figure 2:**
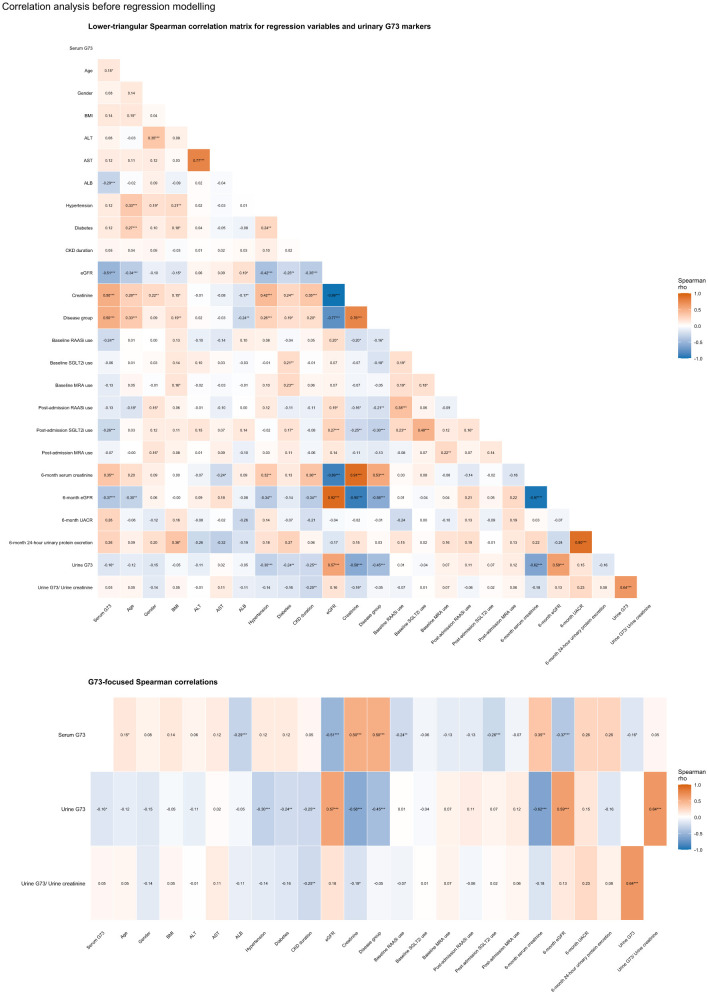
Correlation heatmap between G73 and other variables.

Spearman correlation analysis revealed that serum G73 was significantly correlated with eGFR (ρ = 0.51, *P* < 0.001), serum creatinine (ρ = 0.50, *P* < 0.001), ALB (ρ = 0.29, *P* < 0.001). In contrast, the urine G73-to-creatinine ratio did not correlate significantly with eGFR (ρ = 0.16, *P* = 0.059) but showed a moderate negative correlation with serum creatinine (ρ = 0.19, *P* = 0.028) (Appendix Table 2). Significant correlation was observed between serum G73 and urine G73 (ρ = 0.16, *P* = 0.035). But the correlation between serum G73 and the urine G73-to-creatinine ratio is not statistical significantly (ρ = 0.05, *P* = 0.576). A full correlation heatmap is provided in Figure 2.

### 3.3 Serum G73 for distinguishing kidney disease from healthy status

Logistic regression analyses showed that serum G73 was significantly associated with the presence of kidney disease in the unadjusted model (OR = 1.05, 95% CI: 1.01–1.09, *P* = 0.012) and after adjustment for age, sex and BMI (OR = 1.04, 95% CI: 1.01–1.09, *P* = 0.043). Further adjustment for ALT, AST and albumin attenuated the association (OR = 1.04, 95% CI: 1.00–1.10, *P* = 0.081) (Table 2). The attenuation after additional biochemical adjustment suggests that part of the association between serum G73 and kidney disease may be explained by related clinical or biochemical factors. Nevertheless, serum G73 remained positively associated with kidney disease in the crude and demographically adjusted models, supporting its potential relevance as a disease-associated biomarker.

**Table 2:** Logistic regression analyses of serum G73 for identifying kidney disease, advanced CKD, and AKI/AonC.

Endpoint	Model	Term	Estimate	OR 95%CI	*P* value
Healthy vs. kidney disease	Model 1: Serum G73	Serum G73	1.05	1.05 (1.01–1.09)	0.012
Healthy vs. kidney disease	Model 2: Model 1 + age + sex + BMI	Serum G73	1.04	1.04 (1.01–1.09)	0.043
Healthy vs. kidney disease	Model 2: Model 1 + age + sex + BMI	Age	1.08	1.08 (1.03–1.14)	0.004
Healthy vs. kidney disease	Model 2: Model 1 + age + sex + BMI	Gender (ref: Male)	3.43	3.43 (0.69–26.79)	0.168
Healthy vs. kidney disease	Model 2: Model 1 + age + sex + BMI	BMI	1.06	1.06 (0.86–1.31)	0.595
Healthy vs. kidney disease	Model 3: Model 2 + ALT + AST + ALB	Serum G73	1.04	1.04 (1.00–1.10)	0.081
Healthy vs. kidney disease	Model 3: Model 2 + ALT + AST + ALB	Age	1.04	1.04 (0.95–1.14)	0.416
Healthy vs. kidney disease	Model 3: Model 2 + ALT + AST + ALB	Gender (ref: Male)	1.57	1.57 (0.13–23.81)	0.722
Healthy vs. kidney disease	Model 3: Model 2 + ALT + AST + ALB	BMI	1.14	1.14 (0.79–1.78)	0.514
Healthy vs. kidney disease	Model 3: Model 2 + ALT + AST + ALB	ALT	0.98	0.98 (0.86–1.17)	0.774
Healthy vs. kidney disease	Model 3: Model 2 + ALT + AST + ALB	AST	1.02	1.02 (0.82–1.23)	0.843
Healthy vs. kidney disease	Model 3: Model 2 + ALT + AST + ALB	ALB	0.58	0.58 (0.35–0.79)	0.006
CKD1-3 vs. CKD4-5/5D	Model 1: Serum G73	Serum G73	1.02	1.02 (1.01–1.03)	< 0.001
CKD1-3 vs. CKD4-5/5D	Model 2: Model 1+ age + sex + BMI	Serum G73	1.02	1.02 (1.01–1.03)	< 0.001
CKD1-3 vs. CKD4-5/5D	Model 2: Model 1+ age + sex + BMI	Age	1.04	1.04 (1.01–1.07)	0.010
CKD1-3 vs. CKD4-5/5D	Model 2: Model 1+ age + sex + BMI	Gender (ref: Male)	0.77	0.77 (0.35–1.72)	0.527
CKD1-3 vs. CKD4-5/5D	Model 2: Model 1+ age + sex + BMI	BMI	1.06	1.06 (0.95–1.19)	0.335
CKD1-3 vs. CKD4-5/5D	Model 3: Model 2+ hypertension + diabetes + CKD duration	Serum G73	1.02	1.02 (1.01–1.04)	< 0.001
CKD1-3 vs. CKD4-5/5D	Model 3: Model 2+ hypertension + diabetes + CKD duration	Age	1.02	1.02 (0.99–1.05)	0.284
CKD1-3 vs. CKD4-5/5D	Model 3: Model 2+ hypertension + diabetes + CKD duration	Gender (ref: Male)	1.10	1.10 (0.46–2.72)	0.830
CKD1-3 vs. CKD4-5/5D	Model 3: Model 2+ hypertension + diabetes + CKD duration	BMI	1.01	1.01 (0.89–1.15)	0.845
CKD1-3 vs. CKD4-5/5D	Model 3: Model 2+ hypertension + diabetes + CKD duration	Hypertension	4.77	4.77 (1.80–13.75)	0.002
CKD1-3 vs. CKD4-5/5D	Model 3: Model 2+ hypertension + diabetes + CKD duration	Diabetes	1.83	1.83 (0.70–4.97)	0.224
CKD1-3 vs. CKD4-5/5D	Model 3: Model 2+ hypertension + diabetes + CKD duration	CKD duration	1.08	1.08 (1.00–1.18)	0.071
CKD1-3 vs. CKD4-5/5D	Model 4: Model 3+ ALT + AST + ALB	Serum G73	1.03	1.03 (1.02–1.04)	< 0.001
CKD1-3 vs. CKD4-5/5D	Model 4: Model 3+ ALT + AST + ALB	Age	1.03	1.03 (0.99–1.07)	0.132
CKD1-3 vs. CKD4-5/5D	Model 4: Model 3+ ALT + AST + ALB	Gender (ref: Male)	1.40	1.40 (0.53–3.80)	0.500
CKD1-3 vs. CKD4-5/5D	Model 4: Model 3+ ALT + AST + ALB	BMI	0.99	0.99 (0.87–1.14)	0.920
CKD1-3 vs. CKD4-5/5D	Model 4: Model 3+ ALT + AST + ALB	Hypertension	5.55	5.55 (1.95–17.61)	0.002
CKD1-3 vs. CKD4-5/5D	Model 4: Model 3+ ALT + AST + ALB	Diabetes	1.53	1.53 (0.56–4.31)	0.415
CKD1-3 vs. CKD4-5/5D	Model 4: Model 3+ ALT + AST + ALB	CKD duration	1.08	1.08 (1.00–1.18)	0.082
CKD1-3 vs. CKD4-5/5D	Model 4: Model 3+ ALT + AST + ALB	ALT	1.05	1.05 (1.01–1.11)	0.039
CKD1-3 vs. CKD4-5/5D	Model 4: Model 3+ ALT + AST + ALB	AST	0.92	0.92 (0.85–0.98)	0.014
CKD1-3 vs. CKD4-5/5D	Model 4: Model 3+ ALT + AST + ALB	ALB	0.99	0.99 (0.91–1.08)	0.772
CKD vs. AKI/AonC	Model 1: Serum G73	Serum G73	1.01	1.01 (1.00–1.01)	0.133
CKD vs. AKI/AonC	Model 2: Model 1+ age + sex + BMI	Serum G73	1.01	1.01 (1.00–1.02)	0.057
CKD vs. AKI/AonC	Model 2: Model 1 + age + sex + BMI	Age	1.04	1.04 (1.00–1.08)	0.065
CKD vs. AKI/AonC	Model 2: Model 1 + age + sex + BMI	Gender (ref: Male)	0.65	0.65 (0.19–1.90)	0.450
CKD vs. AKI/AonC	Model 2: Model 1 + age + sex + BMI	BMI	1.10	1.10 (0.95–1.28)	0.205
CKD vs. AKI/AonC	Model 3a: Model 2+ eGFR	Serum G73	1.01	1.01 (1.00–1.02)	0.056
CKD vs. AKI/AonC	Model 3a: Model 2+ eGFR	Age	1.04	1.04 (1.00–1.09)	0.063
CKD vs. AKI/AonC	Model 3a: Model 2+ eGFR	Gender (ref: Male)	0.64	0.64 (0.19–1.88)	0.439
CKD vs. AKI/AonC	Model 3a: Model 2+ eGFR	BMI	1.11	1.11 (0.95–1.29)	0.189
CKD vs. AKI/AonC	Model 3a: Model 2+ eGFR	eGFR	1.00	1.00 (0.99–1.02)	0.694
CKD vs. AKI/AonC	Model 3b: Model 2+ creatinine	Serum G73	1.01	1.01 (1.00–1.02)	0.010
CKD vs. AKI/AonC	Model 3b: Model 2+ creatinine	Age	1.05	1.05 (1.01–1.09)	0.025
CKD vs. AKI/AonC	Model 3b: Model 2+ creatinine	Gender (ref: Male)	0.48	0.48 (0.13–1.46)	0.215
CKD vs. AKI/AonC	Model 3b: Model 2+ creatinine	BMI	1.17	1.17 (1.00–1.40)	0.058
CKD vs. AKI/AonC	Model 3b: Model 2+ creatinine	Creatinine	1.00	1.00 (0.99–1.00)	0.019

The ROC curve for serum G73 alone yielded an AUC of 0.79 (95% CI: 0.69–0.88). Adding age, sex and BMI increased the AUC to 0.88 (95% CI: 0.77–0.98), and the fully adjusted model (including ALT, AST and albumin) achieved an AUC of 0.97 (95% CI: 0.94–0.99). At the optimal Youden threshold, the fully adjusted model showed 88% sensitivity and 100% specificity. For the healthy-vs.-kidney-disease endpoint, serum G73 showed apparent discriminatory ability. However, this analysis should be interpreted as exploratory because the common complete-case dataset included only 10 healthy controls. In the fully adjusted model, the non-event-per-variable ratio was 1.43, indicating a high risk of overfitting. Although the apparent AUC was 0.97, bootstrap internal validation suggested optimism, with an optimism-corrected AUC of 0.92 and a calibration slope of 0.38. The detailed discriminative performance metrics (AUC, optimal cutoff, sensitivity, specificity, PPV, NPV) for this endpoint are shown in Table 3. Bootstrap estimates for the logistic regression models are shown in Appendix Table 4.

**Table 3:** Discriminative performance of serum G73-based logistic regression models for kidney disease-related endpoints.

Endpoint	Model	AUC	AUC 95%CI	Cutoff	Sensitivity	Specificity	PPV	NPV
Healthy vs. kidney disease	Model 1	0.79	0.79 (0.69–0.88)	0.94	0.67	0.90	0.99	0.15
Healthy vs. kidney disease	Model 2	0.88	0.88 (0.77–0.98)	0.94	0.81	0.80	0.98	0.21
Healthy vs. kidney disease	Model 3	0.97	0.97 (0.94–0.99)	0.95	0.88	1.00	1.00	0.36
CKD1-3 vs. CKD4-5/5D	Model 1	0.74	0.74 (0.65–0.83)	0.48	0.81	0.62	0.73	0.72
CKD1-3 vs. CKD4-5/5D	Model 2	0.76	0.76 (0.67–0.84)	0.65	0.56	0.91	0.89	0.62
CKD1-3 vs. CKD4-5/5D	Model 3	0.81	0.81 (0.73–0.88)	0.63	0.70	0.81	0.82	0.68
CKD1-3 vs. CKD4-5/5D	Model 4	0.85	0.85 (0.77–0.91)	0.56	0.84	0.76	0.81	0.79
CKD vs. AKI/AonC	Model 1	0.59	0.59 (0.46–0.71)	0.11	0.79	0.52	0.18	0.95
CKD vs. AKI/AonC	Model 2	0.71	0.71 (0.57–0.83)	0.14	0.74	0.71	0.25	0.95
CKD vs. AKI/AonC	Model 3a	0.71	0.71 (0.57–0.84)	0.14	0.68	0.75	0.27	0.95
CKD vs. AKI/AonC	Model 3b	0.75	0.75 (0.63–0.87)	0.22	0.53	0.91	0.43	0.94

The cutoff value refers to the predicted probability threshold from the logistic model, not the serum G73 concentration.

### 3.4 Serum G73 for identifying advanced CKD

For distinguishing advanced CKD (CKD stages 4–5 or 5D) from early CKD (stages 1–3), serum G73 showed a consistent association across all logistic regression models. In the unadjusted model, higher serum G73 was associated with advanced CKD (OR = 1.02, 95% CI: 1.01–1.03, *P* < 0.001). This association remained significant after sequential adjustment for age, sex, BMI, hypertension, diabetes, CKD duration, ALT, AST and albumin (OR = 1.03, 95% CI: 1.02–1.04, *P* < 0.001, Model 4 in Table 2).

**Figure 3:**
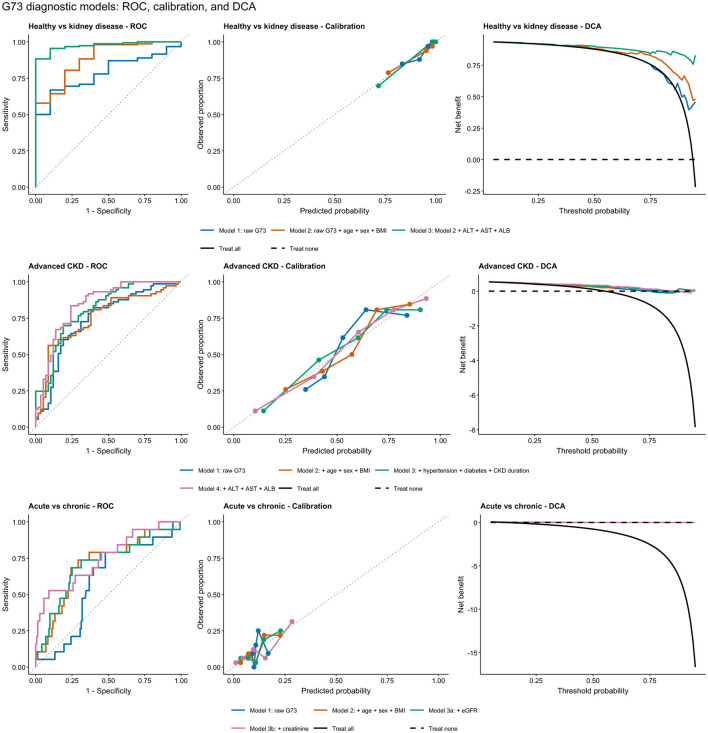
Discrimination, calibration, and clinical utility of serum G73-based models for kidney disease-related endpoints. The three panels in the first row show the ROC curve, calibration curve, and decision curve analysis (DCA) curve of the G73-based prediction models for distinguishing healthy controls from patients with kidney disease. The three panels in the second row show the ROC curve, calibration curve, and DCA curve of the G73-based prediction models for identifying advanced CKD. The three panels in the third row show the ROC curve, calibration curve, and DCA curve of the G73-based prediction models for distinguishing CKD from AKI/AonC. Shaded bands in the calibration plots represent 95% confidence intervals estimated by bootstrap resampling.

The G73-only model gave an AUC of 0.74 (95% CI: 0.65–0.83). Adding clinical covariates (age, sex, BMI) increased the AUC to 0.76, and further adjustment for hypertension, diabetes and CKD duration raised it to 0.81. The fully adjusted model (Model 4) achieved the highest AUC of 0.85 (95% CI: 0.77–0.91), with sensitivity 84%, specificity 76%, PPV 81% and NPV 79% at the optimal threshold. Compared with the clinical model (Model 3, AUC = 0.81), adding serum G73 (Model 4, AUC = 0.85) significantly improved discrimination (DeLong test, *P* = 0.026). Calibration was acceptable, and DCA showed that the model including G73 provided a higher net benefit across a wide range of threshold probabilities (0%−85%) (Figure 3).The full discriminative performance of all models for identifying advanced CKD is presented in Table 3.

In sensitivity analyses requested to assess incremental value beyond established renal-function markers, models including eGFR or serum creatinine were nearly saturated for the eGFR-defined advanced CKD endpoint. Adding serum G73 to these models did not meaningfully improve AUC, IDI, or decision-curve net benefit. In contrast, serum G73 improved discrimination when added to a non-renal clinical covariate model, suggesting that G73 is associated with CKD severity but does not add substantial information beyond eGFR or creatinine for this endpoint. The results of sensitivity analyses were shown in Appendix Tables 7, 8, 9.

### 3.5 Serum G73 for distinguishing AKI/AonC from CKD

For differentiating AKI/AonC from chronic kidney disease (CKD/CKD 5D), using Firth penalized logistic regression to reduce small-sample bias, serum G73 was not significantly associated in the unadjusted model (OR = 1.01, 95% CI: 1.00–1.01, *P* = 0.133) or after adjustment for age, sex and BMI (OR = 1.01, 95% CI: 1.00–1.02, *P* = 0.057). The association became statistically significant only after further adjustment for serum creatinine (OR = 1.01, 95% CI: 1.00–1.02, *P* = 0.010) (Table 2).

The G73-only model showed limited discriminative ability (AUC = 0.59, 95% CI: 0.46–0.71). Adding age, sex and BMI increased the AUC to 0.71 (95% CI: 0.57–0.83), and adjustment for creatinine further improved it to 0.75 (95% CI: 0.63–0.87), with specificity 91% and NPV 94% albeit modest sensitivity (53%). DCA revealed no substantial net benefit over the treat-none strategy (Figure 3). The corresponding performance metrics for distinguishing AKI/AonC from CKD are also shown in Table 3. Given the small number of AKI/AonC events (*n* = 21), these findings should be considered exploratory. The events-per-variable (EPV) ratio for the creatinine-adjusted model was approximately 5.25, which is below the recommended minimum of 10, indicating a risk of overfitting. Therefore, these results require validation in larger independent cohorts.

### 3.6 Exploratory 6-month follow-up analysis

**Figure 4:**
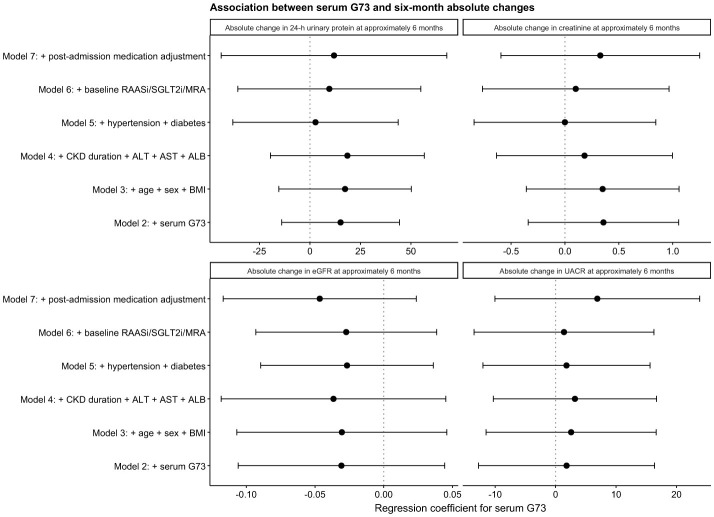
Forest map of regression coefficient for serum in six-month absolute changes models.

Six-month follow-up data were available for 76 patients. Median changes in eGFR and serum creatinine were modest (−1.0 mL/min/1.73 m^2^ and +3.85 μmol/L, respectively), and only two patients (3%) reached an eGFR decline ≥30%. Linear regression analyses showed no significant association between baseline serum G73 and 6-month changes in eGFR, serum creatinine, UACR or 24-h urinary protein after adjustment for baseline covariates (all *P* > 0.05). These null findings are likely explained by the short follow-up duration and the relatively stable disease course in this cohort, and they do not diminish the cross-sectional diagnostic value of serum G73 for disease staging. Forest plots of regression coefficients are provided in Figure 4. Regression results were shown in Appendix Table 3.

### 3.7 Immunohistochemistry

**Figure 5:**
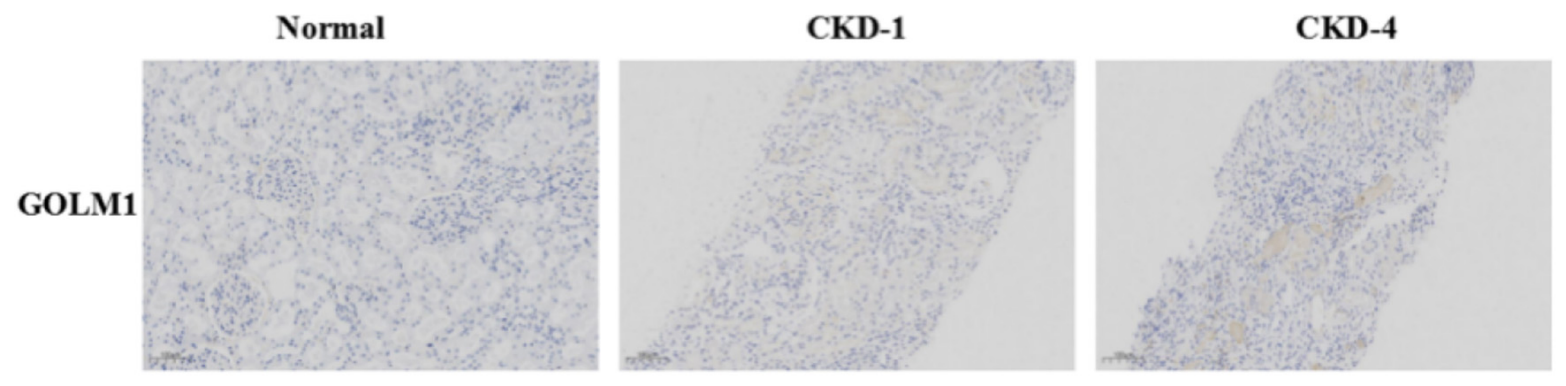
Representative images of GP73 immunohistochemistry in human kidney tissues.

RenalGP73 expression was evaluated by immunohistochemistry in 4 normal controls, 7 patients with CKD stages 1–2, and 9 patients with CKD stages 3–4. Positive staining was predominantly localized to the cytoplasm of tubular epithelial cells, with minimal or absent staining in glomeruli. Staining intensity (0–3) and the percentage of positively stained tubules (0–4) were scored semi-quantitatively, and the product (0–12) was used as the final score. The median GP73 IHC score was 1.5 in normal controls, 2.0 in CKD stages 1–2, and 2.5 in CKD stages 3–4. Although scores tended to be higher in advanced CKD, no statistically significant difference was observed among the three groups (Kruskal-Wallis test, *P* = 0.12). Representative images are shown in Figure 5.

These findings indicate that renal GP73 expression does not substantially increase with CKD progression, suggesting that the elevated serum G73 levels in CKD patients are not primarily derived from increased local production within the kidney.

## 4 Discussion

In this cross-sectional observational study, we systematically evaluated the clinical utility of serum and urinary G73 across the full spectrum of kidney disease, including healthy individuals, non-dialysis CKD stages 1–3 and 4–5, dialysis-dependent CKD stage 5D, and patients with AKI or acute-on-chronic kidney disease. The high apparent AUC observed for the healthy-vs.-kidney-disease endpoint should be interpreted cautiously. Healthy controls were younger than patients, and the complete-case analysis included a very small number of non-events. The low non-event-per-variable ratio and reduced optimism-corrected calibration slope indicate potential overfitting. Therefore, this analysis supports an association between serum G73 and kidney-disease status but does not establish a clinically deployable diagnostic model. The main findings can be summarized as follows: serum G73 levels progressively increased with CKD severity from healthy to CKD 4-5, with a modest decrease in CKD 5D, whereas urinary G73 and the urine G73-to-creatinine ratio progressively declined; serum G73 alone showed moderate discrimination for advanced CKD (AUC 0.74), but adding it to a clinical model significantly improved the AUC to 0.85 (DeLong *P* = 0.026) with a favorable net benefit on decision curve analysis. Serum G73 improved discrimination beyond non-renal clinical covariates, but did not provide meaningful additional discrimination beyond eGFR or serum creatinine in the eGFR-defined advanced CKD endpoint. This suggests that serum G73 should not be interpreted as a replacement for eGFR or creatinine in CKD staging. Rather, it may reflect CKD severity and systemic disease burden, with potential biological relevance that requires further validation. The association between serum G73 and AKI/AonC was modest and reached significance only after adjustment for serum creatinin. Renal GP73 immunostaining was weak and unchanged across CKD stages, but this finding is limited by small sample size (*n* = 20), non-significant difference (*P* = 0.12), and possible low antibody sensitivity. Thus, these data generate a hypothesis (extra-renal origin) rather than proving it, and do not firmly exclude increased local production. Taken together, these results position serum G73 as a useful biomarker for CKD staging that adds independent diagnostic value to conventional clinical models, while the contrasting serum-urine pattern and the lack of increased tissue expression suggest that circulating G73 originates mainly from extra-renal sources and that urinary G73 may serve as a functional readout of tubular health. Although serum G73 correlated significantly with eGFR (ρ = −0.51, *P* < 0.001) and cystatin C (ρ = 0.52, *P* < 0.001), the moderate magnitude of these correlation coefficients suggests that G73 captures only part of the overall disease burden.

Our observation that serum G73 is elevated in CKD patients and correlates with disease severity extends previous findings in diabetic nephropathy. Xu et al. reported that serumGP73 levels were higher in patients with diabetic kidney disease and correlated positively with serum creatinine and negatively with eGFR (16). In their mouse model, podocyte-specific GP73 overexpression exacerbated diabetes-induced renal injury, whereas podocyte-specific ablation was protective. The present study expands these observations to a predominantly non-diabetic CKD population and, for the first time, includes patients with AKI and AonC. Moreover, while Xu et al. focused on podocytes, our work provides evidence that tubular G73 dynamics differ from those in the glomerular compartment- a distinction that may have important pathophysiological implications.

In contrast to serum G73, urinary G73 and the urine G73-to-creatinine ratio were lower in patients with advanced CKD and CKD 5D than in healthy controls and early-stage CKD patients. This pattern is reminiscent of certain tubular proteins whose excretion declines as tubular mass and function are lost. For example, urinary α-1-microglobulin and β-2-microglobulin, markers of proximal tubular reabsorption, typically increase with tubular injury (11); however, G73 appears to behave differently - its urinary excretion decreases in advanced disease. This discrepancy may reflect distinct biological roles: G73 is a Golgi-associated protein that can be released into the extracellular space via exosomes or secretory pathways, and its urinary concentration may depend on the integrity of tubular secretion rather than on passive leakage. We acknowledge that urinary biomarker concentrations can also be influenced by urine flow, dilution, proteinuria, and changes in glomerular filtration. Although normalization to urinary creatinine mitigates these effects, residual confounding cannot be excluded. Therefore, the interpretation of declining urinary G73 as isolated tubular dysfunction or impaired secretion remains speculative, and alternative explanations (e.g., reduced renal clearance, altered tubular handling) should be considered. Future studies using urinary exosome analysis or direct measurement of tubular secretion are needed to clarify the underlying mechanisms. To our knowledge, this is the first study to simultaneously measure serum and urinary G73 across the entire CKD spectrum and to relate these measurements to kidney tissue expression. Compared with established tubular injury markers (NGAL, KIM-1, α1-microglobulin), G73 offers the advantage of dual-compartment measurement (serum and urine) and may reflect loss of tubular secretory capacity rather than injury. However, G73 is less validated in kidney disease, and its urinary concentration may be influenced by dilution and flow. Thus, G73 should be considered a complementary rather than a replacement marker, and further comparative studies are needed.

One of the most intriguing observations of this study is the divergent trend between serum and urinary G73: serum levels increased with advancing CKD, whereas urinary levels decreased. This “scissors” pattern was further highlighted by immunohistochemistry, which showed only weak GP73 expression in renal tubules and glomeruli without any significant change across CKD stages. This “scissors” pattern should be considered hypothesis-generating rather than a proven mechanism, and further studies are needed to elucidate the underlying biology. Collectively, these findings argue against the simple hypothesis that elevated serum G73 originates from increased local production within the diseased kidney. Instead, we propose two non-mutually exclusive mechanisms. One possibility is that circulating G73 derives partly from extra-renal sources (e.g., liver), supported by hepatic G73 expression (14, 15) and attenuation after adjusting for liver enzymes. However, an alternative explanation is that as GFR declines, serum G73 rises due to reduced renal clearance, while urinary G73 falls with tubular loss. Our data cannot distinguish between these mechanisms. Thus, the “extra-renal source” is one hypothesis among several, requiring further study. Second, the decline in urinary G73, in the absence of a corresponding drop in tissue G73, suggests a defect in tubular secretion or exosomal packaging rather than a loss of cellular G73 content. Tubular cells in advanced CKD undergo dedifferentiation, atrophy, and dysfunction of their secretory machinery (11). G73 is known to be released in association with extracellular vesicles; thus, impaired vesicle biogenesis or trafficking could reduce urinary G73 excretion while tissue levels remain unchanged. This interpretation aligns with the concept that the urine G73-to-creatinine ratio may serve as a functional biomarker of tubular health-a parameter not captured by serum G73 or eGFR.

From a practical perspective, serum G73 alone showed only moderate ability to discriminate healthy individuals from patients with kidney disease (AUC = 0.79) or early from advanced CKD (AUC = 0.74). However, when added to a clinical model containing age, sex, BMI, hypertension, diabetes, and CKD duration, serum G73 significantly improved the AUC to 0.84 (95% CI: 0.77–0.91). The DeLong test confirmed that this improvement was statistically significant (*P* = 0.026). More importantly, decision curve analysis demonstrated that the model including serum G73 provided a higher net benefit than the clinical model alone across a wide range of threshold probabilities (0%−85%), indicating that using G73 to guide decisions would reduce unnecessary interventions or missed diagnoses. These results support the potential use of serum G73 as an adjunctive tool to refine disease staging for advanced CKD, particularly in settings where more precise staging is needed, such as in clinical trials or before making decisions about dialysis preparation.

In contrast, the performance of serum G73 for distinguishing AKI/AonC from CKD was limited (AUC = 0.59–0.75), and the association was significant only after adjusting for serum creatinine. Moreover, the number of patients with acute presentations was small (*n* = 21), and the wide confidence intervals indicate imprecision. Therefore, we caution against using serum G73 in isolation to differentiate acute from chronic kidney disease; its role in this setting, if any, remains exploratory and requires validation in larger cohorts.

This study has several strengths. First, the inclusion of a well-characterized cohort covering the entire spectrum of kidney disease- from healthy individuals to non-dialysis CKD, dialysis-dependent CKD, AKI, and AonC-allowed us to examine G73 dynamics across different disease categories. Second, the simultaneous measurement of serum and urinary G73, together with a subset of kidney biopsies, provided multi-compartment evidence that is rarely available in biomarker studies. Third, the statistical approach was rigorous, including bootstrap optimism correction for AUC, calibration plots, decision curve analysis, and appropriate handling of non-normal distributions.

Several limitations must be acknowledged. First, this was a singlecenter study, which may limit the generalizability of our findings. Second, the healthy control group was significantly younger than the patient groups (median 38 vs. 46–63 years). Although age was adjusted for in all multivariable models, residual confounding cannot be completely excluded. Third, the sample size for the AKI/AonC group was small (*n* = 21), and the models for this endpoint should be considered exploratory. The events-per-variable ratio was approximately 5.25, below the recommended minimum of 10, indicating a risk of overfitting. Firth penalized logistic regression was used to reduce small-sample bias, but independent validation is needed. Fourth, we did not perform external validation in an independent cohort. Fifth, the absence of measured GFR (mGFR) prevented us from directly comparing G73 with a gold-standard reference. We used eGFR derived from serum creatinine, which is subject to the limitations inherent in creatinine-based equations. Sixth, the immunohistochemical analysis was performed on a limited number of biopsies and was semi-quantitative; the weak and uniform staining could be due to low antibody sensitivity or true low expression. A quantitative method such as digital image analysis or RNA in situ hybridization might provide additional resolution. Seventh, we did not measure other potential confounders such as liver fibrosis markers or circulating inflammatory cytokines that could influence serum G73 levels. Eighth, the 6-month follow-up duration was relatively short, and longer follow-up is needed to assess hard outcomes. Finally, the cross-sectional design precludes causal inferences; longitudinal studies are needed to determine whether baseline G73 predicts future eGFR decline.

Our findings generate several hypotheses for future research. First, the origin of circulating G73 in CKD should be investigated using animal models with tissue-specific G73 deletion (e.g., liver- vs. kidney-specific knockout) and by measuring portal vs. systemic G73 levels in patients. Second, the mechanism underlying the decline in urinary G73 deserves further exploration, particularly whether it reflects reduced tubular secretion, altered exosomal sorting, or decreased viable tubular mass. Third, prospective cohort studies with longer follow-up are warranted to evaluate whether baseline serum or urinary G73 predicts hard endpoints such as ESKD or cardiovascular events beyond traditional risk factors. Fourth, the incremental value of combining serum and urinary G73 (e.g., a G73-based score) should be tested in independent external cohorts.

## 5 Conclusions

In summary, Serum G73 is associated with CKD severity and may improve classification beyond non-renal clinical variables, but its incremental value beyond eGFR or creatinine was not demonstrated for the eGFR-defined advanced CKD endpoint. Given the cross-sectional design and the largely negative 6-month follow-up, G73 should be considered a candidate biomarker associated with CKD severity rather than a validated prognostic marker. The contrasting patterns of serum and urinary G73, together with the lack of increased renal tissue expression, are consistent with a possible extra-renal contribution, but reduced renal clearance remains an alternative explanation that requires further investigation. Urinary G73 appears to reflect tubular function. These findings position G73 as a promising dual-compartment biomarker that could be used alongside eGFR and albuminuria in the management of CKD. However, validation in larger, prospective, multi-center cohorts is required before clinical implementation. For the differentiation of acute from chronic kidney disease, the utility of serum G73 appears limited and requires further investigation.

## Data Availability

The original contributions presented in the study are included in the article/Supplementary material, further inquiries can be directed to the corresponding authors.
