# Supplementary material for: Serum and urinary golgi membrane protein 1 (GOLM1/GP73/G73) for chronic kidney disease staging
Source: Front Med (Lausanne). 2026 Jul 10;13:1896678. doi: 10.3389/fmed.2026.1896678 (PMC13395760; doi:10.3389/fmed.2026.1896678)
Supplement: Supplementary file 1 [file Supplementary_File_1.docx]

**Supplementary Materials:** **Additional Figures and Tables**

**Appendix Table1. Missing variables summary**

| variable | n | No missing | Missing\ | Missing percent |
| --- | --- | --- | --- | --- |
| Disease group | 207 | 207 | 0 | 0.00 |
| Kidney disease status | 207 | 207 | 0 | 0.00 |
| Acute kidney disease status | 207 | 175 | 32 | 15.46 |
| CKD severity | 207 | 154 | 53 | 25.60 |
| Gender | 207 | 194 | 13 | 6.28 |
| Age | 207 | 194 | 13 | 6.28 |
| BMI | 207 | 191 | 16 | 7.73 |
| Systolic blood pressure | 207 | 192 | 15 | 7.25 |
| Diastolic Blood Pressure | 207 | 192 | 15 | 7.25 |
| Serum G73 | 207 | 204 | 3 | 1.45 |
| Urine G73 | 207 | 171 | 36 | 17.39 |
| Urine creatinine | 207 | 160 | 47 | 22.71 |
| Urine G73/ Urine creatinine | 207 | 144 | 63 | 30.43 |
| Urea | 207 | 189 | 18 | 8.70 |
| Creatinine | 207 | 192 | 15 | 7.25 |
| eGFR | 207 | 192 | 15 | 7.25 |
| Cystatin c | 207 | 136 | 71 | 34.30 |
| Uric acid | 207 | 171 | 36 | 17.39 |
| Albumin-to-Creatinine Ratio | 207 | 104 | 103 | 49.76 |
| 24-hour urinary total protein | 207 | 133 | 74 | 35.75 |
| Urine Protein-to-Creatinine Ratio | 207 | 142 | 65 | 31.40 |
| Urine NAG | 207 | 66 | 141 | 68.12 |
| Hypertension | 207 | 167 | 40 | 19.32 |
| Diabetes | 207 | 167 | 40 | 19.32 |
| Coronary heart disease | 207 | 167 | 40 | 19.32 |
| Hyperlipidemia | 207 | 167 | 40 | 19.32 |
| Fasting Blood Glucose | 207 | 143 | 64 | 30.92 |
| HbA1c | 207 | 93 | 114 | 55.07 |
| TG | 207 | 171 | 36 | 17.39 |
| LDL-C | 207 | 171 | 36 | 17.39 |
| HDL-C | 207 | 171 | 36 | 17.39 |
| CRP | 207 | 56 | 151 | 72.95 |
| hsCRP | 207 | 61 | 146 | 70.53 |
| Hemoglobin | 207 | 190 | 17 | 8.21 |
| ALB | 207 | 171 | 36 | 17.39 |
| Ferritin | 207 | 82 | 125 | 60.39 |
| ALT | 207 | 186 | 21 | 10.14 |
| AST | 207 | 186 | 21 | 10.14 |
| BNP | 207 | 80 | 127 | 61.35 |
| EF | 207 | 101 | 106 | 51.21 |
| CKD duration | 207 | 160 | 47 | 22.71 |
| Dialysis modality | 207 | 168 | 39 | 18.84 |


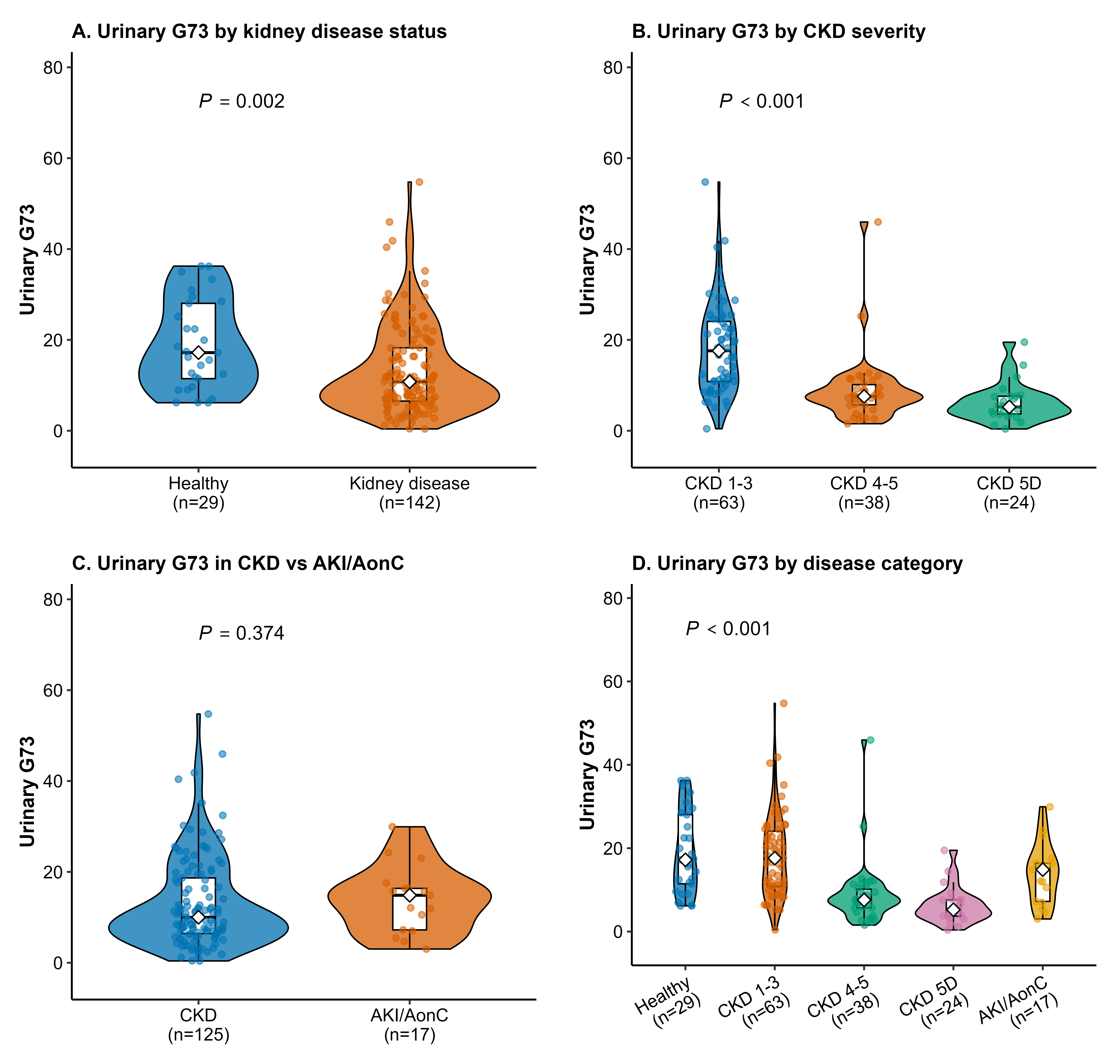


**Appendix Figure 1. Distribution of Urinary G73 levels according to kidney disease status and disease category.**


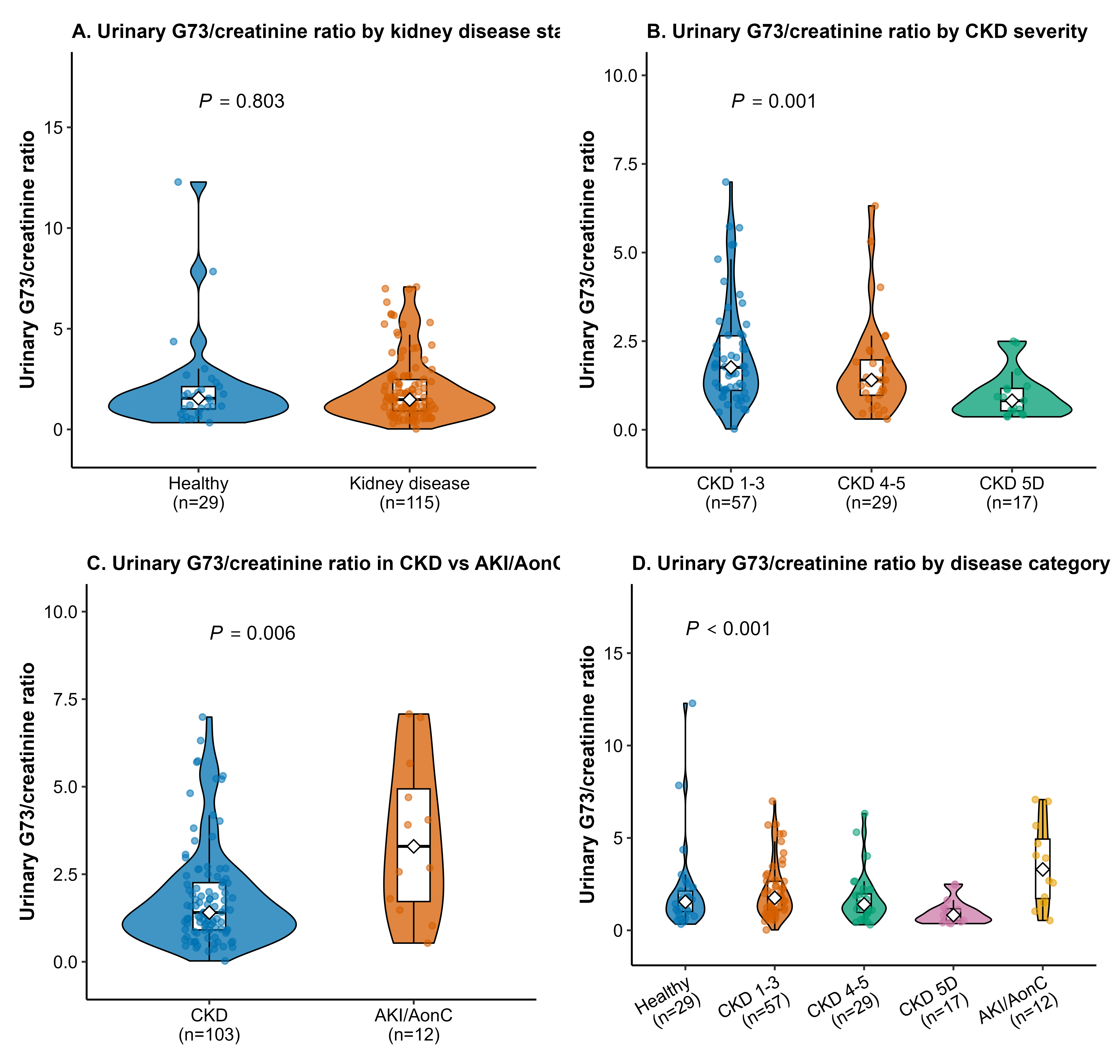


**Appendix Figure 2. Distribution of Urinary G73/creatinine ratio levels according to kidney disease status and disease category.**

**Appendix Table 2. Correlations between serum G73 and other variables.**

| **variable_1** | **variable_2** | **n** | **method** | $\boldsymbol{\rho}$ | ***P*-value** | ***P* -adj BH** |
| --- | --- | --- | --- | --- | --- | --- |
| Serum G73 | Age | 193 | spearman | 0.15 | 0.043 | 0.145 |
| Serum G73 | Gender | 193 | spearman | 0.08 | 0.299 | 0.525 |
| Serum G73 | BMI | 190 | spearman | 0.14 | 0.054 | 0.168 |
| Serum G73 | ALT | 185 | spearman | 0.06 | 0.396 | 0.609 |
| Serum G73 | AST | 185 | spearman | 0.12 | 0.104 | 0.267 |
| Serum G73 | ALB | 170 | spearman | -0.29 | <0.001 | 0.001 |
| Serum G73 | Hypertension | 166 | spearman | 0.12 | 0.112 | 0.283 |
| Serum G73 | Diabetes | 166 | spearman | 0.12 | 0.111 | 0.283 |
| Serum G73 | CKD duration | 160 | spearman | 0.05 | 0.512 | 0.701 |
| Serum G73 | eGFR | 191 | spearman | -0.51 | <0.001 | <0.001 |
| Serum G73 | Creatinine | 191 | spearman | 0.50 | <0.001 | <0.001 |
| Serum G73 | Disease group | 204 | spearman | 0.50 | <0.001 | <0.001 |
| Serum G73 | Baseline RAASi use | 171 | spearman | -0.24 | 0.002 | 0.011 |
| Serum G73 | Baseline SGLT2i use | 171 | spearman | -0.06 | 0.404 | 0.612 |
| Serum G73 | Baseline MRA use | 171 | spearman | -0.13 | 0.099 | 0.257 |
| Serum G73 | Post-admission RAASi use | 171 | spearman | -0.13 | 0.097 | 0.256 |
| Serum G73 | Post-admission SGLT2i use | 171 | spearman | -0.26 | 0.001 | 0.005 |
| Serum G73 | Post-admission MRA use | 171 | spearman | -0.07 | 0.352 | 0.584 |
| Serum G73 | 6-month serum creatinine | 77 | spearman | 0.35 | 0.002 | 0.011 |
| Serum G73 | 6-month eGFR | 77 | spearman | -0.37 | 0.001 | 0.006 |
| Serum G73 | 6-month UACR | 53 | spearman | 0.26 | 0.064 | 0.193 |
| Serum G73 | 6-month 24-hour urinary protein excretion | 31 | spearman | 0.26 | 0.153 | 0.343 |
| Serum G73 | Urine G73 | 168 | spearman | -0.16 | 0.035 | 0.130 |
| Serum G73 | Urine G73/ Urine creatinine | 144 | spearman | 0.05 | 0.576 | 0.748 |
| Age | Gender | 194 | spearman | 0.14 | 0.054 | 0.168 |
| Age | BMI | 191 | spearman | 0.14 | 0.047 | 0.154 |
| Age | ALT | 186 | spearman | -0.03 | 0.671 | 0.819 |
| Age | AST | 186 | spearman | 0.10 | 0.157 | 0.348 |
| Age | ALB | 171 | spearman | -0.01 | 0.866 | 0.938 |
| Age | Hypertension | 167 | spearman | 0.32 | <0.001 | <0.001 |
| Age | Diabetes | 167 | spearman | 0.28 | <0.001 | 0.002 |
| Age | CKD duration | 160 | spearman | 0.04 | 0.604 | 0.777 |
| Age | eGFR | 190 | spearman | -0.34 | <0.001 | <0.001 |
| Age | Creatinine | 190 | spearman | 0.28 | <0.001 | 0.001 |
| Age | Disease group | 194 | spearman | 0.33 | <0.001 | <0.001 |
| Age | Baseline RAASi use | 170 | spearman | 0.01 | 0.928 | 0.960 |
| Age | Baseline SGLT2i use | 170 | spearman | 0.01 | 0.913 | 0.953 |
| Age | Baseline MRA use | 170 | spearman | 0.05 | 0.502 | 0.695 |
| Age | Post-admission RAASi use | 170 | spearman | -0.18 | 0.018 | 0.072 |
| Age | Post-admission SGLT2i use | 170 | spearman | 0.03 | 0.722 | 0.849 |
| Age | Post-admission MRA use | 170 | spearman | -0.00 | 0.954 | 0.974 |
| Age | 6-month serum creatinine | 77 | spearman | 0.20 | 0.089 | 0.244 |
| Age | 6-month eGFR | 77 | spearman | -0.30 | 0.009 | 0.042 |
| Age | 6-month UACR | 53 | spearman | -0.06 | 0.671 | 0.819 |
| Age | 6-month 24-hour urinary protein excretion | 31 | spearman | 0.09 | 0.642 | 0.802 |
| Age | Urine G73 | 160 | spearman | -0.12 | 0.124 | 0.305 |
| Age | Urine G73/ Urine creatinine | 137 | spearman | 0.05 | 0.535 | 0.717 |
| Gender | BMI | 191 | spearman | 0.04 | 0.609 | 0.777 |
| Gender | ALT | 186 | spearman | 0.34 | <0.001 | <0.001 |
| Gender | AST | 186 | spearman | 0.11 | 0.123 | 0.305 |
| Gender | ALB | 171 | spearman | 0.09 | 0.223 | 0.431 |
| Gender | Hypertension | 167 | spearman | 0.18 | 0.020 | 0.082 |
| Gender | Diabetes | 167 | spearman | 0.11 | 0.158 | 0.348 |
| Gender | CKD duration | 160 | spearman | 0.05 | 0.503 | 0.695 |
| Gender | eGFR | 190 | spearman | -0.11 | 0.139 | 0.333 |
| Gender | Creatinine | 190 | spearman | 0.22 | 0.002 | 0.013 |
| Gender | Disease group | 194 | spearman | 0.10 | 0.186 | 0.382 |
| Gender | Baseline RAASi use | 170 | spearman | -0.00 | 0.984 | 0.987 |
| Gender | Baseline SGLT2i use | 170 | spearman | 0.03 | 0.687 | 0.824 |
| Gender | Baseline MRA use | 170 | spearman | -0.01 | 0.847 | 0.936 |
| Gender | Post-admission RAASi use | 170 | spearman | 0.16 | 0.042 | 0.144 |
| Gender | Post-admission SGLT2i use | 170 | spearman | 0.11 | 0.141 | 0.333 |
| Gender | Post-admission MRA use | 170 | spearman | 0.16 | 0.039 | 0.139 |
| Gender | 6-month serum creatinine | 77 | spearman | 0.09 | 0.413 | 0.616 |
| Gender | 6-month eGFR | 77 | spearman | 0.06 | 0.596 | 0.771 |
| Gender | 6-month UACR | 53 | spearman | -0.12 | 0.378 | 0.597 |
| Gender | 6-month 24-hour urinary protein excretion | 31 | spearman | 0.20 | 0.287 | 0.510 |
| Gender | Urine G73 | 160 | spearman | -0.16 | 0.049 | 0.159 |
| Gender | Urine G73/ Urine creatinine | 137 | spearman | -0.14 | 0.099 | 0.257 |
| BMI | ALT | 183 | spearman | 0.09 | 0.202 | 0.402 |
| BMI | AST | 183 | spearman | 0.03 | 0.693 | 0.824 |
| BMI | ALB | 168 | spearman | -0.09 | 0.268 | 0.484 |
| BMI | Hypertension | 164 | spearman | 0.21 | 0.007 | 0.035 |
| BMI | Diabetes | 164 | spearman | 0.18 | 0.024 | 0.094 |
| BMI | CKD duration | 158 | spearman | -0.03 | 0.753 | 0.872 |
| BMI | eGFR | 187 | spearman | -0.15 | 0.040 | 0.140 |
| BMI | Creatinine | 187 | spearman | 0.15 | 0.036 | 0.132 |
| BMI | Disease group | 191 | spearman | 0.18 | 0.010 | 0.047 |
| BMI | Baseline RAASi use | 167 | spearman | 0.13 | 0.092 | 0.248 |
| BMI | Baseline SGLT2i use | 167 | spearman | 0.14 | 0.064 | 0.193 |
| BMI | Baseline MRA use | 167 | spearman | 0.16 | 0.041 | 0.140 |
| BMI | Post-admission RAASi use | 167 | spearman | 0.06 | 0.404 | 0.612 |
| BMI | Post-admission SGLT2i use | 167 | spearman | 0.11 | 0.151 | 0.343 |
| BMI | Post-admission MRA use | 167 | spearman | 0.07 | 0.337 | 0.568 |
| BMI | 6-month serum creatinine | 77 | spearman | 0.00 | 0.970 | 0.979 |
| BMI | 6-month eGFR | 77 | spearman | -0.00 | 0.974 | 0.981 |
| BMI | 6-month UACR | 53 | spearman | 0.16 | 0.252 | 0.463 |
| BMI | 6-month 24-hour urinary protein excretion | 31 | spearman | 0.36 | 0.049 | 0.159 |
| BMI | Urine G73 | 158 | spearman | -0.05 | 0.567 | 0.743 |
| BMI | Urine G73/ Urine creatinine | 135 | spearman | 0.05 | 0.574 | 0.748 |
| ALT | AST | 186 | spearman | 0.77 | <0.001 | <0.001 |
| ALT | ALB | 168 | spearman | 0.01 | 0.856 | 0.938 |
| ALT | Hypertension | 160 | spearman | 0.03 | 0.742 | 0.862 |
| ALT | Diabetes | 160 | spearman | 0.04 | 0.648 | 0.803 |
| ALT | CKD duration | 153 | spearman | 0.01 | 0.912 | 0.953 |
| ALT | eGFR | 185 | spearman | 0.06 | 0.387 | 0.602 |
| ALT | Creatinine | 185 | spearman | -0.02 | 0.794 | 0.902 |
| ALT | Disease group | 186 | spearman | 0.01 | 0.844 | 0.936 |
| ALT | Baseline RAASi use | 162 | spearman | -0.10 | 0.207 | 0.409 |
| ALT | Baseline SGLT2i use | 162 | spearman | 0.11 | 0.181 | 0.382 |
| ALT | Baseline MRA use | 162 | spearman | -0.02 | 0.842 | 0.936 |
| ALT | Post-admission RAASi use | 162 | spearman | -0.01 | 0.939 | 0.964 |
| ALT | Post-admission SGLT2i use | 162 | spearman | 0.15 | 0.051 | 0.162 |
| ALT | Post-admission MRA use | 162 | spearman | 0.01 | 0.860 | 0.938 |
| ALT | 6-month serum creatinine | 73 | spearman | -0.07 | 0.537 | 0.717 |
| ALT | 6-month eGFR | 73 | spearman | 0.09 | 0.469 | 0.668 |
| ALT | 6-month UACR | 49 | spearman | -0.08 | 0.609 | 0.777 |
| ALT | 6-month 24-hour urinary protein excretion | 31 | spearman | -0.26 | 0.162 | 0.355 |
| ALT | Urine G73 | 154 | spearman | -0.10 | 0.199 | 0.399 |
| ALT | Urine G73/ Urine creatinine | 133 | spearman | -0.01 | 0.918 | 0.953 |
| AST | ALB | 168 | spearman | -0.04 | 0.565 | 0.743 |
| AST | Hypertension | 160 | spearman | -0.02 | 0.802 | 0.905 |
| AST | Diabetes | 160 | spearman | -0.06 | 0.432 | 0.632 |
| AST | CKD duration | 153 | spearman | 0.02 | 0.794 | 0.902 |
| AST | eGFR | 185 | spearman | 0.10 | 0.184 | 0.382 |
| AST | Creatinine | 185 | spearman | -0.09 | 0.225 | 0.432 |
| AST | Disease group | 186 | spearman | -0.04 | 0.622 | 0.791 |
| AST | Baseline RAASi use | 162 | spearman | -0.14 | 0.081 | 0.227 |
| AST | Baseline SGLT2i use | 162 | spearman | 0.03 | 0.689 | 0.824 |
| AST | Baseline MRA use | 162 | spearman | -0.03 | 0.695 | 0.824 |
| AST | Post-admission RAASi use | 162 | spearman | -0.09 | 0.247 | 0.457 |
| AST | Post-admission SGLT2i use | 162 | spearman | 0.07 | 0.363 | 0.595 |
| AST | Post-admission MRA use | 162 | spearman | 0.09 | 0.270 | 0.485 |
| AST | 6-month serum creatinine | 73 | spearman | -0.24 | 0.043 | 0.145 |
| AST | 6-month eGFR | 73 | spearman | 0.18 | 0.125 | 0.306 |
| AST | 6-month UACR | 49 | spearman | -0.02 | 0.917 | 0.953 |
| AST | 6-month 24-hour urinary protein excretion | 31 | spearman | -0.32 | 0.078 | 0.225 |
| AST | Urine G73 | 154 | spearman | 0.03 | 0.700 | 0.826 |
| AST | Urine G73/ Urine creatinine | 133 | spearman | 0.11 | 0.192 | 0.390 |
| ALB | Hypertension | 158 | spearman | -0.01 | 0.909 | 0.953 |
| ALB | Diabetes | 158 | spearman | -0.07 | 0.413 | 0.616 |
| ALB | CKD duration | 151 | spearman | 0.03 | 0.679 | 0.821 |
| ALB | eGFR | 169 | spearman | 0.18 | 0.020 | 0.081 |
| ALB | Creatinine | 169 | spearman | -0.16 | 0.037 | 0.132 |
| ALB | Disease group | 171 | spearman | -0.23 | 0.003 | 0.016 |
| ALB | Baseline RAASi use | 160 | spearman | 0.09 | 0.262 | 0.478 |
| ALB | Baseline SGLT2i use | 160 | spearman | -0.04 | 0.633 | 0.798 |
| ALB | Baseline MRA use | 160 | spearman | -0.02 | 0.849 | 0.936 |
| ALB | Post-admission RAASi use | 160 | spearman | -0.00 | 0.952 | 0.974 |
| ALB | Post-admission SGLT2i use | 160 | spearman | 0.13 | 0.094 | 0.253 |
| ALB | Post-admission MRA use | 160 | spearman | -0.10 | 0.217 | 0.423 |
| ALB | 6-month serum creatinine | 71 | spearman | 0.09 | 0.431 | 0.632 |
| ALB | 6-month eGFR | 71 | spearman | -0.08 | 0.512 | 0.701 |
| ALB | 6-month UACR | 48 | spearman | -0.26 | 0.075 | 0.218 |
| ALB | 6-month 24-hour urinary protein excretion | 29 | spearman | -0.19 | 0.327 | 0.557 |
| ALB | Urine G73 | 138 | spearman | -0.06 | 0.497 | 0.694 |
| ALB | Urine G73/ Urine creatinine | 116 | spearman | -0.11 | 0.231 | 0.441 |
| Hypertension | Diabetes | 166 | spearman | 0.22 | 0.005 | 0.024 |
| Hypertension | CKD duration | 157 | spearman | 0.10 | 0.232 | 0.441 |
| Hypertension | eGFR | 163 | spearman | -0.41 | <0.001 | <0.001 |
| Hypertension | Creatinine | 163 | spearman | 0.41 | <0.001 | <0.001 |
| Hypertension | Disease group | 167 | spearman | 0.24 | 0.001 | 0.010 |
| Hypertension | Baseline RAASi use | 166 | spearman | 0.09 | 0.263 | 0.478 |
| Hypertension | Baseline SGLT2i use | 166 | spearman | -0.00 | 0.962 | 0.975 |
| Hypertension | Baseline MRA use | 166 | spearman | 0.10 | 0.180 | 0.382 |
| Hypertension | Post-admission RAASi use | 166 | spearman | 0.13 | 0.095 | 0.253 |
| Hypertension | Post-admission SGLT2i use | 166 | spearman | -0.01 | 0.882 | 0.944 |
| Hypertension | Post-admission MRA use | 166 | spearman | 0.03 | 0.658 | 0.812 |
| Hypertension | 6-month serum creatinine | 76 | spearman | 0.32 | 0.005 | 0.028 |
| Hypertension | 6-month eGFR | 76 | spearman | -0.34 | 0.003 | 0.016 |
| Hypertension | 6-month UACR | 52 | spearman | 0.14 | 0.326 | 0.557 |
| Hypertension | 6-month 24-hour urinary protein excretion | 31 | spearman | 0.18 | 0.345 | 0.579 |
| Hypertension | Urine G73 | 135 | spearman | -0.29 | 0.001 | 0.004 |
| Hypertension | Urine G73/ Urine creatinine | 114 | spearman | -0.14 | 0.150 | 0.343 |
| Diabetes | CKD duration | 157 | spearman | 0.02 | 0.792 | 0.902 |
| Diabetes | eGFR | 163 | spearman | -0.26 | 0.001 | 0.007 |
| Diabetes | Creatinine | 163 | spearman | 0.24 | 0.002 | 0.011 |
| Diabetes | Disease group | 167 | spearman | 0.20 | 0.008 | 0.039 |
| Diabetes | Baseline RAASi use | 166 | spearman | -0.05 | 0.558 | 0.738 |
| Diabetes | Baseline SGLT2i use | 166 | spearman | 0.21 | 0.007 | 0.035 |
| Diabetes | Baseline MRA use | 166 | spearman | 0.23 | 0.003 | 0.019 |
| Diabetes | Post-admission RAASi use | 166 | spearman | -0.11 | 0.148 | 0.343 |
| Diabetes | Post-admission SGLT2i use | 166 | spearman | 0.16 | 0.039 | 0.138 |
| Diabetes | Post-admission MRA use | 166 | spearman | 0.10 | 0.182 | 0.382 |
| Diabetes | 6-month serum creatinine | 75 | spearman | 0.13 | 0.276 | 0.493 |
| Diabetes | 6-month eGFR | 75 | spearman | -0.14 | 0.245 | 0.456 |
| Diabetes | 6-month UACR | 52 | spearman | -0.07 | 0.637 | 0.800 |
| Diabetes | 6-month 24-hour urinary protein excretion | 30 | spearman | 0.27 | 0.150 | 0.343 |
| Diabetes | Urine G73 | 136 | spearman | -0.24 | 0.004 | 0.024 |
| Diabetes | Urine G73/ Urine creatinine | 115 | spearman | -0.16 | 0.088 | 0.244 |
| CKD duration | eGFR | 156 | spearman | -0.35 | <0.001 | <0.001 |
| CKD duration | Creatinine | 156 | spearman | 0.35 | <0.001 | <0.001 |
| CKD duration | Disease group | 160 | spearman | 0.20 | 0.012 | 0.050 |
| CKD duration | Baseline RAASi use | 159 | spearman | 0.05 | 0.558 | 0.738 |
| CKD duration | Baseline SGLT2i use | 159 | spearman | -0.01 | 0.887 | 0.944 |
| CKD duration | Baseline MRA use | 159 | spearman | 0.06 | 0.445 | 0.646 |
| CKD duration | Post-admission RAASi use | 159 | spearman | -0.11 | 0.187 | 0.382 |
| CKD duration | Post-admission SGLT2i use | 159 | spearman | -0.08 | 0.306 | 0.534 |
| CKD duration | Post-admission MRA use | 159 | spearman | -0.06 | 0.449 | 0.647 |
| CKD duration | 6-month serum creatinine | 73 | spearman | 0.36 | 0.002 | 0.011 |
| CKD duration | 6-month eGFR | 73 | spearman | -0.34 | 0.003 | 0.017 |
| CKD duration | 6-month UACR | 51 | spearman | -0.21 | 0.135 | 0.327 |
| CKD duration | 6-month 24-hour urinary protein excretion | 30 | spearman | 0.06 | 0.756 | 0.873 |
| CKD duration | Urine G73 | 129 | spearman | -0.25 | 0.004 | 0.023 |
| CKD duration | Urine G73/ Urine creatinine | 111 | spearman | -0.25 | 0.009 | 0.042 |
| eGFR | Creatinine | 192 | spearman | -0.99 | <0.001 | <0.001 |
| eGFR | Disease group | 192 | spearman | -0.77 | <0.001 | <0.001 |
| eGFR | Baseline RAASi use | 168 | spearman | 0.20 | 0.009 | 0.043 |
| eGFR | Baseline SGLT2i use | 168 | spearman | 0.07 | 0.352 | 0.584 |
| eGFR | Baseline MRA use | 168 | spearman | 0.07 | 0.379 | 0.597 |
| eGFR | Post-admission RAASi use | 168 | spearman | 0.20 | 0.011 | 0.047 |
| eGFR | Post-admission SGLT2i use | 168 | spearman | 0.27 | <0.001 | 0.004 |
| eGFR | Post-admission MRA use | 168 | spearman | 0.14 | 0.079 | 0.225 |
| eGFR | 6-month serum creatinine | 76 | spearman | -0.89 | <0.001 | <0.001 |
| eGFR | 6-month eGFR | 76 | spearman | 0.92 | <0.001 | <0.001 |
| eGFR | 6-month UACR | 52 | spearman | -0.04 | 0.764 | 0.878 |
| eGFR | 6-month 24-hour urinary protein excretion | 31 | spearman | -0.17 | 0.363 | 0.595 |
| eGFR | Urine G73 | 158 | spearman | 0.57 | <0.001 | <0.001 |
| eGFR | Urine G73/ Urine creatinine | 133 | spearman | 0.16 | 0.059 | 0.182 |
| Creatinine | Disease group | 192 | spearman | 0.76 | <0.001 | <0.001 |
| Creatinine | Baseline RAASi use | 168 | spearman | -0.20 | 0.010 | 0.045 |
| Creatinine | Baseline SGLT2i use | 168 | spearman | -0.07 | 0.396 | 0.609 |
| Creatinine | Baseline MRA use | 168 | spearman | -0.07 | 0.367 | 0.595 |
| Creatinine | Post-admission RAASi use | 168 | spearman | -0.16 | 0.035 | 0.130 |
| Creatinine | Post-admission SGLT2i use | 168 | spearman | -0.25 | 0.001 | 0.007 |
| Creatinine | Post-admission MRA use | 168 | spearman | -0.11 | 0.143 | 0.336 |
| Creatinine | 6-month serum creatinine | 76 | spearman | 0.91 | <0.001 | <0.001 |
| Creatinine | 6-month eGFR | 76 | spearman | -0.90 | <0.001 | <0.001 |
| Creatinine | 6-month UACR | 52 | spearman | -0.02 | 0.882 | 0.944 |
| Creatinine | 6-month 24-hour urinary protein excretion | 31 | spearman | 0.15 | 0.408 | 0.615 |
| Creatinine | Urine G73 | 158 | spearman | -0.58 | <0.001 | <0.001 |
| Creatinine | Urine G73/ Urine creatinine | 133 | spearman | -0.19 | 0.028 | 0.108 |
| Disease group | Baseline RAASi use | 172 | spearman | -0.17 | 0.030 | 0.117 |
| Disease group | Baseline SGLT2i use | 172 | spearman | -0.19 | 0.014 | 0.061 |
| Disease group | Baseline MRA use | 172 | spearman | -0.05 | 0.492 | 0.692 |
| Disease group | Post-admission RAASi use | 172 | spearman | -0.21 | 0.005 | 0.026 |
| Disease group | Post-admission SGLT2i use | 172 | spearman | -0.30 | <0.001 | 0.001 |
| Disease group | Post-admission MRA use | 172 | spearman | -0.13 | 0.081 | 0.227 |
| Disease group | 6-month serum creatinine | 77 | spearman | 0.53 | <0.001 | <0.001 |
| Disease group | 6-month eGFR | 77 | spearman | -0.58 | <0.001 | <0.001 |
| Disease group | 6-month UACR | 53 | spearman | -0.01 | 0.935 | 0.964 |
| Disease group | 6-month 24-hour urinary protein excretion | 31 | spearman | 0.03 | 0.881 | 0.944 |
| Disease group | Urine G73 | 171 | spearman | -0.45 | <0.001 | <0.001 |
| Disease group | Urine G73/ Urine creatinine | 144 | spearman | -0.05 | 0.538 | 0.717 |
| Baseline RAASi use | Baseline SGLT2i use | 172 | spearman | 0.19 | 0.012 | 0.050 |
| Baseline RAASi use | Baseline MRA use | 172 | spearman | 0.19 | 0.011 | 0.047 |
| Baseline RAASi use | Post-admission RAASi use | 172 | spearman | 0.39 | <0.001 | <0.001 |
| Baseline RAASi use | Post-admission SGLT2i use | 172 | spearman | 0.23 | 0.002 | 0.015 |
| Baseline RAASi use | Post-admission MRA use | 172 | spearman | -0.07 | 0.329 | 0.558 |
| Baseline RAASi use | 6-month serum creatinine | 77 | spearman | 0.03 | 0.802 | 0.905 |
| Baseline RAASi use | 6-month eGFR | 77 | spearman | 0.01 | 0.911 | 0.953 |
| Baseline RAASi use | 6-month UACR | 53 | spearman | -0.24 | 0.090 | 0.246 |
| Baseline RAASi use | 6-month 24-hour urinary protein excretion | 31 | spearman | 0.15 | 0.418 | 0.621 |
| Baseline RAASi use | Urine G73 | 140 | spearman | 0.02 | 0.859 | 0.938 |
| Baseline RAASi use | Urine G73/ Urine creatinine | 115 | spearman | -0.07 | 0.457 | 0.656 |
| Baseline SGLT2i use | Baseline MRA use | 172 | spearman | 0.18 | 0.016 | 0.068 |
| Baseline SGLT2i use | Post-admission RAASi use | 172 | spearman | 0.07 | 0.387 | 0.602 |
| Baseline SGLT2i use | Post-admission SGLT2i use | 172 | spearman | 0.48 | <0.001 | <0.001 |
| Baseline SGLT2i use | Post-admission MRA use | 172 | spearman | 0.07 | 0.365 | 0.595 |
| Baseline SGLT2i use | 6-month serum creatinine | 77 | spearman | 0.08 | 0.471 | 0.668 |
| Baseline SGLT2i use | 6-month eGFR | 77 | spearman | -0.04 | 0.728 | 0.854 |
| Baseline SGLT2i use | 6-month UACR | 53 | spearman | 0.00 | 1.000 | 1.000 |
| Baseline SGLT2i use | 6-month 24-hour urinary protein excretion | 31 | spearman | 0.02 | 0.909 | 0.953 |
| Baseline SGLT2i use | Urine G73 | 140 | spearman | -0.04 | 0.671 | 0.819 |
| Baseline SGLT2i use | Urine G73/ Urine creatinine | 115 | spearman | 0.01 | 0.887 | 0.944 |
| Baseline MRA use | Post-admission RAASi use | 172 | spearman | -0.09 | 0.240 | 0.450 |
| Baseline MRA use | Post-admission SGLT2i use | 172 | spearman | 0.12 | 0.116 | 0.291 |
| Baseline MRA use | Post-admission MRA use | 172 | spearman | 0.22 | 0.003 | 0.018 |
| Baseline MRA use | 6-month serum creatinine | 77 | spearman | -0.06 | 0.633 | 0.798 |
| Baseline MRA use | 6-month eGFR | 77 | spearman | 0.04 | 0.742 | 0.862 |
| Baseline MRA use | 6-month UACR | 53 | spearman | -0.10 | 0.494 | 0.692 |
| Baseline MRA use | 6-month 24-hour urinary protein excretion | 31 | spearman | 0.16 | 0.380 | 0.597 |
| Baseline MRA use | Urine G73 | 140 | spearman | 0.08 | 0.374 | 0.597 |
| Baseline MRA use | Urine G73/ Urine creatinine | 115 | spearman | 0.07 | 0.439 | 0.639 |
| Post-admission RAASi use | Post-admission SGLT2i use | 172 | spearman | 0.16 | 0.031 | 0.117 |
| Post-admission RAASi use | Post-admission MRA use | 172 | spearman | 0.07 | 0.374 | 0.597 |
| Post-admission RAASi use | 6-month serum creatinine | 77 | spearman | -0.14 | 0.212 | 0.416 |
| Post-admission RAASi use | 6-month eGFR | 77 | spearman | 0.21 | 0.069 | 0.204 |
| Post-admission RAASi use | 6-month UACR | 53 | spearman | 0.13 | 0.369 | 0.596 |
| Post-admission RAASi use | 6-month 24-hour urinary protein excretion | 31 | spearman | 0.19 | 0.299 | 0.525 |
| Post-admission RAASi use | Urine G73 | 140 | spearman | 0.11 | 0.187 | 0.382 |
| Post-admission RAASi use | Urine G73/ Urine creatinine | 115 | spearman | -0.06 | 0.533 | 0.717 |
| Post-admission SGLT2i use | Post-admission MRA use | 172 | spearman | 0.14 | 0.068 | 0.203 |
| Post-admission SGLT2i use | 6-month serum creatinine | 77 | spearman | -0.02 | 0.865 | 0.938 |
| Post-admission SGLT2i use | 6-month eGFR | 77 | spearman | 0.05 | 0.679 | 0.821 |
| Post-admission SGLT2i use | 6-month UACR | 53 | spearman | -0.09 | 0.524 | 0.711 |
| Post-admission SGLT2i use | 6-month 24-hour urinary protein excretion | 31 | spearman | -0.01 | 0.961 | 0.975 |
| Post-admission SGLT2i use | Urine G73 | 140 | spearman | 0.07 | 0.402 | 0.612 |
| Post-admission SGLT2i use | Urine G73/ Urine creatinine | 115 | spearman | 0.02 | 0.807 | 0.907 |
| Post-admission MRA use | 6-month serum creatinine | 77 | spearman | -0.16 | 0.152 | 0.343 |
| Post-admission MRA use | 6-month eGFR | 77 | spearman | 0.22 | 0.050 | 0.160 |
| Post-admission MRA use | 6-month UACR | 53 | spearman | 0.19 | 0.183 | 0.382 |
| Post-admission MRA use | 6-month 24-hour urinary protein excretion | 31 | spearman | 0.13 | 0.472 | 0.668 |
| Post-admission MRA use | Urine G73 | 140 | spearman | 0.12 | 0.165 | 0.358 |
| Post-admission MRA use | Urine G73/ Urine creatinine | 115 | spearman | 0.06 | 0.519 | 0.708 |
| 6-month serum creatinine | 6-month eGFR | 77 | spearman | -0.97 | <0.001 | <0.001 |
| 6-month serum creatinine | 6-month UACR | 51 | spearman | 0.03 | 0.819 | 0.917 |
| 6-month serum creatinine | 6-month 24-hour urinary protein excretion | 30 | spearman | 0.22 | 0.236 | 0.445 |
| 6-month serum creatinine | Urine G73 | 66 | spearman | -0.62 | <0.001 | <0.001 |
| 6-month serum creatinine | Urine G73/ Urine creatinine | 60 | spearman | -0.18 | 0.169 | 0.364 |
| 6-month eGFR | 6-month UACR | 51 | spearman | -0.07 | 0.646 | 0.803 |
| 6-month eGFR | 6-month 24-hour urinary protein excretion | 30 | spearman | -0.24 | 0.196 | 0.395 |
| 6-month eGFR | Urine G73 | 66 | spearman | 0.59 | <0.001 | <0.001 |
| 6-month eGFR | Urine G73/ Urine creatinine | 60 | spearman | 0.13 | 0.316 | 0.546 |
| 6-month UACR | 6-month 24-hour urinary protein excretion | 18 | spearman | 0.90 | <0.001 | <0.001 |
| 6-month UACR | Urine G73 | 46 | spearman | 0.15 | 0.313 | 0.542 |
| 6-month UACR | Urine G73/ Urine creatinine | 43 | spearman | 0.23 | 0.141 | 0.333 |
| 6-month 24-hour urinary protein excretion | Urine G73 | 27 | spearman | -0.16 | 0.424 | 0.626 |
| 6-month 24-hour urinary protein excretion | Urine G73/ Urine creatinine | 26 | spearman | 0.08 | 0.692 | 0.824 |
| Urine G73 | Urine G73/ Urine creatinine | 144 | spearman | 0.64 | <0.001 | <0.001 |

**Appendix Table 3. Exploratory 6-month follow-up analysis results**

| outcome | model | Variables | $\beta(95\%CI)$ | *P*-value | $R^{2}$ | AIC |
| --- | --- | --- | --- | --- | --- | --- |
| six_month_24h_proteinuria_change | Model 1: disease group | Disease groupCKD 4-5 | 260.96 (-2918.26-3440.18) | 0.867 | 0.10 | 547.56 |
| six_month_24h_proteinuria_change | Model 1: disease group | Disease groupCKD 5D | 1173.47 (-1740.33-4087.28) | 0.415 | 0.10 | 547.56 |
| six_month_24h_proteinuria_change | Model 1: disease group | Disease groupAKI/AonC | -1809.14 (-4988.36-1370.08) | 0.252 | 0.10 | 547.56 |
| six_month_24h_proteinuria_change | Model 2: + serum G73 | Disease groupCKD 4-5 | 560.90 (-2667.13-3788.94) | 0.723 | 0.14 | 548.19 |
| six_month_24h_proteinuria_change | Model 2: + serum G73 | Disease groupCKD 5D | 780.92 (-2226.21-3788.05) | 0.597 | 0.14 | 548.19 |
| six_month_24h_proteinuria_change | Model 2: + serum G73 | Disease groupAKI/AonC | -1914.46 (-5097.09-1268.16) | 0.226 | 0.14 | 548.19 |
| six_month_24h_proteinuria_change | Model 2: + serum G73 | Serum G73 | 15.18 (-13.99-44.35) | 0.293 | 0.14 | 548.19 |
| six_month_24h_proteinuria_change | Model 3: + age + sex + BMI | Disease groupCKD 4-5 | 1687.48 (-2833.60-6208.55) | 0.446 | 0.17 | 553.25 |
| six_month_24h_proteinuria_change | Model 3: + age + sex + BMI | Disease groupCKD 5D | 1337.57 (-2149.81-4824.95) | 0.434 | 0.17 | 553.25 |
| six_month_24h_proteinuria_change | Model 3: + age + sex + BMI | Disease groupAKI/AonC | -860.31 (-5233.20-3512.58) | 0.687 | 0.17 | 553.25 |
| six_month_24h_proteinuria_change | Model 3: + age + sex + BMI | Serum G73 | 17.41 (-15.38-50.20) | 0.282 | 0.17 | 553.25 |
| six_month_24h_proteinuria_change | Model 3: + age + sex + BMI | Age | -41.05 (-146.15-64.04) | 0.426 | 0.17 | 553.25 |
| six_month_24h_proteinuria_change | Model 3: + age + sex + BMI | GenderMale | -215.72 (-2700.50-2269.06) | 0.858 | 0.17 | 553.25 |
| six_month_24h_proteinuria_change | Model 3: + age + sex + BMI | BMI | 34.65 (-277.99-347.29) | 0.820 | 0.17 | 553.25 |
| six_month_24h_proteinuria_change | Model 4: + CKD duration + ALT + AST + ALB | Disease groupCKD 4-5 | 1192.46 (-3483.53-5868.44) | 0.593 | 0.51 | 494.59 |
| six_month_24h_proteinuria_change | Model 4: + CKD duration + ALT + AST + ALB | Disease groupCKD 5D | 1650.02 (-2971.97-6272.02) | 0.457 | 0.51 | 494.59 |
| six_month_24h_proteinuria_change | Model 4: + CKD duration + ALT + AST + ALB | Disease groupAKI/AonC | -1663.88 (-7222.35-3894.60) | 0.531 | 0.51 | 494.59 |
| six_month_24h_proteinuria_change | Model 4: + CKD duration + ALT + AST + ALB | Serum G73 | 18.56 (-19.52-56.64) | 0.314 | 0.51 | 494.59 |
| six_month_24h_proteinuria_change | Model 4: + CKD duration + ALT + AST + ALB | Age | 24.17 (-101.03-149.37) | 0.685 | 0.51 | 494.59 |
| six_month_24h_proteinuria_change | Model 4: + CKD duration + ALT + AST + ALB | GenderMale | -427.48 (-3359.34-2504.38) | 0.759 | 0.51 | 494.59 |
| six_month_24h_proteinuria_change | Model 4: + CKD duration + ALT + AST + ALB | BMI | 8.55 (-369.24-386.34) | 0.962 | 0.51 | 494.59 |
| six_month_24h_proteinuria_change | Model 4: + CKD duration + ALT + AST + ALB | CKD duration | -110.12 (-394.00-173.77) | 0.419 | 0.51 | 494.59 |
| six_month_24h_proteinuria_change | Model 4: + CKD duration + ALT + AST + ALB | ALT | 46.34 (-183.02-275.69) | 0.671 | 0.51 | 494.59 |
| six_month_24h_proteinuria_change | Model 4: + CKD duration + ALT + AST + ALB | AST | -65.58 (-483.82-352.67) | 0.742 | 0.51 | 494.59 |
| six_month_24h_proteinuria_change | Model 4: + CKD duration + ALT + AST + ALB | ALB | 241.17 (25.75-456.59) | 0.031 | 0.51 | 494.59 |
| six_month_24h_proteinuria_change | Model 5: + hypertension + diabetes | Disease groupCKD 4-5 | -866.89 (-6716.18-4982.39) | 0.750 | 0.65 | 473.26 |
| six_month_24h_proteinuria_change | Model 5: + hypertension + diabetes | Disease groupCKD 5D | 991.16 (-4493.17-6475.49) | 0.698 | 0.65 | 473.26 |
| six_month_24h_proteinuria_change | Model 5: + hypertension + diabetes | Disease groupAKI/AonC | -940.73 (-7217.15-5335.68) | 0.748 | 0.65 | 473.26 |
| six_month_24h_proteinuria_change | Model 5: + hypertension + diabetes | Serum G73 | 2.76 (-38.19-43.71) | 0.885 | 0.65 | 473.26 |
| six_month_24h_proteinuria_change | Model 5: + hypertension + diabetes | Age | -8.15 (-151.34-135.04) | 0.903 | 0.65 | 473.26 |
| six_month_24h_proteinuria_change | Model 5: + hypertension + diabetes | GenderMale | -173.67 (-3640.72-3293.39) | 0.914 | 0.65 | 473.26 |
| six_month_24h_proteinuria_change | Model 5: + hypertension + diabetes | BMI | 33.40 (-404.70-471.50) | 0.870 | 0.65 | 473.26 |
| six_month_24h_proteinuria_change | Model 5: + hypertension + diabetes | CKD duration | 15.84 (-308.77-340.45) | 0.916 | 0.65 | 473.26 |
| six_month_24h_proteinuria_change | Model 5: + hypertension + diabetes | ALT | 164.78 (-106.00-435.57) | 0.207 | 0.65 | 473.26 |
| six_month_24h_proteinuria_change | Model 5: + hypertension + diabetes | AST | -209.17 (-672.61-254.26) | 0.342 | 0.65 | 473.26 |
| six_month_24h_proteinuria_change | Model 5: + hypertension + diabetes | ALB | 187.81 (-35.86-411.48) | 0.092 | 0.65 | 473.26 |
| six_month_24h_proteinuria_change | Model 5: + hypertension + diabetes | `Hypertension`Yes | -1842.22 (-6113.62-2429.17) | 0.363 | 0.65 | 473.26 |
| six_month_24h_proteinuria_change | Model 5: + hypertension + diabetes | `Diabetes`Yes | 3899.49 (-924.44-8723.41) | 0.103 | 0.65 | 473.26 |
| six_month_24h_proteinuria_change | Model 6: + baseline RAASi/SGLT2i/MRA | Disease groupCKD 4-5 | 216.29 (-5479.38-5911.97) | 0.932 | 0.79 | 466.17 |
| six_month_24h_proteinuria_change | Model 6: + baseline RAASi/SGLT2i/MRA | Disease groupCKD 5D | 1706.03 (-3828.08-7240.14) | 0.497 | 0.79 | 466.17 |
| six_month_24h_proteinuria_change | Model 6: + baseline RAASi/SGLT2i/MRA | Disease groupAKI/AonC | -3148.01 (-9608.93-3312.90) | 0.294 | 0.79 | 466.17 |
| six_month_24h_proteinuria_change | Model 6: + baseline RAASi/SGLT2i/MRA | Serum G73 | 9.61 (-35.68-54.90) | 0.638 | 0.79 | 466.17 |
| six_month_24h_proteinuria_change | Model 6: + baseline RAASi/SGLT2i/MRA | Age | 38.10 (-112.19-188.39) | 0.575 | 0.79 | 466.17 |
| six_month_24h_proteinuria_change | Model 6: + baseline RAASi/SGLT2i/MRA | GenderMale | -1161.75 (-4583.19-2259.69) | 0.456 | 0.79 | 466.17 |
| six_month_24h_proteinuria_change | Model 6: + baseline RAASi/SGLT2i/MRA | BMI | -238.63 (-789.17-311.90) | 0.347 | 0.79 | 466.17 |
| six_month_24h_proteinuria_change | Model 6: + baseline RAASi/SGLT2i/MRA | CKD duration | -134.05 (-477.99-209.89) | 0.395 | 0.79 | 466.17 |
| six_month_24h_proteinuria_change | Model 6: + baseline RAASi/SGLT2i/MRA | ALT | 7.98 (-336.58-352.55) | 0.959 | 0.79 | 466.17 |
| six_month_24h_proteinuria_change | Model 6: + baseline RAASi/SGLT2i/MRA | AST | 70.04 (-488.37-628.44) | 0.780 | 0.79 | 466.17 |
| six_month_24h_proteinuria_change | Model 6: + baseline RAASi/SGLT2i/MRA | ALB | 116.15 (-140.99-373.30) | 0.328 | 0.79 | 466.17 |
| six_month_24h_proteinuria_change | Model 6: + baseline RAASi/SGLT2i/MRA | `Hypertension`Yes | -1181.75 (-5475.12-3111.63) | 0.543 | 0.79 | 466.17 |
| six_month_24h_proteinuria_change | Model 6: + baseline RAASi/SGLT2i/MRA | `Diabetes`Yes | 2838.43 (-2384.01-8060.86) | 0.245 | 0.79 | 466.17 |
| six_month_24h_proteinuria_change | Model 6: + baseline RAASi/SGLT2i/MRA | Baseline RAASi useYes | 4605.60 (-1629.09-10840.30) | 0.127 | 0.79 | 466.17 |
| six_month_24h_proteinuria_change | Model 6: + baseline RAASi/SGLT2i/MRA | Baseline SGLT2i useYes | -1131.79 (-5565.95-3302.38) | 0.572 | 0.79 | 466.17 |
| six_month_24h_proteinuria_change | Model 6: + baseline RAASi/SGLT2i/MRA | Baseline MRA useYes | -5200.54 (-14136.41-3735.32) | 0.216 | 0.79 | 466.17 |
| six_month_24h_proteinuria_change | Model 7: + post-admission medication adjustment | Disease groupCKD 4-5 | 1265.97 (-5362.15-7894.10) | 0.644 | 0.87 | 460.81 |
| six_month_24h_proteinuria_change | Model 7: + post-admission medication adjustment | Disease groupCKD 5D | 1718.47 (-5015.15-8452.10) | 0.541 | 0.87 | 460.81 |
| six_month_24h_proteinuria_change | Model 7: + post-admission medication adjustment | Disease groupAKI/AonC | -2590.56 (-11482.53-6301.40) | 0.488 | 0.87 | 460.81 |
| six_month_24h_proteinuria_change | Model 7: + post-admission medication adjustment | Serum G73 | 11.94 (-43.93-67.80) | 0.606 | 0.87 | 460.81 |
| six_month_24h_proteinuria_change | Model 7: + post-admission medication adjustment | Age | 61.32 (-111.94-234.58) | 0.405 | 0.87 | 460.81 |
| six_month_24h_proteinuria_change | Model 7: + post-admission medication adjustment | GenderMale | -1929.11 (-5955.12-2096.90) | 0.273 | 0.87 | 460.81 |
| six_month_24h_proteinuria_change | Model 7: + post-admission medication adjustment | BMI | -241.38 (-1108.45-625.70) | 0.506 | 0.87 | 460.81 |
| six_month_24h_proteinuria_change | Model 7: + post-admission medication adjustment | CKD duration | -101.27 (-537.30-334.77) | 0.577 | 0.87 | 460.81 |
| six_month_24h_proteinuria_change | Model 7: + post-admission medication adjustment | ALT | -231.44 (-819.03-356.16) | 0.358 | 0.87 | 460.81 |
| six_month_24h_proteinuria_change | Model 7: + post-admission medication adjustment | AST | 232.50 (-509.23-974.23) | 0.457 | 0.87 | 460.81 |
| six_month_24h_proteinuria_change | Model 7: + post-admission medication adjustment | ALB | 123.51 (-236.20-483.22) | 0.418 | 0.87 | 460.81 |
| six_month_24h_proteinuria_change | Model 7: + post-admission medication adjustment | `Hypertension`Yes | -884.81 (-5855.24-4085.63) | 0.666 | 0.87 | 460.81 |
| six_month_24h_proteinuria_change | Model 7: + post-admission medication adjustment | `Diabetes`Yes | 2586.27 (-3548.85-8721.39) | 0.328 | 0.87 | 460.81 |
| six_month_24h_proteinuria_change | Model 7: + post-admission medication adjustment | Baseline RAASi useYes | 3253.36 (-5514.42-12021.14) | 0.384 | 0.87 | 460.81 |
| six_month_24h_proteinuria_change | Model 7: + post-admission medication adjustment | Baseline SGLT2i useYes | -1288.20 (-8337.26-5760.86) | 0.658 | 0.87 | 460.81 |
| six_month_24h_proteinuria_change | Model 7: + post-admission medication adjustment | Baseline MRA useYes | -3768.45 (-16914.37-9377.46) | 0.494 | 0.87 | 460.81 |
| six_month_24h_proteinuria_change | Model 7: + post-admission medication adjustment | Post-admission RAASi useYes | 4349.41 (-2647.56-11346.39) | 0.171 | 0.87 | 460.81 |
| six_month_24h_proteinuria_change | Model 7: + post-admission medication adjustment | Post-admission SGLT2i useYes | 1032.71 (-5462.90-7528.32) | 0.700 | 0.87 | 460.81 |
| six_month_24h_proteinuria_change | Model 7: + post-admission medication adjustment | Post-admission MRA useYes | -4965.90 (-15113.71-5181.91) | 0.264 | 0.87 | 460.81 |
| six_month_creatinine_change | Model 1: disease group | Disease groupCKD 4-5 | -34.46 (-98.00-29.08) | 0.283 | 0.11 | 929.88 |
| six_month_creatinine_change | Model 1: disease group | Disease groupCKD 5D | 9.85 (-61.64-81.35) | 0.784 | 0.11 | 929.88 |
| six_month_creatinine_change | Model 1: disease group | Disease groupAKI/AonC | -109.56 (-187.06--32.05) | 0.006 | 0.11 | 929.88 |
| six_month_creatinine_change | Model 2: + serum G73 | Disease groupCKD 4-5 | -41.39 (-106.35-23.58) | 0.208 | 0.12 | 930.77 |
| six_month_creatinine_change | Model 2: + serum G73 | Disease groupCKD 5D | -2.89 (-78.60-72.82) | 0.940 | 0.12 | 930.77 |
| six_month_creatinine_change | Model 2: + serum G73 | Disease groupAKI/AonC | -115.25 (-193.56--36.95) | 0.004 | 0.12 | 930.77 |
| six_month_creatinine_change | Model 2: + serum G73 | Serum G73 | 0.36 (-0.34-1.06) | 0.311 | 0.12 | 930.77 |
| six_month_creatinine_change | Model 3: + age + sex + BMI | Disease groupCKD 4-5 | -47.43 (-116.12-21.26) | 0.173 | 0.14 | 935.25 |
| six_month_creatinine_change | Model 3: + age + sex + BMI | Disease groupCKD 5D | -0.29 (-78.68-78.10) | 0.994 | 0.14 | 935.25 |
| six_month_creatinine_change | Model 3: + age + sex + BMI | Disease groupAKI/AonC | -111.28 (-196.37--26.19) | 0.011 | 0.14 | 935.25 |
| six_month_creatinine_change | Model 3: + age + sex + BMI | Serum G73 | 0.35 (-0.36-1.06) | 0.328 | 0.14 | 935.25 |
| six_month_creatinine_change | Model 3: + age + sex + BMI | Age | 0.23 (-1.51-1.98) | 0.792 | 0.14 | 935.25 |
| six_month_creatinine_change | Model 3: + age + sex + BMI | GenderMale | -7.63 (-58.18-42.92) | 0.764 | 0.14 | 935.25 |
| six_month_creatinine_change | Model 3: + age + sex + BMI | BMI | -3.85 (-10.68-2.98) | 0.265 | 0.14 | 935.25 |
| six_month_creatinine_change | Model 4: + CKD duration + ALT + AST + ALB | Disease groupCKD 4-5 | -52.69 (-132.93-27.54) | 0.193 | 0.20 | 801.21 |
| six_month_creatinine_change | Model 4: + CKD duration + ALT + AST + ALB | Disease groupCKD 5D | -17.24 (-117.41-82.92) | 0.731 | 0.20 | 801.21 |
| six_month_creatinine_change | Model 4: + CKD duration + ALT + AST + ALB | Disease groupAKI/AonC | -133.68 (-249.49--17.87) | 0.025 | 0.20 | 801.21 |
| six_month_creatinine_change | Model 4: + CKD duration + ALT + AST + ALB | Serum G73 | 0.18 (-0.63-1.00) | 0.657 | 0.20 | 801.21 |
| six_month_creatinine_change | Model 4: + CKD duration + ALT + AST + ALB | Age | 0.95 (-1.25-3.16) | 0.390 | 0.20 | 801.21 |
| six_month_creatinine_change | Model 4: + CKD duration + ALT + AST + ALB | GenderMale | -13.68 (-78.12-50.75) | 0.672 | 0.20 | 801.21 |
| six_month_creatinine_change | Model 4: + CKD duration + ALT + AST + ALB | BMI | -4.19 (-12.16-3.79) | 0.297 | 0.20 | 801.21 |
| six_month_creatinine_change | Model 4: + CKD duration + ALT + AST + ALB | CKD duration | -1.28 (-7.23-4.67) | 0.668 | 0.20 | 801.21 |
| six_month_creatinine_change | Model 4: + CKD duration + ALT + AST + ALB | ALT | 0.87 (-1.92-3.66) | 0.534 | 0.20 | 801.21 |
| six_month_creatinine_change | Model 4: + CKD duration + ALT + AST + ALB | AST | -4.19 (-9.56-1.18) | 0.124 | 0.20 | 801.21 |
| six_month_creatinine_change | Model 4: + CKD duration + ALT + AST + ALB | ALB | -1.69 (-6.03-2.64) | 0.437 | 0.20 | 801.21 |
| six_month_creatinine_change | Model 5: + hypertension + diabetes | Disease groupCKD 4-5 | -50.45 (-130.93-30.02) | 0.214 | 0.25 | 777.42 |
| six_month_creatinine_change | Model 5: + hypertension + diabetes | Disease groupCKD 5D | 11.96 (-92.12-116.03) | 0.818 | 0.25 | 777.42 |
| six_month_creatinine_change | Model 5: + hypertension + diabetes | Disease groupAKI/AonC | -124.98 (-247.56--2.41) | 0.046 | 0.25 | 777.42 |
| six_month_creatinine_change | Model 5: + hypertension + diabetes | Serum G73 | -0.00 (-0.84-0.84) | 0.999 | 0.25 | 777.42 |
| six_month_creatinine_change | Model 5: + hypertension + diabetes | Age | 1.91 (-0.54-4.36) | 0.124 | 0.25 | 777.42 |
| six_month_creatinine_change | Model 5: + hypertension + diabetes | GenderMale | -12.95 (-78.52-52.63) | 0.693 | 0.25 | 777.42 |
| six_month_creatinine_change | Model 5: + hypertension + diabetes | BMI | -1.66 (-10.02-6.70) | 0.692 | 0.25 | 777.42 |
| six_month_creatinine_change | Model 5: + hypertension + diabetes | CKD duration | -1.36 (-7.45-4.73) | 0.655 | 0.25 | 777.42 |
| six_month_creatinine_change | Model 5: + hypertension + diabetes | ALT | 0.83 (-2.05-3.71) | 0.566 | 0.25 | 777.42 |
| six_month_creatinine_change | Model 5: + hypertension + diabetes | AST | -5.42 (-11.12-0.28) | 0.062 | 0.25 | 777.42 |
| six_month_creatinine_change | Model 5: + hypertension + diabetes | ALB | -1.17 (-5.75-3.40) | 0.608 | 0.25 | 777.42 |
| six_month_creatinine_change | Model 5: + hypertension + diabetes | `Hypertension`Yes | -66.99 (-137.89-3.92) | 0.064 | 0.25 | 777.42 |
| six_month_creatinine_change | Model 5: + hypertension + diabetes | `Diabetes`Yes | -33.17 (-111.10-44.76) | 0.396 | 0.25 | 777.42 |
| six_month_creatinine_change | Model 6: + baseline RAASi/SGLT2i/MRA | Disease groupCKD 4-5 | -48.34 (-130.58-33.89) | 0.243 | 0.30 | 779.19 |
| six_month_creatinine_change | Model 6: + baseline RAASi/SGLT2i/MRA | Disease groupCKD 5D | 29.80 (-78.65-138.24) | 0.583 | 0.30 | 779.19 |
| six_month_creatinine_change | Model 6: + baseline RAASi/SGLT2i/MRA | Disease groupAKI/AonC | -112.37 (-239.47-14.73) | 0.082 | 0.30 | 779.19 |
| six_month_creatinine_change | Model 6: + baseline RAASi/SGLT2i/MRA | Serum G73 | 0.10 (-0.77-0.97) | 0.817 | 0.30 | 779.19 |
| six_month_creatinine_change | Model 6: + baseline RAASi/SGLT2i/MRA | Age | 1.83 (-0.63-4.29) | 0.140 | 0.30 | 779.19 |
| six_month_creatinine_change | Model 6: + baseline RAASi/SGLT2i/MRA | GenderMale | -25.16 (-92.18-41.85) | 0.453 | 0.30 | 779.19 |
| six_month_creatinine_change | Model 6: + baseline RAASi/SGLT2i/MRA | BMI | -3.77 (-12.54-5.00) | 0.392 | 0.30 | 779.19 |
| six_month_creatinine_change | Model 6: + baseline RAASi/SGLT2i/MRA | CKD duration | -2.43 (-8.70-3.84) | 0.439 | 0.30 | 779.19 |
| six_month_creatinine_change | Model 6: + baseline RAASi/SGLT2i/MRA | ALT | 0.79 (-2.17-3.74) | 0.595 | 0.30 | 779.19 |
| six_month_creatinine_change | Model 6: + baseline RAASi/SGLT2i/MRA | AST | -4.65 (-10.45-1.16) | 0.114 | 0.30 | 779.19 |
| six_month_creatinine_change | Model 6: + baseline RAASi/SGLT2i/MRA | ALB | -1.84 (-6.51-2.82) | 0.430 | 0.30 | 779.19 |
| six_month_creatinine_change | Model 6: + baseline RAASi/SGLT2i/MRA | `Hypertension`Yes | -84.03 (-158.84--9.22) | 0.029 | 0.30 | 779.19 |
| six_month_creatinine_change | Model 6: + baseline RAASi/SGLT2i/MRA | `Diabetes`Yes | -38.56 (-124.76-47.64) | 0.372 | 0.30 | 779.19 |
| six_month_creatinine_change | Model 6: + baseline RAASi/SGLT2i/MRA | Baseline RAASi useYes | 56.68 (-20.55-133.90) | 0.146 | 0.30 | 779.19 |
| six_month_creatinine_change | Model 6: + baseline RAASi/SGLT2i/MRA | Baseline SGLT2i useYes | -26.21 (-136.26-83.83) | 0.634 | 0.30 | 779.19 |
| six_month_creatinine_change | Model 6: + baseline RAASi/SGLT2i/MRA | Baseline MRA useYes | 86.09 (-121.32-293.49) | 0.408 | 0.30 | 779.19 |
| six_month_creatinine_change | Model 7: + post-admission medication adjustment | Disease groupCKD 4-5 | -36.04 (-118.84-46.75) | 0.385 | 0.36 | 779.40 |
| six_month_creatinine_change | Model 7: + post-admission medication adjustment | Disease groupCKD 5D | 41.00 (-68.35-150.35) | 0.453 | 0.36 | 779.40 |
| six_month_creatinine_change | Model 7: + post-admission medication adjustment | Disease groupAKI/AonC | -71.21 (-205.05-62.63) | 0.289 | 0.36 | 779.40 |
| six_month_creatinine_change | Model 7: + post-admission medication adjustment | Serum G73 | 0.33 (-0.59-1.25) | 0.477 | 0.36 | 779.40 |
| six_month_creatinine_change | Model 7: + post-admission medication adjustment | Age | 2.49 (-0.14-5.13) | 0.063 | 0.36 | 779.40 |
| six_month_creatinine_change | Model 7: + post-admission medication adjustment | GenderMale | -45.58 (-116.55-25.39) | 0.202 | 0.36 | 779.40 |
| six_month_creatinine_change | Model 7: + post-admission medication adjustment | BMI | -1.68 (-10.89-7.52) | 0.714 | 0.36 | 779.40 |
| six_month_creatinine_change | Model 7: + post-admission medication adjustment | CKD duration | -1.73 (-8.30-4.85) | 0.598 | 0.36 | 779.40 |
| six_month_creatinine_change | Model 7: + post-admission medication adjustment | ALT | 0.58 (-2.39-3.56) | 0.695 | 0.36 | 779.40 |
| six_month_creatinine_change | Model 7: + post-admission medication adjustment | AST | -4.82 (-10.57-0.93) | 0.098 | 0.36 | 779.40 |
| six_month_creatinine_change | Model 7: + post-admission medication adjustment | ALB | -2.06 (-6.90-2.78) | 0.396 | 0.36 | 779.40 |
| six_month_creatinine_change | Model 7: + post-admission medication adjustment | `Hypertension`Yes | -107.96 (-187.07--28.86) | 0.009 | 0.36 | 779.40 |
| six_month_creatinine_change | Model 7: + post-admission medication adjustment | `Diabetes`Yes | -53.78 (-147.25-39.68) | 0.252 | 0.36 | 779.40 |
| six_month_creatinine_change | Model 7: + post-admission medication adjustment | Baseline RAASi useYes | 33.69 (-48.19-115.56) | 0.411 | 0.36 | 779.40 |
| six_month_creatinine_change | Model 7: + post-admission medication adjustment | Baseline SGLT2i useYes | -63.31 (-187.90-61.28) | 0.311 | 0.36 | 779.40 |
| six_month_creatinine_change | Model 7: + post-admission medication adjustment | Baseline MRA useYes | 128.96 (-85.40-343.32) | 0.231 | 0.36 | 779.40 |
| six_month_creatinine_change | Model 7: + post-admission medication adjustment | Post-admission RAASi useYes | 71.31 (-15.37-157.99) | 0.104 | 0.36 | 779.40 |
| six_month_creatinine_change | Model 7: + post-admission medication adjustment | Post-admission SGLT2i useYes | 60.72 (-37.92-159.37) | 0.221 | 0.36 | 779.40 |
| six_month_creatinine_change | Model 7: + post-admission medication adjustment | Post-admission MRA useYes | -35.49 (-163.45-92.48) | 0.579 | 0.36 | 779.40 |
| six_month_egfr_change | Model 1: disease group | Disease groupCKD 4-5 | 1.07 (-5.75-7.88) | 0.756 | 0.48 | 590.62 |
| six_month_egfr_change | Model 1: disease group | Disease groupCKD 5D | 3.00 (-4.67-10.68) | 0.438 | 0.48 | 590.62 |
| six_month_egfr_change | Model 1: disease group | Disease groupAKI/AonC | 33.18 (24.86-41.49) | <0.001 | 0.48 | 590.62 |
| six_month_egfr_change | Model 2: + serum G73 | Disease groupCKD 4-5 | 1.66 (-5.33-8.65) | 0.637 | 0.48 | 591.91 |
| six_month_egfr_change | Model 2: + serum G73 | Disease groupCKD 5D | 4.10 (-4.04-12.25) | 0.319 | 0.48 | 591.91 |
| six_month_egfr_change | Model 2: + serum G73 | Disease groupAKI/AonC | 33.67 (25.24-42.09) | <0.001 | 0.48 | 591.91 |
| six_month_egfr_change | Model 2: + serum G73 | Serum G73 | -0.03 (-0.11-0.04) | 0.416 | 0.48 | 591.91 |
| six_month_egfr_change | Model 3: + age + sex + BMI | Disease groupCKD 4-5 | 2.37 (-5.03-9.77) | 0.525 | 0.49 | 596.62 |
| six_month_egfr_change | Model 3: + age + sex + BMI | Disease groupCKD 5D | 4.00 (-4.44-12.45) | 0.348 | 0.49 | 596.62 |
| six_month_egfr_change | Model 3: + age + sex + BMI | Disease groupAKI/AonC | 33.55 (24.38-42.72) | <0.001 | 0.49 | 596.62 |
| six_month_egfr_change | Model 3: + age + sex + BMI | Serum G73 | -0.03 (-0.11-0.05) | 0.429 | 0.49 | 596.62 |
| six_month_egfr_change | Model 3: + age + sex + BMI | Age | -0.04 (-0.22-0.15) | 0.700 | 0.49 | 596.62 |
| six_month_egfr_change | Model 3: + age + sex + BMI | GenderMale | 1.00 (-4.44-6.45) | 0.714 | 0.49 | 596.62 |
| six_month_egfr_change | Model 3: + age + sex + BMI | BMI | 0.36 (-0.38-1.09) | 0.336 | 0.49 | 596.62 |
| six_month_egfr_change | Model 4: + CKD duration + ALT + AST + ALB | Disease groupCKD 4-5 | 0.90 (-7.14-8.93) | 0.824 | 0.42 | 506.62 |
| six_month_egfr_change | Model 4: + CKD duration + ALT + AST + ALB | Disease groupCKD 5D | 3.03 (-6.99-13.06) | 0.546 | 0.42 | 506.62 |
| six_month_egfr_change | Model 4: + CKD duration + ALT + AST + ALB | Disease groupAKI/AonC | 26.29 (14.69-37.88) | <0.001 | 0.42 | 506.62 |
| six_month_egfr_change | Model 4: + CKD duration + ALT + AST + ALB | Serum G73 | -0.04 (-0.12-0.05) | 0.373 | 0.42 | 506.62 |
| six_month_egfr_change | Model 4: + CKD duration + ALT + AST + ALB | Age | -0.03 (-0.25-0.20) | 0.821 | 0.42 | 506.62 |
| six_month_egfr_change | Model 4: + CKD duration + ALT + AST + ALB | GenderMale | -1.64 (-8.09-4.81) | 0.613 | 0.42 | 506.62 |
| six_month_egfr_change | Model 4: + CKD duration + ALT + AST + ALB | BMI | 0.46 (-0.34-1.26) | 0.253 | 0.42 | 506.62 |
| six_month_egfr_change | Model 4: + CKD duration + ALT + AST + ALB | CKD duration | -0.07 (-0.67-0.52) | 0.810 | 0.42 | 506.62 |
| six_month_egfr_change | Model 4: + CKD duration + ALT + AST + ALB | ALT | 0.01 (-0.27-0.29) | 0.931 | 0.42 | 506.62 |
| six_month_egfr_change | Model 4: + CKD duration + ALT + AST + ALB | AST | 0.15 (-0.39-0.68) | 0.589 | 0.42 | 506.62 |
| six_month_egfr_change | Model 4: + CKD duration + ALT + AST + ALB | ALB | -0.17 (-0.60-0.27) | 0.446 | 0.42 | 506.62 |
| six_month_egfr_change | Model 5: + hypertension + diabetes | Disease groupCKD 4-5 | 0.56 (-5.43-6.55) | 0.852 | 0.36 | 455.31 |
| six_month_egfr_change | Model 5: + hypertension + diabetes | Disease groupCKD 5D | 2.10 (-5.64-9.85) | 0.587 | 0.36 | 455.31 |
| six_month_egfr_change | Model 5: + hypertension + diabetes | Disease groupAKI/AonC | 17.47 (8.34-26.59) | <0.001 | 0.36 | 455.31 |
| six_month_egfr_change | Model 5: + hypertension + diabetes | Serum G73 | -0.03 (-0.09-0.04) | 0.396 | 0.36 | 455.31 |
| six_month_egfr_change | Model 5: + hypertension + diabetes | Age | -0.09 (-0.27-0.10) | 0.343 | 0.36 | 455.31 |
| six_month_egfr_change | Model 5: + hypertension + diabetes | GenderMale | 0.32 (-4.56-5.21) | 0.895 | 0.36 | 455.31 |
| six_month_egfr_change | Model 5: + hypertension + diabetes | BMI | 0.19 (-0.43-0.81) | 0.539 | 0.36 | 455.31 |
| six_month_egfr_change | Model 5: + hypertension + diabetes | CKD duration | -0.01 (-0.47-0.44) | 0.955 | 0.36 | 455.31 |
| six_month_egfr_change | Model 5: + hypertension + diabetes | ALT | 0.02 (-0.19-0.24) | 0.820 | 0.36 | 455.31 |
| six_month_egfr_change | Model 5: + hypertension + diabetes | AST | 0.16 (-0.26-0.59) | 0.440 | 0.36 | 455.31 |
| six_month_egfr_change | Model 5: + hypertension + diabetes | ALB | 0.05 (-0.29-0.39) | 0.778 | 0.36 | 455.31 |
| six_month_egfr_change | Model 5: + hypertension + diabetes | `Hypertension`Yes | 2.05 (-3.23-7.33) | 0.439 | 0.36 | 455.31 |
| six_month_egfr_change | Model 5: + hypertension + diabetes | `Diabetes`Yes | 3.52 (-2.28-9.32) | 0.228 | 0.36 | 455.31 |
| six_month_egfr_change | Model 6: + baseline RAASi/SGLT2i/MRA | Disease groupCKD 4-5 | 0.51 (-5.74-6.76) | 0.870 | 0.38 | 459.65 |
| six_month_egfr_change | Model 6: + baseline RAASi/SGLT2i/MRA | Disease groupCKD 5D | 0.93 (-7.31-9.18) | 0.820 | 0.38 | 459.65 |
| six_month_egfr_change | Model 6: + baseline RAASi/SGLT2i/MRA | Disease groupAKI/AonC | 16.29 (6.63-25.95) | 0.001 | 0.38 | 459.65 |
| six_month_egfr_change | Model 6: + baseline RAASi/SGLT2i/MRA | Serum G73 | -0.03 (-0.09-0.04) | 0.408 | 0.38 | 459.65 |
| six_month_egfr_change | Model 6: + baseline RAASi/SGLT2i/MRA | Age | -0.09 (-0.27-0.10) | 0.353 | 0.38 | 459.65 |
| six_month_egfr_change | Model 6: + baseline RAASi/SGLT2i/MRA | GenderMale | 0.78 (-4.31-5.87) | 0.759 | 0.38 | 459.65 |
| six_month_egfr_change | Model 6: + baseline RAASi/SGLT2i/MRA | BMI | 0.26 (-0.40-0.93) | 0.428 | 0.38 | 459.65 |
| six_month_egfr_change | Model 6: + baseline RAASi/SGLT2i/MRA | CKD duration | 0.04 (-0.43-0.52) | 0.858 | 0.38 | 459.65 |
| six_month_egfr_change | Model 6: + baseline RAASi/SGLT2i/MRA | ALT | 0.03 (-0.19-0.25) | 0.790 | 0.38 | 459.65 |
| six_month_egfr_change | Model 6: + baseline RAASi/SGLT2i/MRA | AST | 0.14 (-0.30-0.59) | 0.514 | 0.38 | 459.65 |
| six_month_egfr_change | Model 6: + baseline RAASi/SGLT2i/MRA | ALB | 0.06 (-0.29-0.41) | 0.734 | 0.38 | 459.65 |
| six_month_egfr_change | Model 6: + baseline RAASi/SGLT2i/MRA | `Hypertension`Yes | 2.79 (-2.90-8.47) | 0.329 | 0.38 | 459.65 |
| six_month_egfr_change | Model 6: + baseline RAASi/SGLT2i/MRA | `Diabetes`Yes | 4.72 (-1.83-11.27) | 0.154 | 0.38 | 459.65 |
| six_month_egfr_change | Model 6: + baseline RAASi/SGLT2i/MRA | Baseline RAASi useYes | -1.33 (-7.20-4.54) | 0.651 | 0.38 | 459.65 |
| six_month_egfr_change | Model 6: + baseline RAASi/SGLT2i/MRA | Baseline SGLT2i useYes | -1.33 (-9.69-7.03) | 0.750 | 0.38 | 459.65 |
| six_month_egfr_change | Model 6: + baseline RAASi/SGLT2i/MRA | Baseline MRA useYes | -5.01 (-20.78-10.75) | 0.525 | 0.38 | 459.65 |
| six_month_egfr_change | Model 7: + post-admission medication adjustment | Disease groupCKD 4-5 | -0.34 (-6.65-5.97) | 0.914 | 0.43 | 460.13 |
| six_month_egfr_change | Model 7: + post-admission medication adjustment | Disease groupCKD 5D | -0.09 (-8.41-8.24) | 0.984 | 0.43 | 460.13 |
| six_month_egfr_change | Model 7: + post-admission medication adjustment | Disease groupAKI/AonC | 13.13 (2.94-23.33) | 0.013 | 0.43 | 460.13 |
| six_month_egfr_change | Model 7: + post-admission medication adjustment | Serum G73 | -0.05 (-0.12-0.02) | 0.188 | 0.43 | 460.13 |
| six_month_egfr_change | Model 7: + post-admission medication adjustment | Age | -0.13 (-0.33-0.07) | 0.212 | 0.43 | 460.13 |
| six_month_egfr_change | Model 7: + post-admission medication adjustment | GenderMale | 2.24 (-3.16-7.65) | 0.407 | 0.43 | 460.13 |
| six_month_egfr_change | Model 7: + post-admission medication adjustment | BMI | 0.14 (-0.56-0.84) | 0.697 | 0.43 | 460.13 |
| six_month_egfr_change | Model 7: + post-admission medication adjustment | CKD duration | 0.00 (-0.50-0.50) | 0.988 | 0.43 | 460.13 |
| six_month_egfr_change | Model 7: + post-admission medication adjustment | ALT | 0.04 (-0.18-0.27) | 0.700 | 0.43 | 460.13 |
| six_month_egfr_change | Model 7: + post-admission medication adjustment | AST | 0.15 (-0.28-0.59) | 0.480 | 0.43 | 460.13 |
| six_month_egfr_change | Model 7: + post-admission medication adjustment | ALB | 0.08 (-0.29-0.45) | 0.671 | 0.43 | 460.13 |
| six_month_egfr_change | Model 7: + post-admission medication adjustment | `Hypertension`Yes | 4.37 (-1.66-10.39) | 0.151 | 0.43 | 460.13 |
| six_month_egfr_change | Model 7: + post-admission medication adjustment | `Diabetes`Yes | 6.02 (-1.10-13.14) | 0.095 | 0.43 | 460.13 |
| six_month_egfr_change | Model 7: + post-admission medication adjustment | Baseline RAASi useYes | 0.15 (-6.08-6.39) | 0.961 | 0.43 | 460.13 |
| six_month_egfr_change | Model 7: + post-admission medication adjustment | Baseline SGLT2i useYes | 2.00 (-7.49-11.49) | 0.673 | 0.43 | 460.13 |
| six_month_egfr_change | Model 7: + post-admission medication adjustment | Baseline MRA useYes | -7.92 (-24.24-8.41) | 0.333 | 0.43 | 460.13 |
| six_month_egfr_change | Model 7: + post-admission medication adjustment | Post-admission RAASi useYes | -4.63 (-11.23-1.98) | 0.165 | 0.43 | 460.13 |
| six_month_egfr_change | Model 7: + post-admission medication adjustment | Post-admission SGLT2i useYes | -5.32 (-12.84-2.19) | 0.160 | 0.43 | 460.13 |
| six_month_egfr_change | Model 7: + post-admission medication adjustment | Post-admission MRA useYes | 1.84 (-7.91-11.58) | 0.706 | 0.43 | 460.13 |
| six_month_uacr_change | Model 1: disease group | Disease groupCKD 4-5 | 348.94 (-835.35-1533.23) | 0.555 | 0.02 | 811.96 |
| six_month_uacr_change | Model 1: disease group | Disease groupAKI/AonC | -575.30 (-2242.64-1092.04) | 0.490 | 0.02 | 811.96 |
| six_month_uacr_change | Model 2: + serum G73 | Disease groupCKD 4-5 | 314.11 (-916.28-1544.49) | 0.609 | 0.02 | 813.89 |
| six_month_uacr_change | Model 2: + serum G73 | Disease groupAKI/AonC | -600.58 (-2299.70-1098.54) | 0.480 | 0.02 | 813.89 |
| six_month_uacr_change | Model 2: + serum G73 | Serum G73 | 1.81 (-12.74-16.37) | 0.803 | 0.02 | 813.89 |
| six_month_uacr_change | Model 3: + age + sex + BMI | Disease groupCKD 4-5 | -160.51 (-1438.03-1117.02) | 0.801 | 0.16 | 813.05 |
| six_month_uacr_change | Model 3: + age + sex + BMI | Disease groupAKI/AonC | -931.92 (-2670.40-806.56) | 0.285 | 0.16 | 813.05 |
| six_month_uacr_change | Model 3: + age + sex + BMI | Serum G73 | 2.56 (-11.51-16.64) | 0.715 | 0.16 | 813.05 |
| six_month_uacr_change | Model 3: + age + sex + BMI | Age | 40.76 (7.30-74.21) | 0.018 | 0.16 | 813.05 |
| six_month_uacr_change | Model 3: + age + sex + BMI | GenderMale | 16.49 (-920.16-953.13) | 0.972 | 0.16 | 813.05 |
| six_month_uacr_change | Model 3: + age + sex + BMI | BMI | -20.54 (-151.84-110.76) | 0.753 | 0.16 | 813.05 |
| six_month_uacr_change | Model 4: + CKD duration + ALT + AST + ALB | Disease groupCKD 4-5 | -108.07 (-1439.02-1222.88) | 0.869 | 0.49 | 684.90 |
| six_month_uacr_change | Model 4: + CKD duration + ALT + AST + ALB | Disease groupAKI/AonC | -1143.83 (-3029.98-742.31) | 0.224 | 0.49 | 684.90 |
| six_month_uacr_change | Model 4: + CKD duration + ALT + AST + ALB | Serum G73 | 3.19 (-10.31-16.70) | 0.632 | 0.49 | 684.90 |
| six_month_uacr_change | Model 4: + CKD duration + ALT + AST + ALB | Age | 21.80 (-18.23-61.82) | 0.274 | 0.49 | 684.90 |
| six_month_uacr_change | Model 4: + CKD duration + ALT + AST + ALB | GenderMale | -29.46 (-1086.98-1028.05) | 0.955 | 0.49 | 684.90 |
| six_month_uacr_change | Model 4: + CKD duration + ALT + AST + ALB | BMI | 16.85 (-112.51-146.20) | 0.792 | 0.49 | 684.90 |
| six_month_uacr_change | Model 4: + CKD duration + ALT + AST + ALB | CKD duration | -11.05 (-122.53-100.43) | 0.841 | 0.49 | 684.90 |
| six_month_uacr_change | Model 4: + CKD duration + ALT + AST + ALB | ALT | 9.51 (-37.83-56.84) | 0.684 | 0.49 | 684.90 |
| six_month_uacr_change | Model 4: + CKD duration + ALT + AST + ALB | AST | -25.96 (-112.55-60.63) | 0.544 | 0.49 | 684.90 |
| six_month_uacr_change | Model 4: + CKD duration + ALT + AST + ALB | ALB | 127.89 (64.30-191.49) | <0.001 | 0.49 | 684.90 |
| six_month_uacr_change | Model 5: + hypertension + diabetes | Disease groupCKD 4-5 | 38.70 (-1342.18-1419.57) | 0.955 | 0.52 | 686.49 |
| six_month_uacr_change | Model 5: + hypertension + diabetes | Disease groupAKI/AonC | -1646.99 (-3903.43-609.44) | 0.146 | 0.52 | 686.49 |
| six_month_uacr_change | Model 5: + hypertension + diabetes | Serum G73 | 1.80 (-12.02-15.62) | 0.791 | 0.52 | 686.49 |
| six_month_uacr_change | Model 5: + hypertension + diabetes | Age | 22.60 (-20.29-65.49) | 0.289 | 0.52 | 686.49 |
| six_month_uacr_change | Model 5: + hypertension + diabetes | GenderMale | -62.81 (-1172.09-1046.46) | 0.908 | 0.52 | 686.49 |
| six_month_uacr_change | Model 5: + hypertension + diabetes | BMI | 28.67 (-108.63-165.97) | 0.671 | 0.52 | 686.49 |
| six_month_uacr_change | Model 5: + hypertension + diabetes | CKD duration | -4.40 (-117.48-108.68) | 0.937 | 0.52 | 686.49 |
| six_month_uacr_change | Model 5: + hypertension + diabetes | ALT | 4.19 (-45.10-53.49) | 0.863 | 0.52 | 686.49 |
| six_month_uacr_change | Model 5: + hypertension + diabetes | AST | -17.29 (-105.97-71.39) | 0.692 | 0.52 | 686.49 |
| six_month_uacr_change | Model 5: + hypertension + diabetes | ALB | 129.08 (63.95-194.22) | <0.001 | 0.52 | 686.49 |
| six_month_uacr_change | Model 5: + hypertension + diabetes | `Hypertension`Yes | -383.37 (-1472.43-705.68) | 0.476 | 0.52 | 686.49 |
| six_month_uacr_change | Model 5: + hypertension + diabetes | `Diabetes`Yes | 674.96 (-790.21-2140.14) | 0.352 | 0.52 | 686.49 |
| six_month_uacr_change | Model 6: + baseline RAASi/SGLT2i/MRA | Disease groupCKD 4-5 | 16.80 (-1409.43-1443.03) | 0.981 | 0.59 | 686.39 |
| six_month_uacr_change | Model 6: + baseline RAASi/SGLT2i/MRA | Disease groupAKI/AonC | -1060.78 (-3461.62-1340.06) | 0.370 | 0.59 | 686.39 |
| six_month_uacr_change | Model 6: + baseline RAASi/SGLT2i/MRA | Serum G73 | 1.39 (-13.49-16.27) | 0.849 | 0.59 | 686.39 |
| six_month_uacr_change | Model 6: + baseline RAASi/SGLT2i/MRA | Age | 24.25 (-18.74-67.23) | 0.255 | 0.59 | 686.39 |
| six_month_uacr_change | Model 6: + baseline RAASi/SGLT2i/MRA | GenderMale | 123.13 (-1001.47-1247.73) | 0.823 | 0.59 | 686.39 |
| six_month_uacr_change | Model 6: + baseline RAASi/SGLT2i/MRA | BMI | 44.41 (-95.36-184.18) | 0.518 | 0.59 | 686.39 |
| six_month_uacr_change | Model 6: + baseline RAASi/SGLT2i/MRA | CKD duration | -26.61 (-146.55-93.34) | 0.651 | 0.59 | 686.39 |
| six_month_uacr_change | Model 6: + baseline RAASi/SGLT2i/MRA | ALT | 4.52 (-44.96-54.01) | 0.852 | 0.59 | 686.39 |
| six_month_uacr_change | Model 6: + baseline RAASi/SGLT2i/MRA | AST | -21.16 (-109.88-67.56) | 0.626 | 0.59 | 686.39 |
| six_month_uacr_change | Model 6: + baseline RAASi/SGLT2i/MRA | ALB | 137.38 (71.85-202.91) | <0.001 | 0.59 | 686.39 |
| six_month_uacr_change | Model 6: + baseline RAASi/SGLT2i/MRA | `Hypertension`Yes | -304.48 (-1444.38-835.42) | 0.586 | 0.59 | 686.39 |
| six_month_uacr_change | Model 6: + baseline RAASi/SGLT2i/MRA | `Diabetes`Yes | 102.60 (-1617.51-1822.71) | 0.903 | 0.59 | 686.39 |
| six_month_uacr_change | Model 6: + baseline RAASi/SGLT2i/MRA | Baseline RAASi useYes | -1045.44 (-2287.62-196.74) | 0.095 | 0.59 | 686.39 |
| six_month_uacr_change | Model 6: + baseline RAASi/SGLT2i/MRA | Baseline SGLT2i useYes | 544.36 (-1255.58-2344.30) | 0.538 | 0.59 | 686.39 |
| six_month_uacr_change | Model 6: + baseline RAASi/SGLT2i/MRA | Baseline MRA useYes | 1808.04 (-2199.81-5815.89) | 0.360 | 0.59 | 686.39 |
| six_month_uacr_change | Model 7: + post-admission medication adjustment | Disease groupCKD 4-5 | 319.35 (-1125.33-1764.04) | 0.650 | 0.67 | 684.34 |
| six_month_uacr_change | Model 7: + post-admission medication adjustment | Disease groupAKI/AonC | -186.00 (-2969.08-2597.08) | 0.891 | 0.67 | 684.34 |
| six_month_uacr_change | Model 7: + post-admission medication adjustment | Serum G73 | 6.90 (-10.04-23.83) | 0.406 | 0.67 | 684.34 |
| six_month_uacr_change | Model 7: + post-admission medication adjustment | Age | 26.00 (-23.11-75.11) | 0.283 | 0.67 | 684.34 |
| six_month_uacr_change | Model 7: + post-admission medication adjustment | GenderMale | -205.76 (-1464.45-1052.93) | 0.737 | 0.67 | 684.34 |
| six_month_uacr_change | Model 7: + post-admission medication adjustment | BMI | 48.75 (-96.79-194.29) | 0.493 | 0.67 | 684.34 |
| six_month_uacr_change | Model 7: + post-admission medication adjustment | CKD duration | 4.48 (-149.35-158.32) | 0.952 | 0.67 | 684.34 |
| six_month_uacr_change | Model 7: + post-admission medication adjustment | ALT | 10.78 (-41.35-62.91) | 0.671 | 0.67 | 684.34 |
| six_month_uacr_change | Model 7: + post-admission medication adjustment | AST | -36.80 (-124.82-51.23) | 0.394 | 0.67 | 684.34 |
| six_month_uacr_change | Model 7: + post-admission medication adjustment | ALB | 163.06 (86.87-239.24) | <0.001 | 0.67 | 684.34 |
| six_month_uacr_change | Model 7: + post-admission medication adjustment | `Hypertension`Yes | -727.21 (-1989.22-534.80) | 0.243 | 0.67 | 684.34 |
| six_month_uacr_change | Model 7: + post-admission medication adjustment | `Diabetes`Yes | -12.74 (-2189.75-2164.28) | 0.990 | 0.67 | 684.34 |
| six_month_uacr_change | Model 7: + post-admission medication adjustment | Baseline RAASi useYes | -921.81 (-2294.52-450.90) | 0.177 | 0.67 | 684.34 |
| six_month_uacr_change | Model 7: + post-admission medication adjustment | Baseline SGLT2i useYes | -297.63 (-2363.47-1768.21) | 0.767 | 0.67 | 684.34 |
| six_month_uacr_change | Model 7: + post-admission medication adjustment | Baseline MRA useYes | 3267.13 (-1245.24-7779.51) | 0.147 | 0.67 | 684.34 |
| six_month_uacr_change | Model 7: + post-admission medication adjustment | Post-admission RAASi useYes | 889.34 (-495.71-2274.38) | 0.195 | 0.67 | 684.34 |
| six_month_uacr_change | Model 7: + post-admission medication adjustment | Post-admission SGLT2i useYes | 306.63 (-1424.74-2038.00) | 0.716 | 0.67 | 684.34 |
| six_month_uacr_change | Model 7: + post-admission medication adjustment | Post-admission MRA useYes | 950.92 (-1114.94-3016.77) | 0.348 | 0.67 | 684.34 |

**Appendix Table 4. Bootstrap results for Logistic Regression**

| **endpoint** | **model** | **n** | **Variables** | **OR** | **OR_boot_95CI** | ***P*-value** |
| --- | --- | --- | --- | --- | --- | --- |
| Healthy vs kidney disease | Model 1: raw G73 | 1000 | Serum G73 | 1.05 | 1.05 (1.03-1.09) | 0.012 |
| Healthy vs kidney disease | Model 2: raw G73 + age + sex + BMI | 1000 | Serum G73 | 1.04 | 1.04 (1.02-1.12) | 0.043 |
| Healthy vs kidney disease | Model 2: raw G73 + age + sex + BMI | 1000 | Age | 1.08 | 1.08 (1.03-1.30) | 0.004 |
| Healthy vs kidney disease | Model 2: raw G73 + age + sex + BMI | 1000 | GenderFemale | 3.43 | 3.43 (0.77-159347674.19) | 0.168 |
| Healthy vs kidney disease | Model 2: raw G73 + age + sex + BMI | 1000 | BMI | 1.06 | 1.06 (0.83-1.30) | 0.595 |
| Healthy vs kidney disease | Model 3: Model 2 + ALT + AST + ALB | 1000 | Serum G73 | 1.04 | 1.04 (1.01-137214.90) | 0.081 |
| Healthy vs kidney disease | Model 3: Model 2 + ALT + AST + ALB | 1000 | Age | 1.04 | 1.04 (0.01-76.82) | 0.416 |
| Healthy vs kidney disease | Model 3: Model 2 + ALT + AST + ALB | 1000 | GenderFemale | 1.57 | 1.57 (0.00-3241088739161129843426280606006024888668460000268086228028862828424820.00) | 0.722 |
| Healthy vs kidney disease | Model 3: Model 2 + ALT + AST + ALB | 1000 | BMI | 1.14 | 1.14 (0.01-37050543741691396664626442422080680808264244688246420262660482.00) | 0.514 |
| Healthy vs kidney disease | Model 3: Model 2 + ALT + AST + ALB | 1000 | ALT | 0.98 | 0.98 (0.00-62.94) | 0.774 |
| Healthy vs kidney disease | Model 3: Model 2 + ALT + AST + ALB | 1000 | AST | 1.02 | 1.02 (0.00-3522320606.08) | 0.843 |
| Healthy vs kidney disease | Model 3: Model 2 + ALT + AST + ALB | 1000 | ALB | 0.58 | 0.58 (0.00-0.71) | 0.006 |
| Advanced CKD | Model 1: raw G73 | 1000 | Serum G73 | 1.02 | 1.02 (1.01-1.04) | <0.001 |
| Advanced CKD | Model 2: + age + sex + BMI | 1000 | Serum G73 | 1.02 | 1.02 (1.01-1.04) | <0.001 |
| Advanced CKD | Model 2: + age + sex + BMI | 1000 | Age | 1.04 | 1.04 (1.01-1.08) | 0.010 |
| Advanced CKD | Model 2: + age + sex + BMI | 1000 | GenderFemale | 0.77 | 0.77 (0.31-1.62) | 0.527 |
| Advanced CKD | Model 2: + age + sex + BMI | 1000 | BMI | 1.06 | 1.06 (0.93-1.23) | 0.335 |
| Advanced CKD | Model 3: + hypertension + diabetes + CKD duration | 1000 | Serum G73 | 1.02 | 1.02 (1.01-1.04) | <0.001 |
| Advanced CKD | Model 3: + hypertension + diabetes + CKD duration | 1000 | Age | 1.02 | 1.02 (0.98-1.06) | 0.284 |
| Advanced CKD | Model 3: + hypertension + diabetes + CKD duration | 1000 | GenderFemale | 1.10 | 1.10 (0.41-2.99) | 0.830 |
| Advanced CKD | Model 3: + hypertension + diabetes + CKD duration | 1000 | BMI | 1.01 | 1.01 (0.88-1.19) | 0.845 |
| Advanced CKD | Model 3: + hypertension + diabetes + CKD duration | 1000 | `Hypertension`1 | 4.77 | 4.77 (1.68-22.75) | 0.002 |
| Advanced CKD | Model 3: + hypertension + diabetes + CKD duration | 1000 | `Diabetes`1 | 1.83 | 1.83 (0.64-6.38) | 0.224 |
| Advanced CKD | Model 3: + hypertension + diabetes + CKD duration | 1000 | CKD duration | 1.08 | 1.08 (1.00-1.22) | 0.071 |
| Advanced CKD | Model 4: + ALT + AST + ALB | 1000 | Serum G73 | 1.03 | 1.03 (1.02-1.06) | <0.001 |
| Advanced CKD | Model 4: + ALT + AST + ALB | 1000 | Age | 1.03 | 1.03 (0.99-1.09) | 0.132 |
| Advanced CKD | Model 4: + ALT + AST + ALB | 1000 | GenderFemale | 1.40 | 1.40 (0.48-4.93) | 0.500 |
| Advanced CKD | Model 4: + ALT + AST + ALB | 1000 | BMI | 0.99 | 0.99 (0.82-1.20) | 0.920 |
| Advanced CKD | Model 4: + ALT + AST + ALB | 1000 | `Hypertension`1 | 5.55 | 5.55 (2.02-33.70) | 0.002 |
| Advanced CKD | Model 4: + ALT + AST + ALB | 1000 | `Diabetes`1 | 1.53 | 1.53 (0.42-6.56) | 0.415 |
| Advanced CKD | Model 4: + ALT + AST + ALB | 1000 | CKD duration | 1.08 | 1.08 (0.99-1.25) | 0.082 |
| Advanced CKD | Model 4: + ALT + AST + ALB | 1000 | ALT | 1.05 | 1.05 (1.01-1.18) | 0.039 |
| Advanced CKD | Model 4: + ALT + AST + ALB | 1000 | AST | 0.92 | 0.92 (0.76-0.97) | 0.014 |
| Advanced CKD | Model 4: + ALT + AST + ALB | 1000 | ALB | 0.99 | 0.99 (0.89-1.08) | 0.772 |
| Acute vs chronic | Model 1: raw G73 | 1000 | Serum G73 | 1.01 | 1.01 (0.99-1.01) | 0.133 |
| Acute vs chronic | Model 2: + age + sex + BMI | 1000 | Serum G73 | 1.01 | 1.01 (0.99-1.02) | 0.057 |
| Acute vs chronic | Model 2: + age + sex + BMI | 1000 | Age | 1.04 | 1.04 (1.00-1.10) | 0.065 |
| Acute vs chronic | Model 2: + age + sex + BMI | 1000 | GenderFemale | 0.65 | 0.65 (0.12-1.75) | 0.450 |
| Acute vs chronic | Model 2: + age + sex + BMI | 1000 | BMI | 1.10 | 1.10 (0.94-1.30) | 0.205 |
| Acute vs chronic | Model 3a: + eGFR | 1000 | Serum G73 | 1.01 | 1.01 (0.99-1.02) | 0.056 |
| Acute vs chronic | Model 3a: + eGFR | 1000 | Age | 1.04 | 1.04 (0.99-1.12) | 0.063 |
| Acute vs chronic | Model 3a: + eGFR | 1000 | GenderFemale | 0.64 | 0.64 (0.12-1.74) | 0.439 |
| Acute vs chronic | Model 3a: + eGFR | 1000 | BMI | 1.11 | 1.11 (0.94-1.34) | 0.189 |
| Acute vs chronic | Model 3a: + eGFR | 1000 | eGFR | 1.00 | 1.00 (0.99-1.02) | 0.694 |
| Acute vs chronic | Model 3b: + creatinine | 1000 | Serum G73 | 1.01 | 1.01 (1.00-1.03) | 0.010 |
| Acute vs chronic | Model 3b: + creatinine | 1000 | Age | 1.05 | 1.05 (1.00-1.12) | 0.025 |
| Acute vs chronic | Model 3b: + creatinine | 1000 | GenderFemale | 0.48 | 0.48 (0.10-1.25) | 0.215 |
| Acute vs chronic | Model 3b: + creatinine | 1000 | BMI | 1.17 | 1.17 (0.98-1.55) | 0.058 |
| Acute vs chronic | Model 3b: + creatinine | 1000 | Creatinine | 1.00 | 1.00 (0.99-1.00) | 0.019 |

**Appendix Table 5. Bootstrap results for calibration analysis**

| **endpoint** | **model** | **calibration_group** | **predicted** | **observed** | **n** | **predicted_boot_mean** | **predicted_low** | **predicted_high** | **observed_boot_mean** | **observed_low** | **observed_high** | **boot_success_n** |
| --- | --- | --- | --- | --- | --- | --- | --- | --- | --- | --- | --- | --- |
| Healthy vs kidney disease | Model 1: raw G73 | 1.000 | 0.83 | 0.85 | 33 | 0.83 | 0.71 | 0.93 | 0.85 | 0.85 | 0.85 | 1000 |
| Healthy vs kidney disease | Model 1: raw G73 | 2.000 | 0.92 | 0.88 | 33 | 0.92 | 0.87 | 0.97 | 0.88 | 0.88 | 0.88 | 1000 |
| Healthy vs kidney disease | Model 1: raw G73 | 3.000 | 0.96 | 0.97 | 33 | 0.96 | 0.93 | 0.99 | 0.97 | 0.97 | 0.97 | 1000 |
| Healthy vs kidney disease | Model 1: raw G73 | 4.000 | 0.98 | 1.00 | 33 | 0.98 | 0.96 | 1.00 | 1.00 | 1.00 | 1.00 | 1000 |
| Healthy vs kidney disease | Model 1: raw G73 | 5.000 | 1.00 | 1.00 | 32 | 1.00 | 0.99 | 1.00 | 1.00 | 1.00 | 1.00 | 1000 |
| Healthy vs kidney disease | Model 2: raw G73 + age + sex + BMI | 1.000 | 0.77 | 0.79 | 33 | 0.75 | 0.61 | 0.88 | 0.79 | 0.76 | 0.85 | 1000 |
| Healthy vs kidney disease | Model 2: raw G73 + age + sex + BMI | 2.000 | 0.95 | 0.94 | 33 | 0.96 | 0.91 | 1.00 | 0.95 | 0.91 | 1.00 | 1000 |
| Healthy vs kidney disease | Model 2: raw G73 + age + sex + BMI | 3.000 | 0.98 | 0.97 | 33 | 0.99 | 0.96 | 1.00 | 0.96 | 0.94 | 1.00 | 1000 |
| Healthy vs kidney disease | Model 2: raw G73 + age + sex + BMI | 4.000 | 1.00 | 1.00 | 33 | 1.00 | 0.98 | 1.00 | 0.99 | 0.94 | 1.00 | 1000 |
| Healthy vs kidney disease | Model 2: raw G73 + age + sex + BMI | 5.000 | 1.00 | 1.00 | 32 | 1.00 | 1.00 | 1.00 | 1.00 | 1.00 | 1.00 | 1000 |
| Healthy vs kidney disease | Model 3: Model 2 + ALT + AST + ALB | 1.000 | 0.72 | 0.70 | 33 | 0.69 | 0.53 | 0.83 | 0.73 | 0.70 | 0.82 | 1000 |
| Healthy vs kidney disease | Model 3: Model 2 + ALT + AST + ALB | 2.000 | 0.98 | 1.00 | 33 | 0.99 | 0.97 | 1.00 | 0.99 | 0.94 | 1.00 | 1000 |
| Healthy vs kidney disease | Model 3: Model 2 + ALT + AST + ALB | 3.000 | 1.00 | 1.00 | 33 | 1.00 | 0.99 | 1.00 | 1.00 | 0.97 | 1.00 | 1000 |
| Healthy vs kidney disease | Model 3: Model 2 + ALT + AST + ALB | 4.000 | 1.00 | 1.00 | 33 | 1.00 | 1.00 | 1.00 | 1.00 | 0.94 | 1.00 | 1000 |
| Healthy vs kidney disease | Model 3: Model 2 + ALT + AST + ALB | 5.000 | 1.00 | 1.00 | 32 | 1.00 | 1.00 | 1.00 | 0.98 | 0.88 | 1.00 | 1000 |
| Advanced CKD | Model 1: raw G73 | 1.000 | 0.35 | 0.26 | 27 | 0.34 | 0.20 | 0.47 | 0.26 | 0.26 | 0.26 | 1000 |
| Advanced CKD | Model 1: raw G73 | 2.000 | 0.44 | 0.35 | 26 | 0.44 | 0.33 | 0.53 | 0.35 | 0.35 | 0.35 | 1000 |
| Advanced CKD | Model 1: raw G73 | 3.000 | 0.53 | 0.62 | 26 | 0.53 | 0.43 | 0.62 | 0.62 | 0.62 | 0.62 | 1000 |
| Advanced CKD | Model 1: raw G73 | 4.000 | 0.64 | 0.81 | 26 | 0.65 | 0.53 | 0.78 | 0.81 | 0.81 | 0.81 | 1000 |
| Advanced CKD | Model 1: raw G73 | 5.000 | 0.84 | 0.77 | 26 | 0.84 | 0.70 | 0.96 | 0.77 | 0.77 | 0.77 | 1000 |
| Advanced CKD | Model 2: + age + sex + BMI | 1.000 | 0.25 | 0.26 | 27 | 0.23 | 0.10 | 0.37 | 0.27 | 0.19 | 0.37 | 1000 |
| Advanced CKD | Model 2: + age + sex + BMI | 2.000 | 0.43 | 0.38 | 26 | 0.42 | 0.30 | 0.53 | 0.40 | 0.27 | 0.54 | 1000 |
| Advanced CKD | Model 2: + age + sex + BMI | 3.000 | 0.57 | 0.50 | 26 | 0.57 | 0.47 | 0.66 | 0.53 | 0.38 | 0.69 | 1000 |
| Advanced CKD | Model 2: + age + sex + BMI | 4.000 | 0.69 | 0.81 | 26 | 0.71 | 0.61 | 0.81 | 0.75 | 0.62 | 0.88 | 1000 |
| Advanced CKD | Model 2: + age + sex + BMI | 5.000 | 0.85 | 0.85 | 26 | 0.87 | 0.76 | 0.96 | 0.84 | 0.77 | 0.92 | 1000 |
| Advanced CKD | Model 3: + hypertension + diabetes + CKD duration | 1.000 | 0.14 | 0.11 | 27 | 0.13 | 0.05 | 0.22 | 0.13 | 0.07 | 0.26 | 1000 |
| Advanced CKD | Model 3: + hypertension + diabetes + CKD duration | 2.000 | 0.41 | 0.46 | 26 | 0.39 | 0.27 | 0.51 | 0.46 | 0.31 | 0.62 | 1000 |
| Advanced CKD | Model 3: + hypertension + diabetes + CKD duration | 3.000 | 0.60 | 0.62 | 26 | 0.60 | 0.49 | 0.72 | 0.62 | 0.46 | 0.77 | 1000 |
| Advanced CKD | Model 3: + hypertension + diabetes + CKD duration | 4.000 | 0.74 | 0.81 | 26 | 0.77 | 0.66 | 0.88 | 0.76 | 0.62 | 0.88 | 1000 |
| Advanced CKD | Model 3: + hypertension + diabetes + CKD duration | 5.000 | 0.90 | 0.81 | 26 | 0.92 | 0.84 | 0.98 | 0.83 | 0.77 | 0.92 | 1000 |
| Advanced CKD | Model 4: + ALT + AST + ALB | 1.000 | 0.10 | 0.11 | 27 | 0.08 | 0.02 | 0.18 | 0.11 | 0.04 | 0.22 | 1000 |
| Advanced CKD | Model 4: + ALT + AST + ALB | 2.000 | 0.39 | 0.35 | 26 | 0.35 | 0.21 | 0.48 | 0.41 | 0.27 | 0.58 | 1000 |
| Advanced CKD | Model 4: + ALT + AST + ALB | 3.000 | 0.60 | 0.65 | 26 | 0.61 | 0.48 | 0.74 | 0.63 | 0.46 | 0.77 | 1000 |
| Advanced CKD | Model 4: + ALT + AST + ALB | 4.000 | 0.78 | 0.81 | 26 | 0.82 | 0.71 | 0.93 | 0.79 | 0.65 | 0.92 | 1000 |
| Advanced CKD | Model 4: + ALT + AST + ALB | 5.000 | 0.93 | 0.88 | 26 | 0.95 | 0.89 | 0.99 | 0.86 | 0.77 | 0.92 | 1000 |
| Acute vs chronic | Model 1: raw G73 | 1.000 | 0.09 | 0.09 | 33 | 0.09 | 0.05 | 0.14 | 0.09 | 0.09 | 0.09 | 1000 |
| Acute vs chronic | Model 1: raw G73 | 2.000 | 0.10 | 0.00 | 33 | 0.10 | 0.06 | 0.15 | 0.05 | 0.00 | 0.27 | 1000 |
| Acute vs chronic | Model 1: raw G73 | 3.000 | 0.11 | 0.15 | 33 | 0.11 | 0.06 | 0.16 | 0.15 | 0.12 | 0.15 | 1000 |
| Acute vs chronic | Model 1: raw G73 | 4.000 | 0.12 | 0.25 | 32 | 0.12 | 0.07 | 0.17 | 0.20 | 0.00 | 0.25 | 1000 |
| Acute vs chronic | Model 1: raw G73 | 5.000 | 0.17 | 0.09 | 32 | 0.16 | 0.09 | 0.25 | 0.09 | 0.09 | 0.09 | 1000 |
| Acute vs chronic | Model 2: + age + sex + BMI | 1.000 | 0.03 | 0.03 | 33 | 0.03 | 0.00 | 0.08 | 0.06 | 0.03 | 0.12 | 1000 |
| Acute vs chronic | Model 2: + age + sex + BMI | 2.000 | 0.07 | 0.09 | 33 | 0.06 | 0.01 | 0.11 | 0.07 | 0.00 | 0.12 | 1000 |
| Acute vs chronic | Model 2: + age + sex + BMI | 3.000 | 0.11 | 0.03 | 33 | 0.10 | 0.04 | 0.15 | 0.08 | 0.00 | 0.18 | 1000 |
| Acute vs chronic | Model 2: + age + sex + BMI | 4.000 | 0.15 | 0.22 | 32 | 0.15 | 0.08 | 0.22 | 0.16 | 0.03 | 0.28 | 1000 |
| Acute vs chronic | Model 2: + age + sex + BMI | 5.000 | 0.23 | 0.22 | 32 | 0.26 | 0.14 | 0.40 | 0.23 | 0.12 | 0.34 | 1000 |
| Acute vs chronic | Model 3a: + eGFR | 1.000 | 0.03 | 0.06 | 33 | 0.03 | 0.00 | 0.07 | 0.06 | 0.03 | 0.12 | 1000 |
| Acute vs chronic | Model 3a: + eGFR | 2.000 | 0.07 | 0.06 | 33 | 0.06 | 0.01 | 0.10 | 0.07 | 0.00 | 0.15 | 1000 |
| Acute vs chronic | Model 3a: + eGFR | 3.000 | 0.11 | 0.03 | 33 | 0.10 | 0.04 | 0.15 | 0.09 | 0.00 | 0.21 | 1000 |
| Acute vs chronic | Model 3a: + eGFR | 4.000 | 0.15 | 0.19 | 32 | 0.14 | 0.08 | 0.21 | 0.15 | 0.03 | 0.28 | 1000 |
| Acute vs chronic | Model 3a: + eGFR | 5.000 | 0.23 | 0.25 | 32 | 0.26 | 0.15 | 0.39 | 0.22 | 0.09 | 0.31 | 1000 |
| Acute vs chronic | Model 3b: + creatinine | 1.000 | 0.01 | 0.03 | 33 | 0.01 | 0.00 | 0.04 | 0.03 | 0.00 | 0.09 | 1000 |
| Acute vs chronic | Model 3b: + creatinine | 2.000 | 0.04 | 0.06 | 33 | 0.04 | 0.00 | 0.09 | 0.08 | 0.03 | 0.18 | 1000 |
| Acute vs chronic | Model 3b: + creatinine | 3.000 | 0.10 | 0.12 | 33 | 0.08 | 0.02 | 0.15 | 0.09 | 0.03 | 0.15 | 1000 |
| Acute vs chronic | Model 3b: + creatinine | 4.000 | 0.15 | 0.06 | 32 | 0.15 | 0.07 | 0.22 | 0.10 | 0.00 | 0.19 | 1000 |
| Acute vs chronic | Model 3b: + creatinine | 5.000 | 0.29 | 0.31 | 32 | 0.31 | 0.18 | 0.44 | 0.29 | 0.19 | 0.34 | 1000 |

**Appendix Table 6. Bootstrap results for Youden cutoff analysis**

| **endpoint** | **model** | **n** | **events** | **B** | **bootstrap_success_n** | **apparent_AUC** | **optimism_corrected_AUC** | **apparent_calibration_intercept** | **optimism_corrected_calibration_intercept** | **apparent_calibration_slope** | **optimism_corrected_calibration_slope** |
| --- | --- | --- | --- | --- | --- | --- | --- | --- | --- | --- | --- |
| Healthy vs kidney disease | Model 1: raw G73 | 164 | 154 | 1000 | 1000 | 0.79 | 0.79 | -0.00 | -0.16 | 1.00 | 1.03 |
| Healthy vs kidney disease | Model 2: raw G73 + age + sex + BMI | 164 | 154 | 1000 | 1000 | 0.88 | 0.85 | -0.00 | 0.41 | 1.00 | 0.78 |
| Healthy vs kidney disease | Model 3: Model 2 + ALT + AST + ALB | 164 | 154 | 1000 | 1000 | 0.97 | 0.92 | -0.00 | 1.04 | 1.00 | 0.38 |
| Advanced CKD | Model 1: raw G73 | 131 | 73 | 1000 | 1000 | 0.74 | 0.75 | 0.00 | -0.02 | 1.00 | 1.07 |
| Advanced CKD | Model 2: + age + sex + BMI | 131 | 73 | 1000 | 1000 | 0.76 | 0.73 | 0.00 | 0.01 | 1.00 | 0.86 |
| Advanced CKD | Model 3: + hypertension + diabetes + CKD duration | 131 | 73 | 1000 | 1000 | 0.81 | 0.77 | 0.00 | 0.04 | 1.00 | 0.79 |
| Advanced CKD | Model 4: + ALT + AST + ALB | 131 | 73 | 1000 | 1000 | 0.85 | 0.79 | 0.00 | 0.06 | 1.00 | 0.71 |
| Acute vs chronic | Model 1: raw G73 | 163 | 19 | 1000 | 1000 | 0.59 | 0.56 | -0.00 | -0.25 | 1.00 | 0.86 |
| Acute vs chronic | Model 2: + age + sex + BMI | 163 | 19 | 1000 | 1000 | 0.71 | 0.64 | 0.00 | -0.63 | 1.00 | 0.65 |
| Acute vs chronic | Model 3a: + eGFR | 163 | 19 | 1000 | 1000 | 0.71 | 0.63 | 0.00 | -0.77 | 1.00 | 0.58 |
| Acute vs chronic | Model 3b: + creatinine | 163 | 19 | 1000 | 1000 | 0.75 | 0.70 | 0.00 | -0.36 | 1.00 | 0.78 |

**Appendix Table 7.** Sensitivity analyses of advanced CKD model

| **comparison** | **comparison_note** | **endpoint** | **model** | **term** | **estimate** | **OR_95CI** | **p.value** |
| --- | --- | --- | --- | --- | --- | --- | --- |
| Clinical + creatinine | Base model includes serum creatinine; new model adds serum G73. | Advanced CKD sensitivity | Clinical + creatinine base | Age | 1.19 | 1.19 (0.99-1.66) | 0.133 |
| Clinical + creatinine | Base model includes serum creatinine; new model adds serum G73. | Advanced CKD sensitivity | Clinical + creatinine base | GenderFemale | 2010.16 | 2010.16 (3.74-128087956534.42) | 0.105 |
| Clinical + creatinine | Base model includes serum creatinine; new model adds serum G73. | Advanced CKD sensitivity | Clinical + creatinine base | BMI | 0.65 | 0.65 (0.17-1.21) | 0.301 |
| Clinical + creatinine | Base model includes serum creatinine; new model adds serum G73. | Advanced CKD sensitivity | Clinical + creatinine base | `Hypertension`1 | 0.03 | 0.03 (0.00-3352.52) | 0.762 |
| Clinical + creatinine | Base model includes serum creatinine; new model adds serum G73. | Advanced CKD sensitivity | Clinical + creatinine base | `Diabetes`1 | 5.97 | 5.97 (0.04-126083.98) | 0.541 |
| Clinical + creatinine | Base model includes serum creatinine; new model adds serum G73. | Advanced CKD sensitivity | Clinical + creatinine base | CKD duration | 0.99 | 0.99 (0.73-1.48) | 0.930 |
| Clinical + creatinine | Base model includes serum creatinine; new model adds serum G73. | Advanced CKD sensitivity | Clinical + creatinine base | Creatinine | 1.15 | 1.15 (1.06-1.46) | 0.034 |
| Clinical + creatinine | Base model includes serum creatinine; new model adds serum G73. | Advanced CKD sensitivity | Clinical + creatinine + serum G73 | Age | 1.23 | 1.23 (0.98-2.18) | 0.180 |
| Clinical + creatinine | Base model includes serum creatinine; new model adds serum G73. | Advanced CKD sensitivity | Clinical + creatinine + serum G73 | GenderFemale | 2663.72 | 2663.72 (3.95-137228832388.72) | 0.092 |
| Clinical + creatinine | Base model includes serum creatinine; new model adds serum G73. | Advanced CKD sensitivity | Clinical + creatinine + serum G73 | BMI | 0.61 | 0.61 (0.11-1.20) | 0.300 |
| Clinical + creatinine | Base model includes serum creatinine; new model adds serum G73. | Advanced CKD sensitivity | Clinical + creatinine + serum G73 | `Hypertension`1 | 0.03 | 0.03 (0.00-5281.38) | 0.805 |
| Clinical + creatinine | Base model includes serum creatinine; new model adds serum G73. | Advanced CKD sensitivity | Clinical + creatinine + serum G73 | `Diabetes`1 | 5.52 | 5.52 (0.04-102537.45) | 0.547 |
| Clinical + creatinine | Base model includes serum creatinine; new model adds serum G73. | Advanced CKD sensitivity | Clinical + creatinine + serum G73 | CKD duration | 0.97 | 0.97 (0.73-1.48) | 0.840 |
| Clinical + creatinine | Base model includes serum creatinine; new model adds serum G73. | Advanced CKD sensitivity | Clinical + creatinine + serum G73 | Creatinine | 1.17 | 1.17 (1.06-1.59) | 0.057 |
| Clinical + creatinine | Base model includes serum creatinine; new model adds serum G73. | Advanced CKD sensitivity | Clinical + creatinine + serum G73 | Serum G73 | 1.03 | 1.03 (0.91-1.23) | 0.647 |
| Clinical + eGFR | Strict sensitivity analysis; eGFR overlaps the advanced CKD definition. | Advanced CKD sensitivity | Clinical + eGFR base | Age | 1.03 | 1.03 (0.87-1.24) | 0.707 |
| Clinical + eGFR | Strict sensitivity analysis; eGFR overlaps the advanced CKD definition. | Advanced CKD sensitivity | Clinical + eGFR base | GenderFemale | 13.43 | 13.43 (0.04-156709.55) | 0.401 |
| Clinical + eGFR | Strict sensitivity analysis; eGFR overlaps the advanced CKD definition. | Advanced CKD sensitivity | Clinical + eGFR base | BMI | 0.60 | 0.60 (0.18-1.16) | 0.241 |
| Clinical + eGFR | Strict sensitivity analysis; eGFR overlaps the advanced CKD definition. | Advanced CKD sensitivity | Clinical + eGFR base | `Hypertension`1 | 0.01 | 0.01 (0.00-8033.90) | 0.901 |
| Clinical + eGFR | Strict sensitivity analysis; eGFR overlaps the advanced CKD definition. | Advanced CKD sensitivity | Clinical + eGFR base | `Diabetes`1 | 5.83 | 5.83 (0.06-17923.27) | 0.509 |
| Clinical + eGFR | Strict sensitivity analysis; eGFR overlaps the advanced CKD definition. | Advanced CKD sensitivity | Clinical + eGFR base | CKD duration | 1.01 | 1.01 (0.76-1.40) | 0.966 |
| Clinical + eGFR | Strict sensitivity analysis; eGFR overlaps the advanced CKD definition. | Advanced CKD sensitivity | Clinical + eGFR base | eGFR | 0.52 | 0.52 (0.19-0.76) | 0.031 |
| Clinical + eGFR | Strict sensitivity analysis; eGFR overlaps the advanced CKD definition. | Advanced CKD sensitivity | Clinical + eGFR + serum G73 | Age | 1.03 | 1.03 (0.83-1.26) | 0.689 |
| Clinical + eGFR | Strict sensitivity analysis; eGFR overlaps the advanced CKD definition. | Advanced CKD sensitivity | Clinical + eGFR + serum G73 | GenderFemale | 12.39 | 12.39 (0.02-290076.59) | 0.418 |
| Clinical + eGFR | Strict sensitivity analysis; eGFR overlaps the advanced CKD definition. | Advanced CKD sensitivity | Clinical + eGFR + serum G73 | BMI | 0.60 | 0.60 (0.18-1.16) | 0.240 |
| Clinical + eGFR | Strict sensitivity analysis; eGFR overlaps the advanced CKD definition. | Advanced CKD sensitivity | Clinical + eGFR + serum G73 | `Hypertension`1 | 0.01 | 0.01 (0.00-0.09) | 0.906 |
| Clinical + eGFR | Strict sensitivity analysis; eGFR overlaps the advanced CKD definition. | Advanced CKD sensitivity | Clinical + eGFR + serum G73 | `Diabetes`1 | 5.54 | 5.54 (0.05-34734.25) | 0.516 |
| Clinical + eGFR | Strict sensitivity analysis; eGFR overlaps the advanced CKD definition. | Advanced CKD sensitivity | Clinical + eGFR + serum G73 | CKD duration | 1.00 | 1.00 (0.76-1.49) | 0.986 |
| Clinical + eGFR | Strict sensitivity analysis; eGFR overlaps the advanced CKD definition. | Advanced CKD sensitivity | Clinical + eGFR + serum G73 | eGFR | 0.51 | 0.51 (0.19-0.76) | 0.035 |
| Clinical + eGFR | Strict sensitivity analysis; eGFR overlaps the advanced CKD definition. | Advanced CKD sensitivity | Clinical + eGFR + serum G73 | Serum G73 | 1.01 | 1.01 (0.90-1.14) | 0.894 |
| Clinical + cystatin C | Base model includes cystatin C; new model adds serum G73. | Advanced CKD sensitivity | Clinical + cystatin C base | Age | 0.94 | 0.94 (0.78-1.07) | 0.400 |
| Clinical + cystatin C | Base model includes cystatin C; new model adds serum G73. | Advanced CKD sensitivity | Clinical + cystatin C base | GenderFemale | 308.63 | 308.63 (1.03-22083429.69) | 0.151 |
| Clinical + cystatin C | Base model includes cystatin C; new model adds serum G73. | Advanced CKD sensitivity | Clinical + cystatin C base | BMI | 0.64 | 0.64 (0.26-1.16) | 0.216 |
| Clinical + cystatin C | Base model includes cystatin C; new model adds serum G73. | Advanced CKD sensitivity | Clinical + cystatin C base | `Hypertension`1 | 0.34 | 0.34 (0.00-12.37) | 0.592 |
| Clinical + cystatin C | Base model includes cystatin C; new model adds serum G73. | Advanced CKD sensitivity | Clinical + cystatin C base | `Diabetes`1 | 37.00 | 37.00 (0.33-115895.63) | 0.229 |
| Clinical + cystatin C | Base model includes cystatin C; new model adds serum G73. | Advanced CKD sensitivity | Clinical + cystatin C base | CKD duration | 1.25 | 1.25 (0.91-2.18) | 0.267 |
| Clinical + cystatin C | Base model includes cystatin C; new model adds serum G73. | Advanced CKD sensitivity | Clinical + cystatin C base | Cystatin c | 24889.48 | 24889.48 (67.86-13395155976.71) | 0.033 |
| Clinical + cystatin C | Base model includes cystatin C; new model adds serum G73. | Advanced CKD sensitivity | Clinical + cystatin C + serum G73 | Age | 0.22 | 0.22 (0.00-2222629619479703702826842624842084006844064460622066204488206666286822002600468842802202082868.00) | 1.000 |
| Clinical + cystatin C | Base model includes cystatin C; new model adds serum G73. | Advanced CKD sensitivity | Clinical + cystatin C + serum G73 | GenderFemale | 2364792352166962467042846602828802068646682882.00 | 2364792352166962467042846602828802068646682882.00 (0.00-Inf) | 0.998 |
| Clinical + cystatin C | Base model includes cystatin C; new model adds serum G73. | Advanced CKD sensitivity | Clinical + cystatin C + serum G73 | BMI | 8.79 | 8.79 (0.00-12486783711976827318668028406488066860080604604884802822460804628462066842886468486680880080020886260282004624800002424846288088424684442.00) | 1.000 |
| Clinical + cystatin C | Base model includes cystatin C; new model adds serum G73. | Advanced CKD sensitivity | Clinical + cystatin C + serum G73 | `Hypertension`1 | 0.00 | 0.00 (0.00-Inf) | 0.999 |
| Clinical + cystatin C | Base model includes cystatin C; new model adds serum G73. | Advanced CKD sensitivity | Clinical + cystatin C + serum G73 | `Diabetes`1 | 769430406430429012244064406.00 | 769430406430429012244064406.00 (0.00-Inf) | 0.999 |
| Clinical + cystatin C | Base model includes cystatin C; new model adds serum G73. | Advanced CKD sensitivity | Clinical + cystatin C + serum G73 | CKD duration | 0.69 | 0.69 (0.00-16566813553940337226648824448844080668804602686008888206626282068428800886884886888.00) | 1.000 |
| Clinical + cystatin C | Base model includes cystatin C; new model adds serum G73. | Advanced CKD sensitivity | Clinical + cystatin C + serum G73 | Cystatin c | 1101564315869100735144468428404266282442608466200606042868484224048608866846420264864860606062882804848406604002680284824486620684448446288688042208248424.00 | 1101564315869100735144468428404266282442608466200606042868484224048608866846420264864860606062882804848406604002680284824486620684448446288688042208248424.00 (0.00-Inf) | 0.994 |
| Clinical + cystatin C | Base model includes cystatin C; new model adds serum G73. | Advanced CKD sensitivity | Clinical + cystatin C + serum G73 | Serum G73 | 0.10 | 0.10 (0.00-28888397719802.53) | 0.998 |
| Clinical + UACR | Base model includes albuminuria; new model adds serum G73. | Advanced CKD sensitivity | Clinical + UACR base | Age | 1.02 | 1.02 (0.98-1.07) | 0.282 |
| Clinical + UACR | Base model includes albuminuria; new model adds serum G73. | Advanced CKD sensitivity | Clinical + UACR base | GenderFemale | 1.44 | 1.44 (0.47-4.54) | 0.520 |
| Clinical + UACR | Base model includes albuminuria; new model adds serum G73. | Advanced CKD sensitivity | Clinical + UACR base | BMI | 0.88 | 0.88 (0.73-1.04) | 0.150 |
| Clinical + UACR | Base model includes albuminuria; new model adds serum G73. | Advanced CKD sensitivity | Clinical + UACR base | `Hypertension`1 | 2.82 | 2.82 (0.82-10.94) | 0.110 |
| Clinical + UACR | Base model includes albuminuria; new model adds serum G73. | Advanced CKD sensitivity | Clinical + UACR base | `Diabetes`1 | 1.94 | 1.94 (0.55-6.80) | 0.295 |
| Clinical + UACR | Base model includes albuminuria; new model adds serum G73. | Advanced CKD sensitivity | Clinical + UACR base | CKD duration | 1.01 | 1.01 (0.91-1.11) | 0.883 |
| Clinical + UACR | Base model includes albuminuria; new model adds serum G73. | Advanced CKD sensitivity | Clinical + UACR base | Albumin-to-Creatinine Ratio | 1.00 | 1.00 (1.00-1.00) | 0.056 |
| Clinical + UACR | Base model includes albuminuria; new model adds serum G73. | Advanced CKD sensitivity | Clinical + UACR + serum G73 | Age | 1.02 | 1.02 (0.98-1.07) | 0.341 |
| Clinical + UACR | Base model includes albuminuria; new model adds serum G73. | Advanced CKD sensitivity | Clinical + UACR + serum G73 | GenderFemale | 1.50 | 1.50 (0.48-4.80) | 0.486 |
| Clinical + UACR | Base model includes albuminuria; new model adds serum G73. | Advanced CKD sensitivity | Clinical + UACR + serum G73 | BMI | 0.92 | 0.92 (0.75-1.09) | 0.338 |
| Clinical + UACR | Base model includes albuminuria; new model adds serum G73. | Advanced CKD sensitivity | Clinical + UACR + serum G73 | `Hypertension`1 | 2.90 | 2.90 (0.82-11.67) | 0.111 |
| Clinical + UACR | Base model includes albuminuria; new model adds serum G73. | Advanced CKD sensitivity | Clinical + UACR + serum G73 | `Diabetes`1 | 1.85 | 1.85 (0.51-6.63) | 0.339 |
| Clinical + UACR | Base model includes albuminuria; new model adds serum G73. | Advanced CKD sensitivity | Clinical + UACR + serum G73 | CKD duration | 1.01 | 1.01 (0.91-1.12) | 0.818 |
| Clinical + UACR | Base model includes albuminuria; new model adds serum G73. | Advanced CKD sensitivity | Clinical + UACR + serum G73 | Albumin-to-Creatinine Ratio | 1.00 | 1.00 (1.00-1.00) | 0.174 |
| Clinical + UACR | Base model includes albuminuria; new model adds serum G73. | Advanced CKD sensitivity | Clinical + UACR + serum G73 | Serum G73 | 1.01 | 1.01 (1.00-1.03) | 0.153 |
| Clinical + 24h urinary protein | Base model includes 24-hour urinary protein; new model adds serum G73. | Advanced CKD sensitivity | Clinical + 24h urinary protein base | Age | 1.02 | 1.02 (0.99-1.06) | 0.261 |
| Clinical + 24h urinary protein | Base model includes 24-hour urinary protein; new model adds serum G73. | Advanced CKD sensitivity | Clinical + 24h urinary protein base | GenderFemale | 1.56 | 1.56 (0.61-4.18) | 0.358 |
| Clinical + 24h urinary protein | Base model includes 24-hour urinary protein; new model adds serum G73. | Advanced CKD sensitivity | Clinical + 24h urinary protein base | BMI | 0.92 | 0.92 (0.80-1.05) | 0.207 |
| Clinical + 24h urinary protein | Base model includes 24-hour urinary protein; new model adds serum G73. | Advanced CKD sensitivity | Clinical + 24h urinary protein base | `Hypertension`1 | 5.10 | 5.10 (1.89-15.28) | 0.002 |
| Clinical + 24h urinary protein | Base model includes 24-hour urinary protein; new model adds serum G73. | Advanced CKD sensitivity | Clinical + 24h urinary protein base | `Diabetes`1 | 1.36 | 1.36 (0.48-3.90) | 0.563 |
| Clinical + 24h urinary protein | Base model includes 24-hour urinary protein; new model adds serum G73. | Advanced CKD sensitivity | Clinical + 24h urinary protein base | CKD duration | 1.07 | 1.07 (0.99-1.18) | 0.103 |
| Clinical + 24h urinary protein | Base model includes 24-hour urinary protein; new model adds serum G73. | Advanced CKD sensitivity | Clinical + 24h urinary protein base | `24-hour urinary total protein` | 1.00 | 1.00 (1.00-1.00) | 0.007 |
| Clinical + 24h urinary protein | Base model includes 24-hour urinary protein; new model adds serum G73. | Advanced CKD sensitivity | Clinical + 24h urinary protein + serum G73 | Age | 1.02 | 1.02 (0.99-1.06) | 0.222 |
| Clinical + 24h urinary protein | Base model includes 24-hour urinary protein; new model adds serum G73. | Advanced CKD sensitivity | Clinical + 24h urinary protein + serum G73 | GenderFemale | 1.83 | 1.83 (0.67-5.33) | 0.251 |
| Clinical + 24h urinary protein | Base model includes 24-hour urinary protein; new model adds serum G73. | Advanced CKD sensitivity | Clinical + 24h urinary protein + serum G73 | BMI | 0.93 | 0.93 (0.80-1.06) | 0.282 |
| Clinical + 24h urinary protein | Base model includes 24-hour urinary protein; new model adds serum G73. | Advanced CKD sensitivity | Clinical + 24h urinary protein + serum G73 | `Hypertension`1 | 6.07 | 6.07 (2.06-20.43) | 0.002 |
| Clinical + 24h urinary protein | Base model includes 24-hour urinary protein; new model adds serum G73. | Advanced CKD sensitivity | Clinical + 24h urinary protein + serum G73 | `Diabetes`1 | 1.23 | 1.23 (0.40-3.78) | 0.711 |
| Clinical + 24h urinary protein | Base model includes 24-hour urinary protein; new model adds serum G73. | Advanced CKD sensitivity | Clinical + 24h urinary protein + serum G73 | CKD duration | 1.08 | 1.08 (0.99-1.19) | 0.091 |
| Clinical + 24h urinary protein | Base model includes 24-hour urinary protein; new model adds serum G73. | Advanced CKD sensitivity | Clinical + 24h urinary protein + serum G73 | `24-hour urinary total protein` | 1.00 | 1.00 (1.00-1.00) | 0.025 |
| Clinical + 24h urinary protein | Base model includes 24-hour urinary protein; new model adds serum G73. | Advanced CKD sensitivity | Clinical + 24h urinary protein + serum G73 | Serum G73 | 1.02 | 1.02 (1.01-1.03) | 0.001 |

**Appendix Table 8.** Sensitivity analyses of advanced CKD model

| **comparison** | **comparison_note** | **endpoint** | **model** | **cutoff_type** | **n** | **events** | **AUC** | **AUC_low** | **AUC_high** | **probability_cutoff** | **sensitivity** | **specificity** | **PPV** | **NPV** |
| --- | --- | --- | --- | --- | --- | --- | --- | --- | --- | --- | --- | --- | --- | --- |
| Clinical + creatinine | Base model includes serum creatinine; new model adds serum G73. | Advanced CKD sensitivity | Clinical + creatinine base | Model-predicted probability | 139 | 75 | 1.00 | 1.00 | 1.00 | 0.82 | 0.97 | 1.00 | 1.00 | 0.97 |
| Clinical + creatinine | Base model includes serum creatinine; new model adds serum G73. | Advanced CKD sensitivity | Clinical + creatinine + serum G73 | Model-predicted probability | 139 | 75 | 1.00 | 1.00 | 1.00 | 0.81 | 0.97 | 1.00 | 1.00 | 0.97 |
| Clinical + eGFR | Strict sensitivity analysis; eGFR overlaps the advanced CKD definition. | Advanced CKD sensitivity | Clinical + eGFR base | Model-predicted probability | 139 | 75 | 1.00 | 1.00 | 1.00 | 0.82 | 0.97 | 1.00 | 1.00 | 0.97 |
| Clinical + eGFR | Strict sensitivity analysis; eGFR overlaps the advanced CKD definition. | Advanced CKD sensitivity | Clinical + eGFR + serum G73 | Model-predicted probability | 139 | 75 | 1.00 | 1.00 | 1.00 | 0.82 | 0.97 | 1.00 | 1.00 | 0.97 |
| Clinical + cystatin C | Base model includes cystatin C; new model adds serum G73. | Advanced CKD sensitivity | Clinical + cystatin C base | Model-predicted probability | 117 | 57 | 1.00 | 0.99 | 1.00 | 0.19 | 1.00 | 0.93 | 0.93 | 1.00 |
| Clinical + cystatin C | Base model includes cystatin C; new model adds serum G73. | Advanced CKD sensitivity | Clinical + cystatin C + serum G73 | Model-predicted probability | 117 | 57 | 1.00 | 1.00 | 1.00 | 0.50 | 1.00 | 1.00 | 1.00 | 1.00 |
| Clinical + UACR | Base model includes albuminuria; new model adds serum G73. | Advanced CKD sensitivity | Clinical + UACR base | Model-predicted probability | 81 | 24 | 0.74 | 0.63 | 0.85 | 0.15 | 1.00 | 0.37 | 0.40 | 1.00 |
| Clinical + UACR | Base model includes albuminuria; new model adds serum G73. | Advanced CKD sensitivity | Clinical + UACR + serum G73 | Model-predicted probability | 81 | 24 | 0.75 | 0.63 | 0.86 | 0.20 | 0.88 | 0.51 | 0.43 | 0.91 |
| Clinical + 24h urinary protein | Base model includes 24-hour urinary protein; new model adds serum G73. | Advanced CKD sensitivity | Clinical + 24h urinary protein base | Model-predicted probability | 112 | 48 | 0.77 | 0.67 | 0.84 | 0.53 | 0.62 | 0.84 | 0.75 | 0.75 |
| Clinical + 24h urinary protein | Base model includes 24-hour urinary protein; new model adds serum G73. | Advanced CKD sensitivity | Clinical + 24h urinary protein + serum G73 | Model-predicted probability | 112 | 48 | 0.82 | 0.74 | 0.90 | 0.49 | 0.75 | 0.81 | 0.75 | 0.81 |

**Appendix Table 9.** Sensitivity analyses of advanced CKD model

| **endpoint** | **comparison** | **comparison_note** | **base_model** | **new_model** | **n** | **events** | **non_events** | **AUC_base** | **AUC_new** | **delta_AUC** | **DeLong_p** | **NRI_event** | **NRI_nonevent** | **NRI** | **IDI** | **event_up** | **event_down** | **nonevent_down** | **nonevent_up** | **discrimination_base** | **discrimination_new** |
| --- | --- | --- | --- | --- | --- | --- | --- | --- | --- | --- | --- | --- | --- | --- | --- | --- | --- | --- | --- | --- | --- |
| Advanced CKD sensitivity | Clinical + creatinine | Base model includes serum creatinine; new model adds serum G73. | Clinical + creatinine base | Clinical + creatinine + serum G73 | 139 | 75 | 64 | 1.00 | 1.00 | 0.00 | 1.000 | 0.27 | 0.69 | 0.95 | 0.00 | 0.307 | 0.04 | 0.84 | 0.156 | 0.95 | 0.95 |
| Advanced CKD sensitivity | Clinical + eGFR | Strict sensitivity analysis; eGFR overlaps the advanced CKD definition. | Clinical + eGFR base | Clinical + eGFR + serum G73 | 139 | 75 | 64 | 1.00 | 1.00 | 0.00 | 1.000 | 0.87 | 0.28 | 1.15 | -0.00 | 0.933 | 0.07 | 0.42 | 0.141 | 0.95 | 0.95 |
| Advanced CKD sensitivity | Clinical + cystatin C | Base model includes cystatin C; new model adds serum G73. | Clinical + cystatin C base | Clinical + cystatin C + serum G73 | 117 | 57 | 60 | 1.00 | 1.00 | 0.00 | 0.170 | 0.67 | 1.00 | 1.67 | 0.09 | 0.667 | 0.00 | 1.00 | <0.001 | 0.91 | 1.00 |
| Advanced CKD sensitivity | Clinical + UACR | Base model includes albuminuria; new model adds serum G73. | Clinical + UACR base | Clinical + UACR + serum G73 | 81 | 24 | 57 | 0.74 | 0.75 | 0.01 | 0.577 | 0.00 | 0.44 | 0.44 | 0.02 | 0.500 | 0.50 | 0.72 | 0.281 | 0.15 | 0.18 |
| Advanced CKD sensitivity | Clinical + 24h urinary protein | Base model includes 24-hour urinary protein; new model adds serum G73. | Clinical + 24h urinary protein base | Clinical + 24h urinary protein + serum G73 | 112 | 48 | 64 | 0.77 | 0.82 | 0.06 | 0.057 | 0.17 | 0.59 | 0.76 | 0.10 | 0.583 | 0.42 | 0.80 | 0.203 | 0.21 | 0.31 |

**Appendix Table 10.** Events-per-variable and model-stability assessment for logistic regression models

| **endpoint** | **model** | **n** | **events** | **non_events** | **predictor_variable_n** | **predictor_parameter_df** | **events_per_variable** | **non_events_per_variable** | **smaller_class_per_variable** | **events_per_parameter** | **non_events_per_parameter** | **smaller_class_per_parameter** |
| --- | --- | --- | --- | --- | --- | --- | --- | --- | --- | --- | --- | --- |
| Healthy vs kidney disease | Model 1: raw G73 | 164 | 154 | 10 | 1 | 1 | 154.00 | 10.00 | 10.00 | 154.00 | 10.00 | 10.00 |
| Healthy vs kidney disease | Model 2: raw G73 + age + sex + BMI | 164 | 154 | 10 | 4 | 4 | 38.50 | 2.50 | 2.50 | 38.50 | 2.50 | 2.50 |
| Healthy vs kidney disease | Model 3: Model 2 + ALT + AST + ALB | 164 | 154 | 10 | 7 | 7 | 22.00 | 1.43 | 1.43 | 22.00 | 1.43 | 1.43 |
| Advanced CKD | Model 1: raw G73 | 131 | 73 | 58 | 1 | 1 | 73.00 | 58.00 | 58.00 | 73.00 | 58.00 | 58.00 |
| Advanced CKD | Model 2: + age + sex + BMI | 131 | 73 | 58 | 4 | 4 | 18.25 | 14.50 | 14.50 | 18.25 | 14.50 | 14.50 |
| Advanced CKD | Model 3: + hypertension + diabetes + CKD duration | 131 | 73 | 58 | 7 | 7 | 10.43 | 8.29 | 8.29 | 10.43 | 8.29 | 8.29 |
| Advanced CKD | Model 4: + ALT + AST + ALB | 131 | 73 | 58 | 10 | 10 | 7.30 | 5.80 | 5.80 | 7.30 | 5.80 | 5.80 |
| Acute vs chronic | Model 1: raw G73 | 163 | 19 | 144 | 1 | 1 | 19.00 | 144.00 | 19.00 | 19.00 | 144.00 | 19.00 |
| Acute vs chronic | Model 2: + age + sex + BMI | 163 | 19 | 144 | 4 | 4 | 4.75 | 36.00 | 4.75 | 4.75 | 36.00 | 4.75 |
| Acute vs chronic | Model 3a: + eGFR | 163 | 19 | 144 | 5 | 5 | 3.80 | 28.80 | 3.80 | 3.80 | 28.80 | 3.80 |
| Acute vs chronic | Model 3b: + creatinine | 163 | 19 | 144 | 5 | 5 | 3.80 | 28.80 | 3.80 | 3.80 | 28.80 | 3.80 |
